# Quinazolinone–diarylpyrazole hybrids as dual EGFR/COX-2 inhibitors: antiproliferative, mechanistic, and *in silico* studies

**DOI:** 10.1039/d6ra07867b

**Published:** 2026-09-24

**Authors:** Abdullah Alkhammash, Basima A. A. Saleem, Khulood H. Oudah, Mohamed Ahmed, Kasim Sakran Abass, Sara Mahmoud Farhan, Magdi E. A. Zaki, Sobhi M. Gomha

**Affiliations:** a Department of Pharmacology, College of Pharmacy, Al-Dawadmi Campus, Shaqra University Shaqra 11961 Saudi Arabia; b Department of Chemistry, College of Science, University of Mosul Mosul 41001 Iraq; c Department of Pharmacy, Mazaya University College Nasiriyah Iraq; d College of Medicine, Dhofar University Salalah 122 Oman; e Department of Physiology, Biochemistry and Pharmacology, College of Veterinary Medicine, University of Kirkuk Kirkuk 36001 Iraq kasim_abass@uokirkuk.edu.iq; f Department of Microbiology and Immunology, Faculty of Pharmacy, Deraya University New Minia 61768 Egypt; g Department of Chemistry, Faculty of Science, Imam Mohammad Ibn Saud Islamic University (IMSIU) Riyadh 11623 Saudi Arabia; h Department of Chemistry, Faculty of Science, Islamic University of Madinah Madinah 42351 Saudi Arabia smgomha@iu.edu.sa

## Abstract

Abnormal epidermal growth factor receptor (EGFR) signaling and cyclooxygenase-2 (COX-2) mediated inflammatory pathways contribute to tumor progression and survival, supporting their simultaneous pharmacological targeting. Herein, a series of quinazolinone–diarylpyrazole hybrids 7a–l was designed, synthesized, and evaluated as dual EGFR/COX-2 inhibitors. Compound 7f exhibited the most balanced inhibitory profile against the two targets, exhibiting EGFR and COX-2 IC_50_ values of 0.24 ± 0.03 and 0.08 ± 0.01 µM, respectively, together with a COX-2 selectivity index of 197.88. Compound 7j displayed the highest EGFR inhibitory activity within the series (IC_50_ = 0.15 ± 0.02 µM). The hybrids showed marked antiproliferative activity against A431, A549, and HCA-7 cancer cells, with 7f exhibiting IC_50_ values of 1.18 ± 0.14, 1.42 ± 0.17, and 0.86 ± 0.17 µM, respectively, and a cellular selectivity index of 14.08 toward MRC-5 cells. Cellular studies further demonstrated that 7f inhibited cellular EGFR phosphorylation (IC_50_ = 0.77 ± 0.11 µM) and suppressed PGE-2 release by 89.9% at 1 µM. Moreover, 7f increased Bax expression, reduced Bcl-2 levels, and enhanced caspase-3 and caspase-9 levels, supporting induction of apoptosis. Molecular docking analyses supported favorable accommodation of 7f within the binding sites of wild-type EGFR and COX-2, consistent with its experimentally observed dual inhibitory activity. Collectively, these findings identify 7f as a promising lead displaying concurrent EGFR and COX-2 inhibitory activity together with pronounced antiproliferative effects.

## Abstract 2

Novel quinazolinone–diarylpyrazole hybrids as dual EGFR and COX-2 inhibitors.

## Graphical abstract

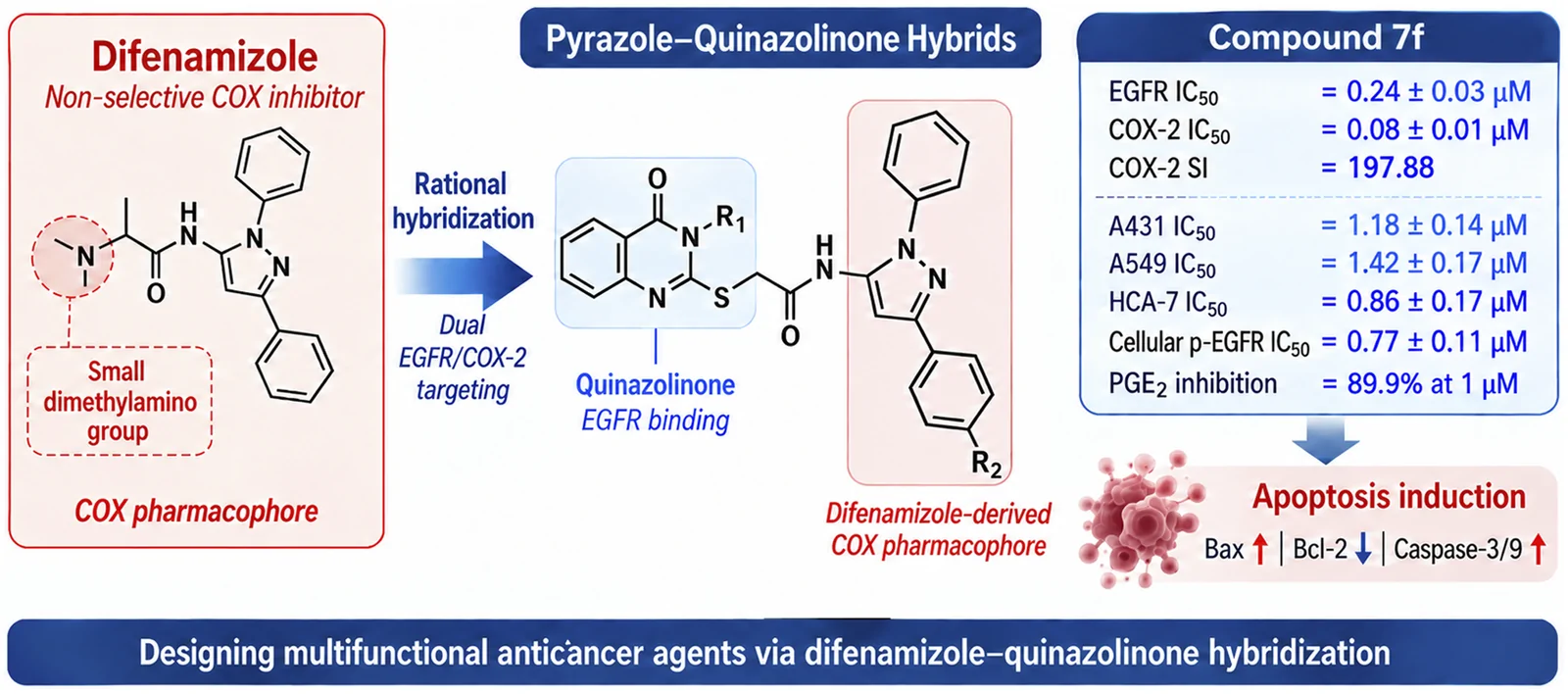

## 1. Introduction

Cancer remains a major therapeutic challenge because malignant progression is sustained by interconnected signaling networks that regulate proliferation, survival, angiogenesis, invasion, inflammation, and resistance to cell death.^1^ Although molecularly targeted therapies have markedly advanced cancer treatment, inhibition of a single signaling pathway may be undermined by compensatory mechanisms and pathway cross-talk.^2^ Consequently, the development of single molecules capable of simultaneously modulating more than one cancer-relevant target has attracted considerable interest as a strategy to enhance therapeutic efficacy while potentially limiting the pharmacokinetic complexities associated with combination regimens.^2,3^ Among the signaling systems implicated in malignant progression, the epidermal growth factor receptor (EGFR) pathway and the cyclooxygenase-2/prostaglandin E2 (COX-2/PGE-2) axis represent mechanistically distinct yet biologically interconnected targets that may be particularly suitable for a dual-targeting approach.^4^

EGFR is a receptor tyrosine kinase that plays a pivotal role in the regulation of cellular proliferation, survival, migration, and differentiation. Aberrant activation of EGFR, arising from receptor overexpression, amplification, or activating mutations, is a frequent molecular event in several malignancies and can promote persistent activation of downstream proliferative and cell-survival signaling pathways.^5^ The clinical success of small-molecule EGFR tyrosine kinase inhibitors has firmly established EGFR as a validated anticancer target.^6^ In particular, quinazoline- and quinazolinone-derived heteroaromatic systems have played a prominent role in the development of ATP-competitive EGFR inhibitors;^7^ representative examples are shown in Fig. 1.^8^ Structural studies have demonstrated the ability of quinazolinone-based inhibitors to occupy the EGFR kinase ATP-binding region, providing a well-established structural basis for the use of quinazolinone-related scaffolds in EGFR-directed drug design.^9,10^ Accordingly, incorporation of a quinazolinone-derived pharmacophore into multitarget hybrids represents an attractive approach for maintaining an EGFR-directed component while introducing additional target-modulating structural features.

**Fig. 1:**
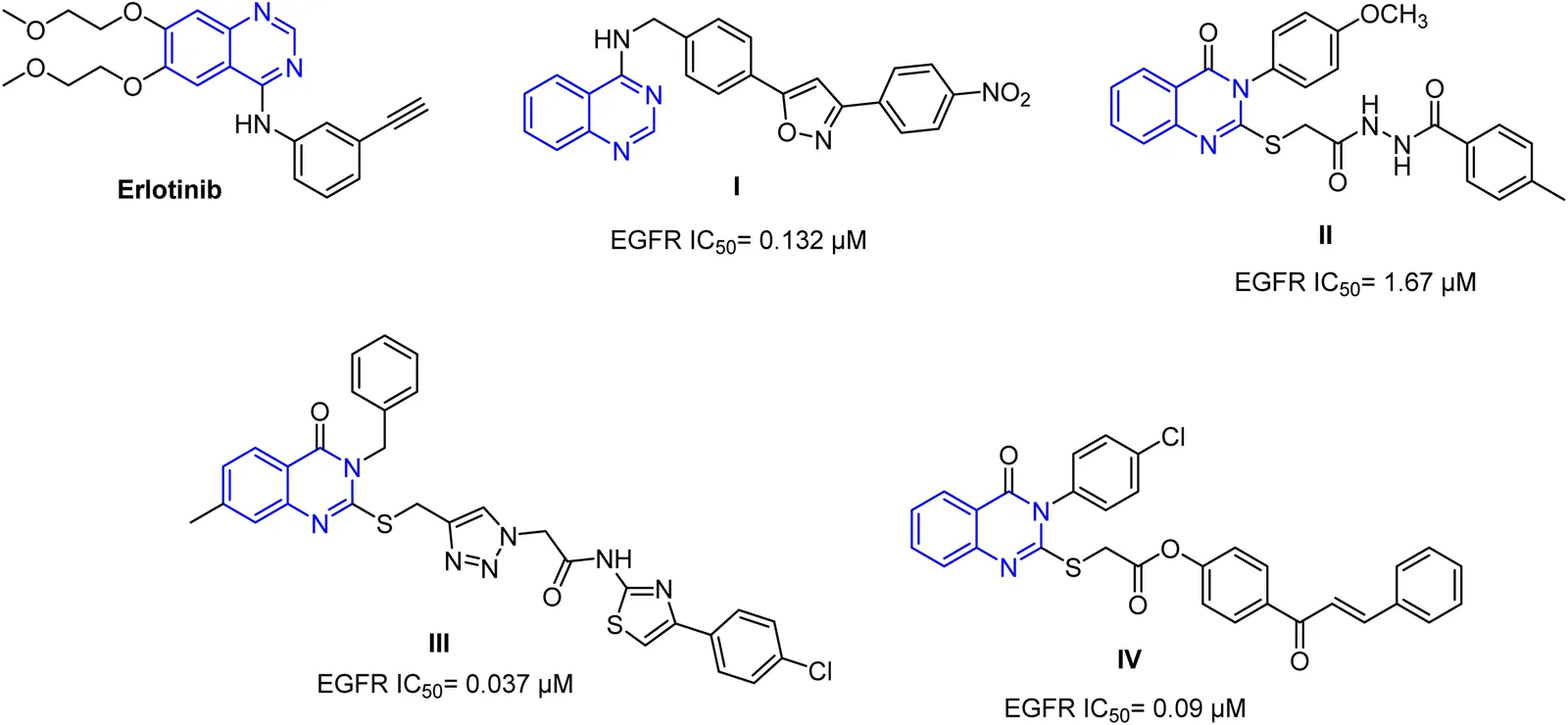
Representative quinazoline- and quinazolinone-based EGFR inhibitors.

In parallel, increasing evidence has highlighted the importance of COX-2 in cancer biology beyond its classical role in inflammation. In contrast to the constitutively expressed COX-1 isoform, which participates in several physiological homeostatic functions, COX-2 is inducible and is frequently upregulated in malignant tissues.^11,12^ COX-2-derived prostaglandins, particularly PGE_2_, can support tumor progression by promoting proliferation, angiogenesis, migration, invasion, immune evasion, and resistance to apoptosis.^13–15^ These observations have sustained interest in selective COX-2 inhibition as a complementary anticancer strategy.^16^ Importantly, structural differences between the two cyclooxygenase isoforms provide opportunities for achieving COX-2 selectivity. The COX-2 active site contains an additional secondary pocket arising partly from amino-acid substitutions, including replacement of Ile523 in COX-1 by the smaller Val523 residue in COX-2, thereby generating a larger region capable of accommodating sterically demanding substituents.^17^

Pyrazole represents one of the most versatile heterocyclic scaffolds in medicinal chemistry and has been extensively explored in the development of anti-inflammatory and COX-directed agents.^18^ Several clinically used or pharmacologically established anti-inflammatory compounds, including celecoxib, lonazolac, and difenamizole, contain a pyrazole nucleus, underscoring the relevance of this heterocycle to cyclooxygenase inhibitor design (Fig. 2).^19,20^ Of particular interest is difenamizole, which contains a 1,3-diphenylpyrazole core and behaves as a non-selective cyclooxygenase inhibitor. Previous optimization of difenamizole analogues indicated that steric expansion around this scaffold can enhance COX-2 selectivity, a trend consistent with the greater capacity of the COX-2 secondary pocket to accommodate bulkier structural features.^21^ These observations suggested that the 1,3-diphenylpyrazole framework could provide a suitable COX-directed component for incorporation into larger multitarget structures.

**Fig. 2:**
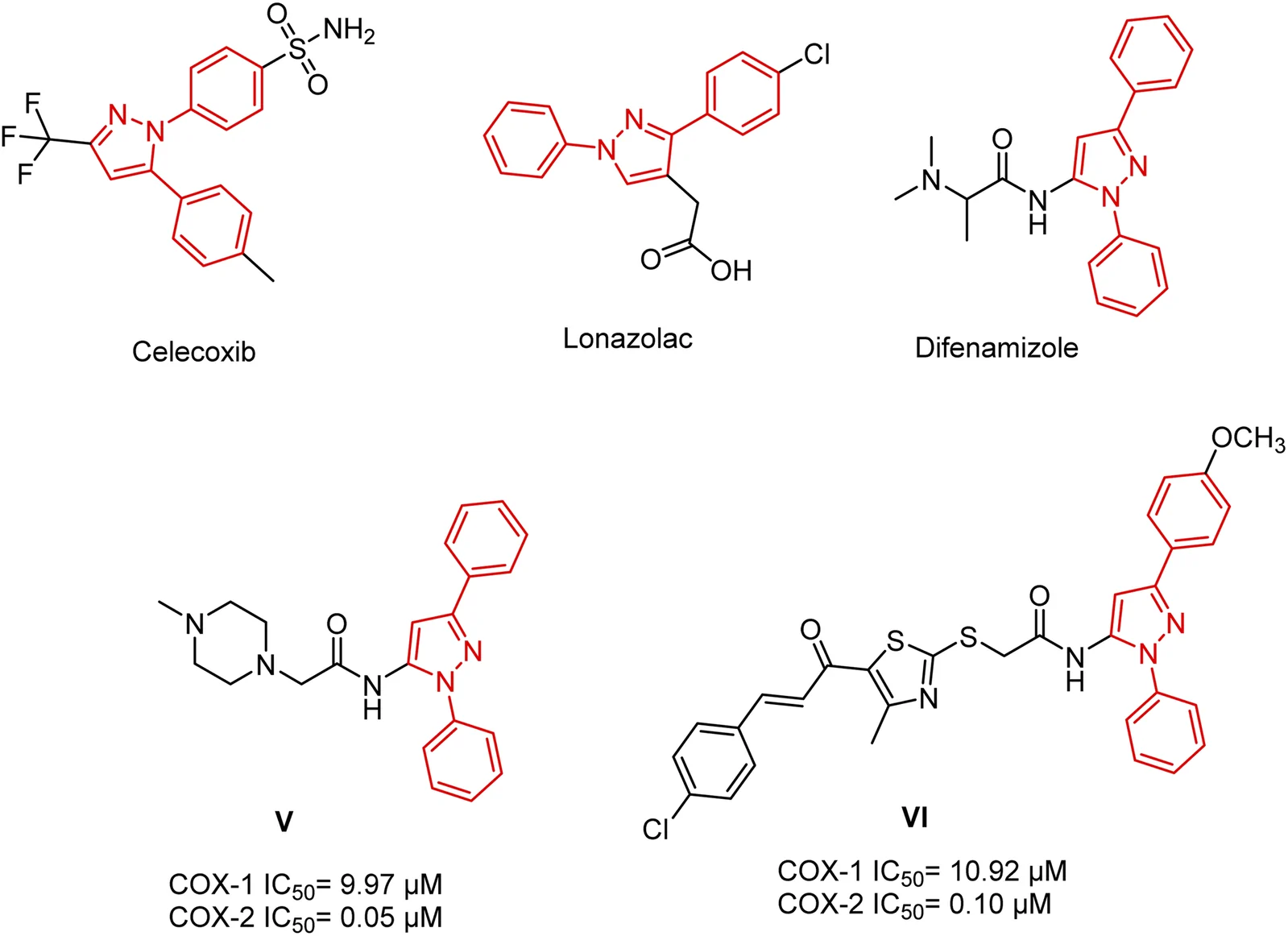
Representative pyrazole-based anti-inflammatory agents.

The rationale for simultaneously targeting EGFR and COX-2 extends beyond the independent involvement of these proteins in malignant progression. Accumulating evidence indicates substantial functional cross-talk between the two pathways. COX-2 overexpression and PGE_2_ signaling can enhance EGFR activation, whereas EGFR stimulation can in turn increase COX-2 expression, thereby establishing a positive feedback relationship capable of reinforcing tumor-promoting signaling and therapeutic resistance.^4^ More specifically, COX-2-derived PGE_2_ has been reported to stimulate EGFR phosphorylation through prostanoid receptor-mediated mechanisms and to enhance downstream MAPK and PI3K/AKT signaling, while aberrant EGFR activation can promote COX-2 transcription through inflammatory transcriptional pathways.^22–24^ This bidirectional interplay provides a biological basis for dual EGFR/COX-2 inhibition, since simultaneous interference with both pathways may suppress oncogenic kinase signaling while concurrently attenuating prostaglandin-driven tumor-promoting processes.^4,25^

Simultaneous EGFR/COX-2 inhibition is, however, an established multitarget strategy and has been explored through several structural approaches, including quinoxaline derivatives,^26^ quinazolinone-based systems targeting mutant EGFR,^27^ pyrazole/triazole scaffolds,^28^ diarylpyrazole carboxamides,^29^ and quinazoline–triazole hybrids.^30^ Recent optimization of difenamizole analogues^21^ has further demonstrated that structural expansion of this diarylpyrazole scaffold can enhance COX-2 selectivity while introducing EGFR inhibitory activity. More broadly, heterocyclic multitarget anticancer systems have combined EGFR inhibition with complementary mechanisms such as topoisomerase II inhibition and DNA intercalation,^31^ while other recent anticancer studies have employed extensive mechanistic validation through apoptosis-related markers and cancer-versus-normal-cell selectivity.^32^ Pyrazolopyrimidine scaffolds have also been explored as multitarget EGFR/STAT3-downregulatory candidates with apoptotic activity.^33^ Collectively, these studies emphasize the importance of experimentally validating the intended biological mechanisms rather than relying solely on cytotoxicity. Accordingly, the distinction of the present study lies not in the EGFR/COX-2 dual-targeting premise itself, but in the integration of a difenamizole-derived 1,3-diarylpyrazole domain with an EGFR-directed quinazolinone through a thioacetamide linker, together with biochemical and cellular evaluation of both targeted pathways.

### 1.1. Rational design

Based on these considerations, the present compounds were designed by combining a difenamizole-inspired diarylpyrazole domain with an EGFR-directed quinazolinone moiety within a single molecular framework. The 1,3-diphenylpyrazole–amide region of difenamizole was retained as the COX-directed component, whereas its relatively small terminal dimethylamino portion was replaced by a substantially bulkier quinazolinone-containing domain. This modification was intended not only to increase steric demand in a manner potentially favorable for accommodation within the enlarged COX-2 secondary pocket,^34^ but also to introduce a second pharmacophoric element capable of interacting with the EGFR kinase domain.

The quinazolinone moiety was therefore incorporated as the EGFR-directed component, based on the established importance of quinazolinone-related heterocycles in ATP-site recognition by EGFR inhibitors. The diarylpyrazole and quinazolinone domains were connected through a thioacetamide-based spacer, providing sufficient structural extension to integrate both pharmacophoric regions within the same molecule. Variation of the N-3 substituent on the quinazolinone ring and the *para* substituent of the diarylpyrazole phenyl ring was introduced to explore the influence of steric, hydrophobic, and electronic properties on EGFR inhibition, COX-2 potency and selectivity, and antiproliferative activity. This design strategy ultimately afforded the target quinazolinone–diarylpyrazole hybrids 7a–l, as illustrated in Fig. 3. Accordingly, the compounds were prospectively designed as candidate dual-target ligands; whether this design translated into measurable inhibitory activity against both EGFR and COX-2 was subsequently determined through independent biochemical assays.

**Fig. 3:**
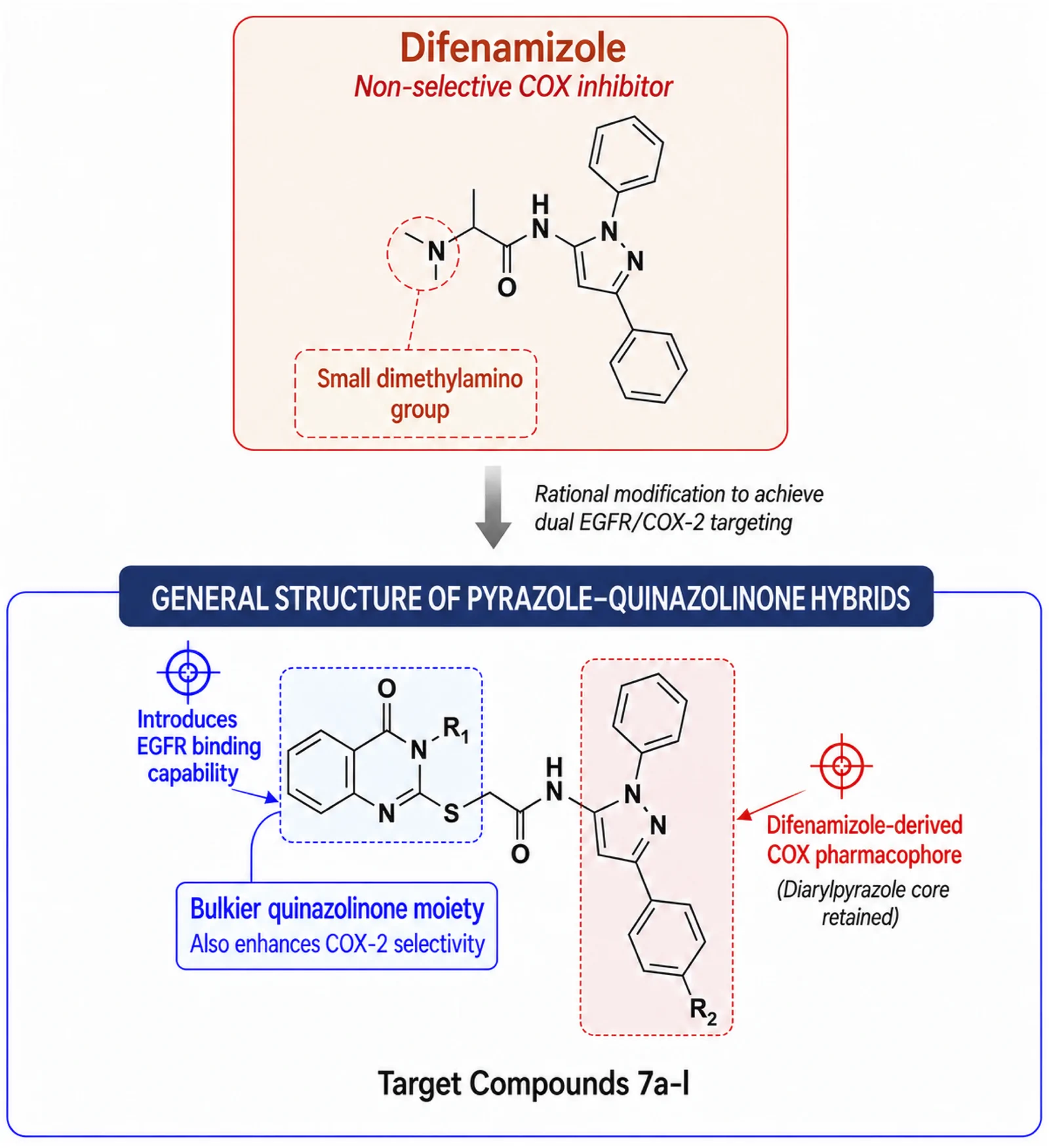
Rational design of the dual EGFR/COX-2-targeted pyrazole–quinazolinone hybrids 7a–l based on structural modification of difenamizole.

## 2. Results and discussion

### 2.1. Chemistry

The synthetic pathway employed for the preparation of the target hybrids 7a–l is depicted in Scheme 1. Initially, anthranilic acid 1 was reacted with the appropriate alkyl isothiocyanate in the presence of triethylamine (TEA) in ethanol under reflux for 2 h to afford the 3-substituted 2-mercaptoquinazolin-4(3*H*)-one derivatives 2a–c. In parallel, the appropriately substituted phenacyl bromides 3a–d were treated with KCN in ethanol at 50 °C for 2 h to furnish the corresponding nitrile intermediates 4a–d. Cyclocondensation of 4a–d with phenylhydrazine hydrochloride under reflux for 8 h afforded the 5-amino-1-phenylpyrazole derivatives 5a–d. Subsequent reaction of the amino derivatives 5a–d with chloroacetyl chloride in DCM at 0 °C for 12 h provided the chloroacetamide intermediates 6a–d. Finally, the mercaptoquinazolinone derivatives 2a–c were reacted with the appropriate chloroacetamides 6a–d in acetonitrile in the presence of TEA at room temperature for 6 h, affording the target compounds 7a–l through *S*-alkylation of the mercapto group.

**Scheme 1:**
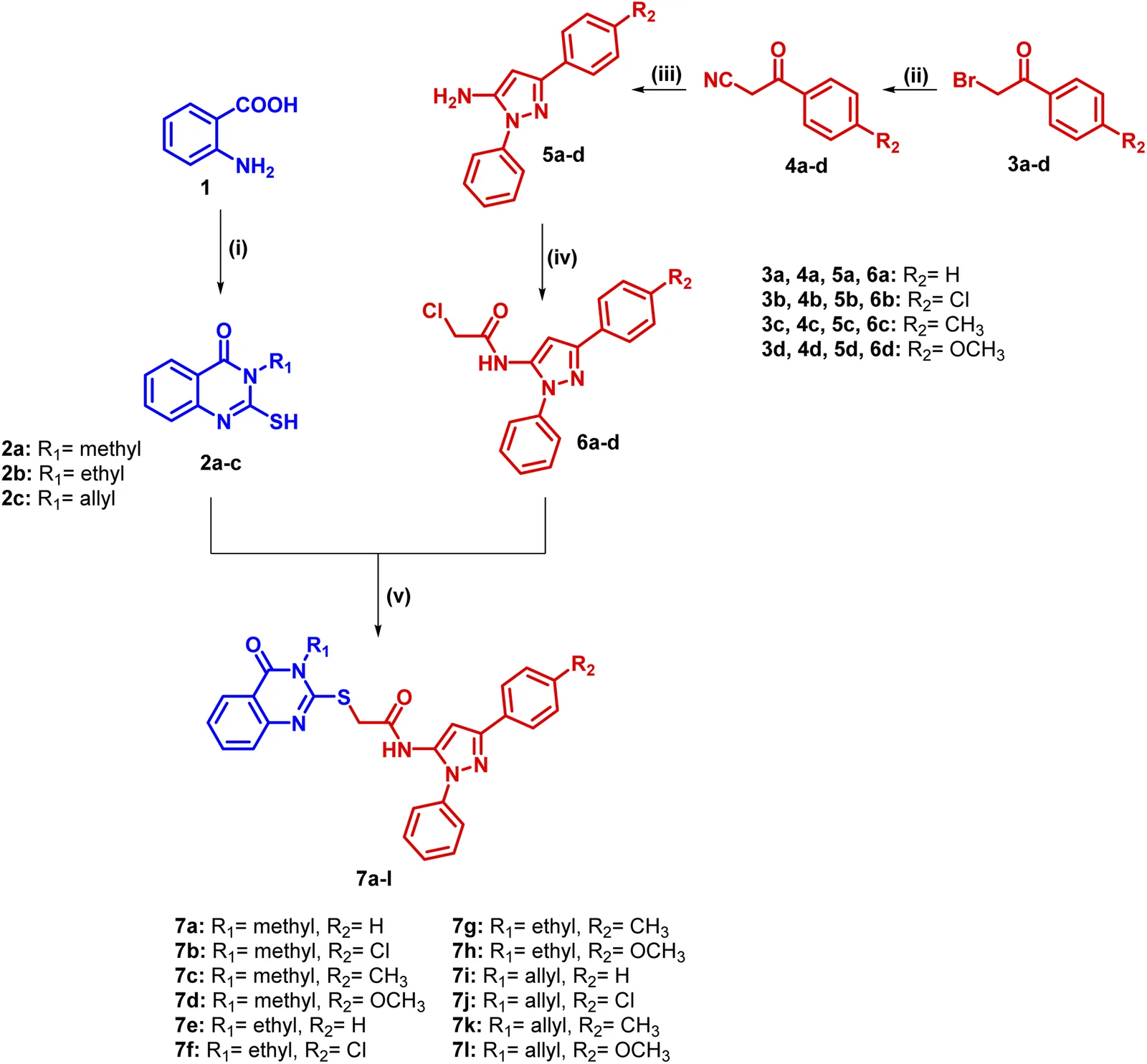
Synthesis of the target compounds 7a–l. Reagents and conditions: (i) TEA, alkyl isothiocyanate, ethanol, reflux, 2 h; (ii) KCN/ethanol, 50 °C, 2 h; (iii) phenylhydrazine/HCl, reflux, 8 h; (iv) chloroacetyl chloride, DCM, 0 °C, 12 h; (v) acetonitrile, TEA, RT, 6 h..

The structures of the newly synthesized compounds 7a–l were confirmed by ^1^H and ^13^C NMR spectroscopy, together with elemental analyses. In the ^1^H NMR spectra, the amide NH proton consistently appeared as a highly deshielded singlet at *δ* 10.35–10.40 ppm, while the linker methylene protons resonated as a two-proton singlet at *δ* 4.17–4.19 ppm. The pyrazole proton was observed as a singlet at *δ* 6.75–6.88 ppm, and the aromatic protons appeared mainly within *δ* 7.19–8.05 ppm. The N-3 substituents of the quinazolinone ring gave the expected characteristic signals: a singlet at *δ* 3.51 ppm for the *N*-methyl derivatives 7a–d, ethyl signals at *δ* 4.08–4.09 and 1.26–1.27 ppm for 7e–h, and the typical allylic pattern at *δ* 4.70, 5.12, 5.20, and 5.82–6.03 ppm for 7i–l. In addition, the *p*-methyl derivatives 7c, 7g, and 7k showed a singlet at *δ* 2.29 ppm, whereas the *p*-methoxy analogues 7d, 7h, and 7l displayed a singlet at *δ* 3.75 ppm.

The ^13^C NMR spectra further confirmed the assigned structures of 7a–l. Two characteristic carbonyl resonances were consistently observed at *δ* 167.07–167.16 and 160.75–161.13 ppm, corresponding to the amide and quinazolinone carbonyl carbons, respectively, while the pyrazole CH carbon appeared at *δ* 99.51–100.07 ppm. The S–CH_2_–CO linker gave a characteristic signal at *δ* 36.07–36.24 ppm. The different N-3 substituents were also readily distinguished: the *N*-methyl carbon of 7a–d resonated at *δ* 30.71–30.72 ppm, whereas the *N*-ethyl derivatives 7e–h showed signals at *δ* 39.54–39.61 and 13.54–13.55 ppm. The *N*-allyl derivatives 7i–l displayed characteristic resonances at *δ* 46.47–46.49 ppm for N–CH_2_ and at *δ* 131.82–131.83 and 118.23–118.25 ppm for the olefinic carbons. Furthermore, the *p*-methyl derivatives 7c, 7g, and 7k showed a signal at *δ* 21.38 ppm, while the *p*-methoxy analogues 7d, 7h, and 7l exhibited the OCH_3_ carbon at *δ* 55.62–55.67 ppm.

The ^13^C NMR data also supported the regioselectivity of the final alkylation step. Since the N-3 position of precursors 2a–c is already substituted, competing endocyclic alkylation would occur at N-1 and retain the 2-thioxo C=S functional group, whereas *S*-alkylation converts this functionality into the corresponding C2–S–CH_2_ thioether.^35^ In related quinazolinone systems, *N*-alkylated 2-thioxo derivatives retain a strongly downfield C=S resonance, while *S*-alkylated derivatives lack this signal and instead display the characteristic upfield C2 resonance together with an S–CH_2_ signal.^35,36^ Consistent with this behavior, compounds 7a–l showed no strongly downfield resonance attributable to a retained C=S group, whereas the quinazolinone C2 carbon resonated at *δ* 156.08–156.95 ppm throughout the series and the S–CH_2_–CO carbon appeared at *δ* 36.07–36.24 ppm. These spectroscopic findings, together with the elemental analyses, were consistent with the proposed S-alkylated structures of 7a–l.

### 2.2. Biological evaluation

#### 2.2.1. EGFR inhibitory activity

The newly synthesized compounds 7a–l were evaluated for their ability to inhibit EGFR, using erlotinib as the reference inhibitor. As shown in Table 1, the tested derivatives displayed EGFR inhibitory activity over a relatively narrow micromolar-to-submicromolar range, with IC_50_ values extending from 0.15 to 2.61 µM. Notably, eight of the twelve investigated compounds exhibited submicromolar EGFR inhibition, indicating that the adopted hybrid scaffold was generally compatible with EGFR inhibitory activity. Among the series, the allyl-substituted derivative 7j emerged as the most potent EGFR inhibitor, displaying an IC_50_ value of 0.15 ± 0.02 µM. This was followed by compounds 7f and 7k, which retained pronounced inhibitory activity with IC_50_ values of 0.24 ± 0.03 and 0.28 ± 0.02 µM, respectively. Although these derivatives were less potent than erlotinib (IC_50_ = 0.074 ± 0.007 µM), the activity of 7j was only approximately twofold lower than that of the reference inhibitor. Compounds 7h and 7d also demonstrated appreciable EGFR inhibition, with IC_50_ values of 0.38 ± 0.04 and 0.47 ± 0.06 µM, respectively.

**Table 1:** EGFR inhibitory activity of compounds 7a–l compared with erlotinib

Compound/reference	EGFR IC_50_ (µM) ± SEM
7a	1.64 ± 0.19
7b	0.87 ± 0.08
7c	2.61 ± 0.19
7d	0.47 ± 0.06
7e	0.66 ± 0.10
7f	0.24 ± 0.03
7g	1.45 ± 0.12
7h	0.38 ± 0.04
7i	0.69 ± 0.12
7j	0.15 ± 0.02
7k	0.28 ± 0.02
7l	1.06 ± 0.16
**Erlotinib**	0.074 ± 0.007

Examination of the structure–activity relationships indicated that EGFR inhibition was strongly influenced by the combined nature of the R_1_ and R_2_ substituents. Within the *N*-methyl series 7a–d, the methoxy derivative 7d was the most active (IC_50_ = 0.47 ± 0.06 µM), followed by the chloro analogue 7b (0.87 ± 0.08 µM), whereas the methyl-substituted compound 7c was the least active. In the *N*-ethyl series 7e–h, chlorine at R_2_ markedly favored activity, as demonstrated by 7f (0.24 ± 0.03 µM), followed by the methoxy derivative 7h (0.38 ± 0.04 µM). A similar preference for chlorine was observed in the allyl series, where 7j showed the strongest EGFR inhibition of all tested compounds (0.15 ± 0.02 µM), while the R_2_-methyl analogue 7k also retained high potency (0.28 ± 0.02 µM). Comparison of matched congeners further showed that increasing R_1_ from methyl to ethyl or allyl was particularly favorable for the chloro-substituted derivatives. Overall, the data indicate that EGFR inhibition depended on the interplay between both substitution sites, with the allyl/Cl, ethyl/Cl, and allyl/CH_3_ combinations being the most favorable.

#### 2.2.2. COX-1/COX-2 inhibitory activity

The inhibitory effects of compounds 7a–l against COX-1 and COX-2 were investigated to assess both COX-2 inhibitory potency and isoform selectivity, using celecoxib and indomethacin as reference inhibitors (Table 2). COX-2 selectivity was expressed as the selectivity index (SI), calculated as the ratio of COX-1 IC_50_ to COX-2 IC_50_. The synthesized hybrids exhibited COX-2 IC_50_ values ranging from 0.08 to 2.02 µM. Compound 7f was the most potent COX-2 inhibitor, with an IC_50_ value of 0.08 ± 0.01 µM, approaching the activities of celecoxib (0.045 ± 0.006 µM) and indomethacin (0.029 ± 0.004 µM). Compound 7j ranked second in potency (0.15 ± 0.02 µM), followed by 7e and 7k, which displayed IC_50_ values of 0.21 ± 0.03 and 0.22 ± 0.03 µM, respectively. Compounds 7c, 7l, and 7g also retained appreciable COX-2 inhibitory activity, with IC_50_ values between 0.27 and 0.37 µM.

**Table 2:** COX-1 and COX-2 inhibitory activities and COX-2 selectivity indices of compounds 7a–l compared with celecoxib and indomethacin

Compound/reference	COX-2 IC_50_ (µM) ± SEM	COX-1 IC_50_ (µM) ± SEM	COX-2 SI
7a	0.56 ± 0.09	1.09 ± 0.16	1.95
7b	2.02 ± 0.26	5.46 ± 0.87	2.70
7c	0.27 ± 0.04	0.50 ± 0.07	1.85
7d	0.97 ± 0.07	1.33 ± 0.17	1.37
7e	0.21 ± 0.03	2.81 ± 0.46	13.38
7f	0.08 ± 0.01	15.83 ± 1.77	197.88
7g	0.37 ± 0.05	1.32 ± 0.18	3.57
7h	1.27 ± 0.16	5.27 ± 0.55	4.15
7i	1.37 ± 0.24	1.45 ± 0.15	1.06
7j	0.15 ± 0.02	10.74 ± 1.23	71.60
7k	0.22 ± 0.03	3.21 ± 0.24	14.59
7l	0.32 ± 0.03	0.86 ± 0.11	2.69
**Celecoxib**	0.045 ± 0.006	14.2 ± 1.6	315.56
**Indomethacin**	0.029 ± 0.004	0.020 ± 0.003	0.69

Evaluation against COX-1 revealed marked differences in isoform selectivity. Notably, 7f exhibited weak COX-1 inhibition (IC_50_ = 15.83 ± 1.77 µM) relative to its strong COX-2 activity, resulting in the highest selectivity index among the synthesized derivatives (SI = 197.88). Although lower than that of celecoxib (SI = 315.56), this value markedly exceeded that of the nonselective inhibitor indomethacin (SI = 0.69). Compound 7j also showed pronounced COX-2 selectivity, with a COX-1 IC_50_ of 10.74 ± 1.23 µM and an SI of 71.60, while 7k and 7e displayed favorable SI values of 14.59 and 13.38, respectively.

The structure–activity relationships indicated that both COX-2 potency and selectivity were strongly influenced by the combined nature of the R_1_ and R_2_ substituents. The *N*-methyl derivatives 7a–d generally exhibited limited selectivity, with SI values ranging from 1.37 to 2.70, despite appreciable COX-2 inhibition in some cases. In contrast, introduction of an ethyl group at R_1_ markedly improved the profile of the chloro-substituted derivative 7f, which combined the strongest COX-2 inhibition with the highest isoform selectivity. A similar favorable effect of chlorine was observed in the allyl series, where 7j displayed potent COX-2 inhibition and a high SI of 71.60, while the methyl-substituted analogue 7k also retained a favorable profile. Comparison of the chloro-substituted congeners 7b, 7f, and 7j further demonstrated that chlorine at R_2_ was particularly advantageous when combined with ethyl or allyl substitution at R_1_. Overall, the biochemical data identified 7f as the most promising COX-2-directed derivative, followed by 7j, supporting the prioritization of these compounds, particularly 7f, for further cellular and mechanistic investigations.

#### 2.2.3. Antiproliferative activity

The antiproliferative activity of the synthesized compounds 7a–l was evaluated against three human cancer cell lines, namely A431 epidermoid carcinoma, A549 lung adenocarcinoma, and HCA-7 colorectal adenocarcinoma cells. Erlotinib and doxorubicin were employed as reference anticancer agents. As presented in Table 3, the investigated derivatives exhibited variable but generally pronounced antiproliferative activity, with IC_50_ values ranging from 0.74 to 8.58 µM against A431, 1.39 to 14.17 µM against A549, and 0.86 to 6.95 µM against HCA-7 cells. Analysis of the geometric mean IC_50_ values across the three cancer cell lines identified 7f, 7j, and 7k as the most broadly active members of the series, with mean IC_50_ values of 1.13, 1.18, and 1.58 µM, respectively. Notably, these values were lower than that of erlotinib (2.28 µM), although doxorubicin remained the most potent reference compound with a mean IC_50_ of 0.31 µM.

**Table 3:** Antiproliferative activity of compounds 7a–l against A431, A549, and HCA-7 cancer cell lines compared with erlotinib and doxorubicin

Compound/reference	A431 IC_50_ (µM) ± SEM	A549 IC_50_ (µM) ± SEM	HCA-7 IC_50_ (µM) ± SEM	Mean IC_50_ (µM)
7a	6.73 ± 0.69	7.76 ± 0.89	3.72 ± 0.53	5.79
7b	2.80 ± 0.34	4.91 ± 0.78	6.95 ± 1.25	4.57
7c	8.58 ± 0.83	14.17 ± 2.53	2.27 ± 0.26	6.51
7d	2.43 ± 0.45	2.82 ± 0.48	2.37 ± 0.46	2.53
7e	3.20 ± 0.55	5.17 ± 1.09	1.28 ± 0.15	2.77
7f	1.18 ± 0.14	1.42 ± 0.17	0.86 ± 0.17	1.13
7g	1.29 ± 0.28	4.48 ± 0.48	3.59 ± 0.69	2.75
7h	1.71 ± 0.19	1.93 ± 0.27	5.31 ± 0.72	2.60
7i	4.15 ± 0.75	8.61 ± 1.05	3.87 ± 0.47	5.17
7j	0.74 ± 0.08	1.39 ± 0.16	1.58 ± 0.18	1.18
7k	0.94 ± 0.16	2.14 ± 0.34	1.96 ± 0.19	1.58
7l	3.58 ± 0.52	4.07 ± 0.70	1.53 ± 0.23	2.81
**Erlotinib**	0.62 ± 0.07	2.45 ± 0.30	7.80 ± 0.95	2.28
**Doxorubicin**	0.14 ± 0.02	0.34 ± 0.04	0.61 ± 0.08	0.31

Against A431 cells, compound 7j displayed the highest activity among the synthesized derivatives, with an IC_50_ value of 0.74 ± 0.08 µM, approaching that of erlotinib (0.62 ± 0.07 µM). Compound 7k also exhibited submicromolar activity (0.94 ± 0.16 µM), whereas 7f and 7g showed pronounced effects with IC_50_ values of 1.18 ± 0.14 and 1.29 ± 0.28 µM, respectively. A somewhat different activity pattern was observed against A549 cells, where 7j and 7f were the most potent derivatives, with respective IC_50_ values of 1.39 ± 0.16 and 1.42 ± 0.17 µM. Both compounds were more active than erlotinib against this cell line (2.45 ± 0.30 µM). Compounds 7h and 7k also surpassed erlotinib, displaying IC_50_ values of 1.93 ± 0.27 and 2.14 ± 0.34 µM, respectively. Interestingly, the synthesized derivatives showed particularly favorable activity against HCA-7 cells, where all compounds exhibited lower IC_50_ values than erlotinib (7.80 ± 0.95 µM). Compound 7f was the most active derivative against HCA-7 cells, with a submicromolar IC_50_ value of 0.86 ± 0.17 µM, followed by 7e, 7l, and 7j, with IC_50_ values of 1.28 ± 0.15, 1.53 ± 0.23, and 1.58 ± 0.18 µM, respectively.

The observed antiproliferative SAR further supported the importance of the combined R_1_/R_2_ substitution pattern. In particular, chloro substitution at R_2_ was strongly favorable when associated with either ethyl or allyl substitution at R_1_, as demonstrated by the broad antiproliferative profiles of 7f and 7j. The allyl/methyl derivative 7k also retained pronounced activity across the investigated cell lines. Among these compounds, 7f showed the most balanced antiproliferative profile, being active in the low-micromolar to submicromolar range against all three cancer cell lines and displaying the lowest mean IC_50_ of the synthesized series. Taken together, these findings identify 7f, together with 7j and 7k, as the principal antiproliferative leads and are consistent with their prominent biochemical inhibitory profiles.

#### 2.2.4. Cytotoxicity toward normal human lung fibroblast MRC-5 cells

To assess whether the antiproliferative effects of the synthesized hybrids were preferentially directed toward malignant cells, compounds 7a–l were additionally evaluated against normal human lung fibroblast MRC-5 cells. Cellular selectivity indices were calculated as the ratio of the MRC-5 IC_50_ to the corresponding IC_50_ against each cancer cell line, while an overall selectivity index was obtained relative to the geometric mean IC_50_ across the three malignant cell lines. As shown in Table 4, the synthesized derivatives exhibited MRC-5 IC_50_ values ranging from 12.2 to 44.4 µM, substantially higher than their corresponding antiproliferative concentrations against the cancer cell lines in most cases. This behavior indicates a preferential cytotoxic effect toward malignant cells. In comparison, doxorubicin displayed pronounced toxicity toward MRC-5 cells (IC_50_ = 1.45 ± 0.18 µM), resulting in an overall selectivity index of only 4.72.

**Table 4:** Cytotoxicity of compounds 7a–l toward MRC-5 normal fibroblasts and their selectivity indices compared with erlotinib and doxorubicin

Compound/reference	MRC-5 IC_50_ (µM) ± SEM	SI A431	SI A549	SI HCA-7	Overall SI
7a	42.2 ± 7.6	6.27	5.44	11.34	7.29
7b	20.5 ± 2.5	7.32	4.18	2.95	4.48
7c	44.4 ± 5.3	5.17	3.13	19.56	6.82
7d	26.8 ± 3.8	11.03	9.50	11.31	10.58
7e	31.7 ± 5.4	9.91	6.13	24.77	11.46
7f	15.9 ± 3.4	13.47	11.20	18.49	14.08
7g	18.0 ± 1.9	13.95	4.02	5.01	6.55
7h	23.6 ± 3.0	13.80	12.23	4.44	9.09
7i	26.1 ± 5.3	6.29	3.03	6.74	5.05
7j	12.2 ± 2.6	16.49	8.78	7.72	10.38
7k	16.8 ± 2.8	17.87	7.85	8.57	10.63
7l	17.4 ± 1.8	4.86	4.28	11.37	6.18
**Erlotinib**	25.8 ± 3.1	41.61	10.53	3.31	11.32
**Doxorubicin**	1.45 ± 0.18	10.36	4.26	2.38	4.72

Among the investigated hybrids, 7f displayed the most favorable overall selectivity profile, with an MRC-5 IC_50_ value of 15.9 ± 3.4 µM and an overall SI of 14.08. Importantly, its selectivity was consistently maintained across all three cancer models, affording SI values of 13.47, 11.20, and 18.49 toward A431, A549, and HCA-7 cells, respectively. Thus, despite its strong antiproliferative activity, 7f exhibited substantially lower cytotoxicity toward normal fibroblasts. Its overall SI also exceeded those of both erlotinib (11.32) and doxorubicin (4.72). Compounds 7e, 7k, 7d, and 7j likewise exhibited favorable overall selectivity, with SI values of 11.46, 10.63, 10.58, and 10.38, respectively.

The cell-specific selectivity data revealed additional differences within the series. Compound 7k showed the greatest selectivity among the synthesized derivatives toward A431 cells (SI = 17.87), closely followed by 7j (SI = 16.49), whereas 7h exhibited the highest selectivity toward A549 cells (SI = 12.23). Against HCA-7 cells, 7e showed the highest selectivity index (24.77), followed by 7c (19.56) and 7f (18.49). Although individual compounds therefore displayed particularly high selectivity toward specific cancer cell types, 7f was distinguished by maintaining consistently high selectivity across the entire cancer panel rather than showing a pronounced preference for a single cell line. Collectively, these results further support 7f as the most balanced lead compound, combining broad antiproliferative potency with a favorable therapeutic window toward the tested normal cells.

#### 2.2.5. Cellular p-EGFR inhibitory activity

To confirm whether the biochemical EGFR inhibitory activity of the most promising derivatives was retained in intact A431 cells, compounds 7j, 7f, and 7k were evaluated for their ability to suppress EGFR phosphorylation, using erlotinib as the reference inhibitor. As shown in Table 5, all three compounds exhibited pronounced cellular p-EGFR inhibition, with IC_50_ values ranging from 0.59 to 0.77 µM. Compound 7j was the most potent, exhibiting an IC_50_ value of 0.59 ± 0.10 µM, followed by 7k (0.72 ± 0.13 µM) and 7f (0.77 ± 0.11 µM), while erlotinib remained more active with an IC_50_ value of 0.104 ± 0.009 µM.

**Table 5:** Cellular p-EGFR inhibitory activity and cellular shifts of compounds 7j, 7f, and 7k compared with erlotinib

Compound	Cellular p-EGFR IC_50_ (µM) ± SEM	Cellular shift^a^
7j	0.59 ± 0.10	3.93-Fold
7f	0.77 ± 0.11	3.21-Fold
7k	0.72 ± 0.13	2.57-Fold
**Erlotinib**	0.104 ± 0.009	1.41-Fold

a Cellular shift = cellular p-EGFR IC_50_/biochemical EGFR IC_50_.

The cellular shift, calculated as the ratio of the cellular p-EGFR IC_50_ to the corresponding biochemical EGFR IC_50_, was used to assess potency attenuation from the isolated enzyme assay to intact cells. A lower shift is more favorable, as it indicates better retention of biochemical potency at the cellular level. Compound 7k showed the smallest shift among the synthesized derivatives (2.57-fold), followed by 7f (3.21-fold) and 7j (3.93-fold), whereas erlotinib displayed the lowest overall shift (1.41-fold). Thus, although 7j showed the strongest absolute cellular p-EGFR inhibition, 7k exhibited the most favorable biochemical-to-cellular potency relationship among the synthesized hybrids.

Importantly, the cellular p-EGFR data provided a direct mechanistic link between EGFR target engagement and the antiproliferative response in A431 cells. The rank order of cellular p-EGFR inhibition among the investigated hybrids (7j > 7k > 7f; IC_50_ = 0.59, 0.72, and 0.77 µM, respectively) closely paralleled their antiproliferative potency in the same cell line (IC_50_ = 0.74, 0.94, and 1.18 µM, respectively). Since EGFR phosphorylation propagates proliferative and survival signaling through downstream pathways such as RAS/RAF/MEK/ERK and PI3K/AKT,^37,38^ this concordance supports a substantial EGFR-mediated component in the growth-inhibitory effects of these hybrids. This interpretation is consistent with previous multitarget EGFR-directed anticancer studies in which inhibition of EGFR signaling was accompanied by pronounced antiproliferative activity.^31,33^ Nevertheless, the imperfect correspondence between biochemical EGFR potency and cellular antiproliferative activity across the complete series indicates that additional mechanisms may contribute to the observed growth inhibition.

#### 2.2.6. Effects on cellular PGE_2_ production

To further investigate whether the observed biochemical COX-2 inhibition was translated into suppression of a downstream cellular product, compounds 7f, 7j, and 7k were evaluated for their effects on PGE_2_ production in HCA-7 cells (Table 6). The untreated control exhibited a PGE_2_ level of 5.16 ± 0.17 ng mL^−1^, while the vehicle control produced only a minor reduction to 5.02 ± 0.11 ng mL^−1^, corresponding to 2.7% inhibition. In contrast, treatment with the investigated compounds at 1.0 µM markedly reduced PGE_2_ production. Compound 7f produced the greatest effect, lowering the PGE_2_ level to 0.52 ± 0.02 ng mL^−1^, corresponding to 10.1% of the untreated control and 89.9% inhibition. Compounds 7j and 7k similarly reduced PGE_2_ levels to 0.60 ± 0.02 and 0.80 ± 0.03 ng mL^−1^, corresponding to inhibition values of 88.4% and 84.5%, respectively.

**Table 6:** Effects of compounds 7f, 7j, and 7k on cellular PGE_2_ production compared with celecoxib

Treatment	PGE_2_ level (ng mL^−1^) ± SEM	Fold change *vs.* control	Relative PGE_2_ production (% of control)	Inhibition of PGE_2_ release (%)
Untreated control	5.16 ± 0.17	1.00	100.0	0.0
Vehicle control	5.02 ± 0.11	0.97	97.3	2.7
7f	0.52 ± 0.02	0.10	10.1	89.9
7j	0.60 ± 0.02	0.12	11.6	88.4
7k	0.80 ± 0.03	0.16	15.5	84.5
**Celecoxib**	0.18 ± 0.01	0.03	3.5	96.5

The marked suppression of PGE_2_ production by 7f, 7j, and 7k demonstrates that their biochemical COX-2 inhibitory activity is retained at the cellular level. Taken together with their cellular p-EGFR inhibition and antiproliferative activity, these findings support concurrent modulation of the two intended signaling axes rather than an exclusively EGFR-dependent mechanism. This dual cellular activity is particularly relevant because both EGFR signaling and the COX-2/PGE_2_ axis contribute to cancer-cell proliferation and survival. Recent studies have similarly emphasized the importance of relating antiproliferative effects to experimentally demonstrated modulation of relevant cellular pathways rather than relying on cytotoxicity measurements alone.^39,40^

#### 2.2.7. Effect of 7f on Bax and Bcl-2 levels

To investigate whether the antiproliferative activity of the lead compound 7f was associated with modulation of apoptosis-related proteins, the levels of the pro-apoptotic protein Bax and the anti-apoptotic protein Bcl-2 were determined in HCA-7 cells, using doxorubicin as a reference drug. As summarized in Table 7, treatment with 7f markedly increased the Bax level from 0.118 ± 0.009 to 0.372 ± 0.025 pg µL^−1^, representing a 3.15-fold elevation relative to the vehicle control. In parallel, the Bcl-2 level decreased from 8.40 ± 0.70 to 3.05 ± 0.26 pg µL^−1^, corresponding to 0.36-fold of the control value. Consequently, the Bax/Bcl-2 ratio increased from 0.014 in vehicle-treated cells to 0.122 following treatment with 7f, representing an approximately 8.7-fold elevation. Doxorubicin produced a comparable pattern, increasing Bax by 3.47-fold while decreasing Bcl-2 to 0.32-fold of the control and yielding a Bax/Bcl-2 ratio of 0.155.

**Table 7:** Effects of compound 7f on Bax and Bcl-2 levels and Bax/Bcl-2 ratio compared with doxorubicin

Compound/reference	Bax (pg µL^−1^) ± SEM	Fold change	Bcl-2 (pg µL^−1^) ± SEM	Fold change	Bax/Bcl-2 ratio
Vehicle control	0.118 ± 0.009	1.00	8.40 ± 0.70	1.00	0.014
7f	0.372 ± 0.025	3.15	3.05 ± 0.26	0.36	0.122
Doxorubicin	0.410 ± 0.028	3.47	2.65 ± 0.22	0.32	0.155

The balance between pro-apoptotic Bax and anti-apoptotic Bcl-2 is an important determinant of mitochondrial susceptibility to apoptosis, and an increased Bax/Bcl-2 ratio is commonly associated with a shift toward pro-apoptotic signaling.^41,42^ Accordingly, the simultaneous elevation of Bax and suppression of Bcl-2 observed for 7f, together with the pronounced increase in their ratio, supports its ability to promote a pro-apoptotic intracellular environment. Notably, the magnitude of these changes approached that produced by doxorubicin, providing further mechanistic support for the antiproliferative effect of 7f.

#### 2.2.8. Effect of 7f on caspase-3 and caspase-9 levels

The apoptotic potential of 7f was further investigated by determining its effect on the levels of caspase-3 and caspase-9 in HCA-7 cells. The results presented in Table 8 revealed that treatment with 7f markedly elevated caspase-3 from 160 ± 20 to 1080 ± 80 pg mL^−1^, corresponding to a 6.75-fold increase relative to the vehicle control. Caspase-9 was similarly increased from 5.30 ± 0.40 to 27.6 ± 1.7 ng mL^−1^, representing a 5.21-fold elevation. Doxorubicin produced 7.00- and 7.58-fold increases in caspase-3 and caspase-9, respectively. Thus, the effect of 7f on caspase-3 closely approached that of doxorubicin, whereas its effect on caspase-9, although somewhat lower than the reference drug, remained pronounced.

**Table 8:** Effects of compound 7f on caspase-3 and caspase-9 levels compared with doxorubicin

Compound/reference	Caspase-3 (pg mL^−1^) ± SEM	Fold change	Caspase-9 (ng mL^−1^) ± SEM	Fold change
Vehicle control	160 ± 20	1.00	5.30 ± 0.40	1.00
7f	1080 ± 80	6.75	27.6 ± 1.7	5.21
Doxorubicin	1120 ± 60	7.00	40.2 ± 2.0	7.58

Caspase-9 functions upstream of the executioner caspase-3 in the mitochondrial apoptotic cascade;^43^ accordingly, coordinated increases in these caspases are commonly used as evidence supporting engagement of caspase-dependent apoptotic signaling. The concomitant elevations of caspase-9 and caspase-3 induced by 7f, together with its preceding increase in the Bax/Bcl-2 ratio, therefore provide a coherent mechanistic pattern consistent with involvement of the mitochondrial apoptotic pathway in its antiproliferative action.

When considered together, the findings obtained for 7f provide a coherent mechanistic explanation for its antiproliferative activity in HCA-7 cells. Compound 7f produced the strongest antiproliferative effect against this cell line and also caused marked suppression of cellular PGE_2_ production. This was accompanied by a pronounced shift in the Bax/Bcl-2 balance toward a pro-apoptotic state, with Bax increasing 3.15-fold, Bcl-2 decreasing to 0.36-fold of the control level, and the Bax/Bcl-2 ratio increasing approximately 8.7-fold. The subsequent elevations of caspase-9 and caspase-3 by 5.21- and 6.75-fold, respectively, further support engagement of the mitochondrial caspase-dependent apoptotic pathway.^42^ Similar mechanistic approaches employing coordinated changes in BAX, BCL-2, and caspase signaling have been used to substantiate apoptosis in recent anticancer studies.^32^ Thus, apoptosis appears to represent an important downstream component of the antiproliferative action of 7f, complementing the demonstrated modulation of EGFR phosphorylation and COX-2/PGE_2_ signaling.

### 2.3. Molecular docking

#### 2.3.1. Docking of compound 7f within the EGFR kinase domain

Molecular docking was performed to investigate the potential binding mode of 7f within the ATP-binding site of wild-type EGFR. The high-resolution crystal structure of the EGFR kinase domain co-crystallized with gefitinib (PDB ID: 4WKQ, resolution 1.85 Å) was selected for the docking study. Before docking the investigated compound, the adopted protocol was validated by re-docking the co-crystallized ligand, gefitinib, into the EGFR active site. The redocked gefitinib reproduced its experimentally observed orientation with an RMSD of 0.4225 Å and a predicted binding affinity of −8.4 kcal mol^−1^. The very low RMSD, substantially below the commonly applied 2 Å criterion for successful reproduction of crystallographic ligand poses,^44^ supports the ability of the employed docking procedure to reproduce the experimentally determined binding geometry. The superimposition of the crystallographic and redocked poses of gefitinib is shown in Fig. 4.

**Fig. 4:**
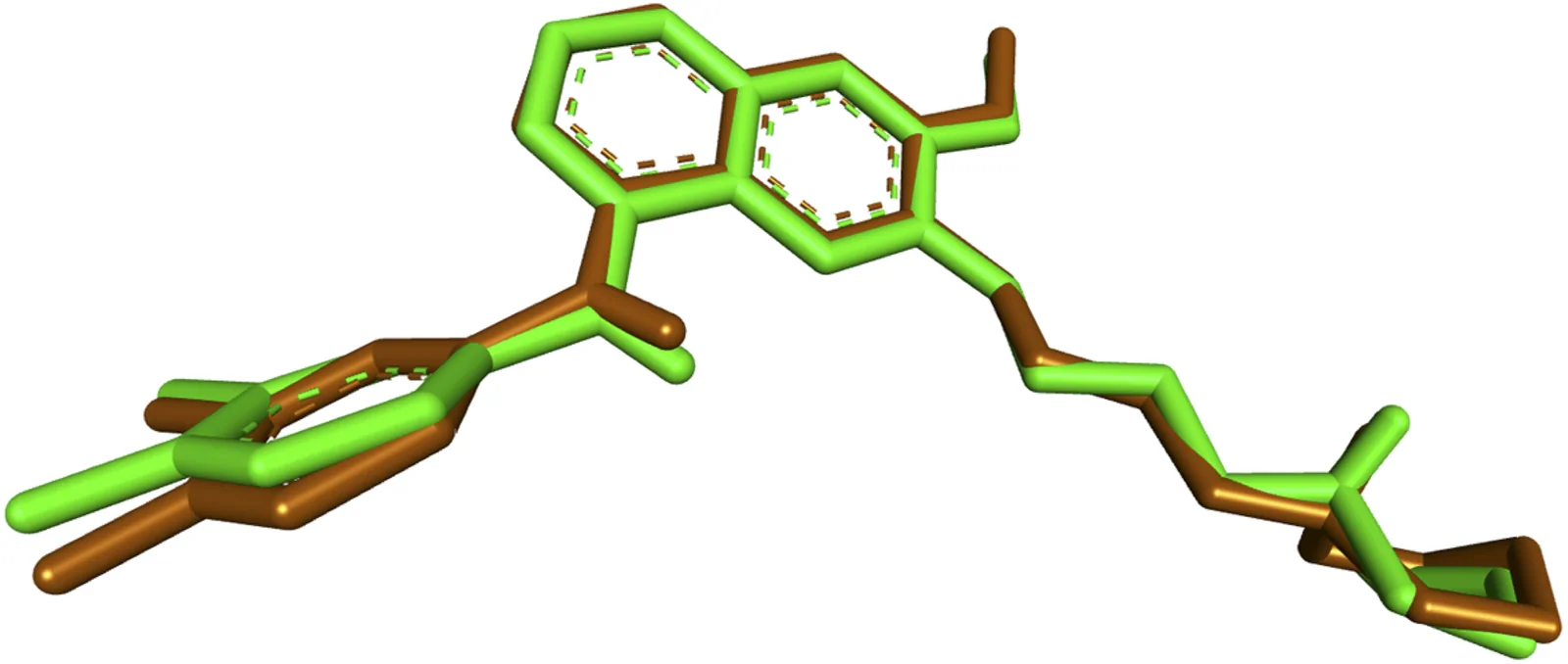
Superimposition of the co-crystallized and redocked poses of gefitinib within the ATP-binding site of wild-type EGFR (PDB ID: 4WKQ), showing an RMSD of 0.4225 Å.

Analysis of the selected docking pose of 7f revealed an extended binding mode within the EGFR ATP-binding pocket. The quinazolinone carbonyl oxygen formed a conventional hydrogen bond with Lys745, while the quinazolinone aromatic system established a π–donor hydrogen bond with Thr854 and π–alkyl interactions with Val726 and Lys745. Importantly, the thioacetamide linker carbonyl formed a conventional hydrogen bond with the key hinge residue Met793, providing an additional anchoring interaction within the ATP-binding site. The diarylpyrazole region further contributed through hydrophobic and π-mediated contacts, with the pyrazole ring forming a π–sigma interaction with Leu718, the *N*-phenyl ring establishing an amide–π stacked interaction with Leu718, and the *p*-chlorophenyl ring forming a π–alkyl interaction with Leu718 and a π–lone-pair interaction with Pro794. Collectively, these polar and hydrophobic interactions support favorable accommodation of 7f within the EGFR ATP-binding pocket and provide a plausible structural basis for its pronounced EGFR inhibitory activity. The predicted binding mode and interaction pattern of 7f are shown in Fig. 5.

**Fig. 5:**
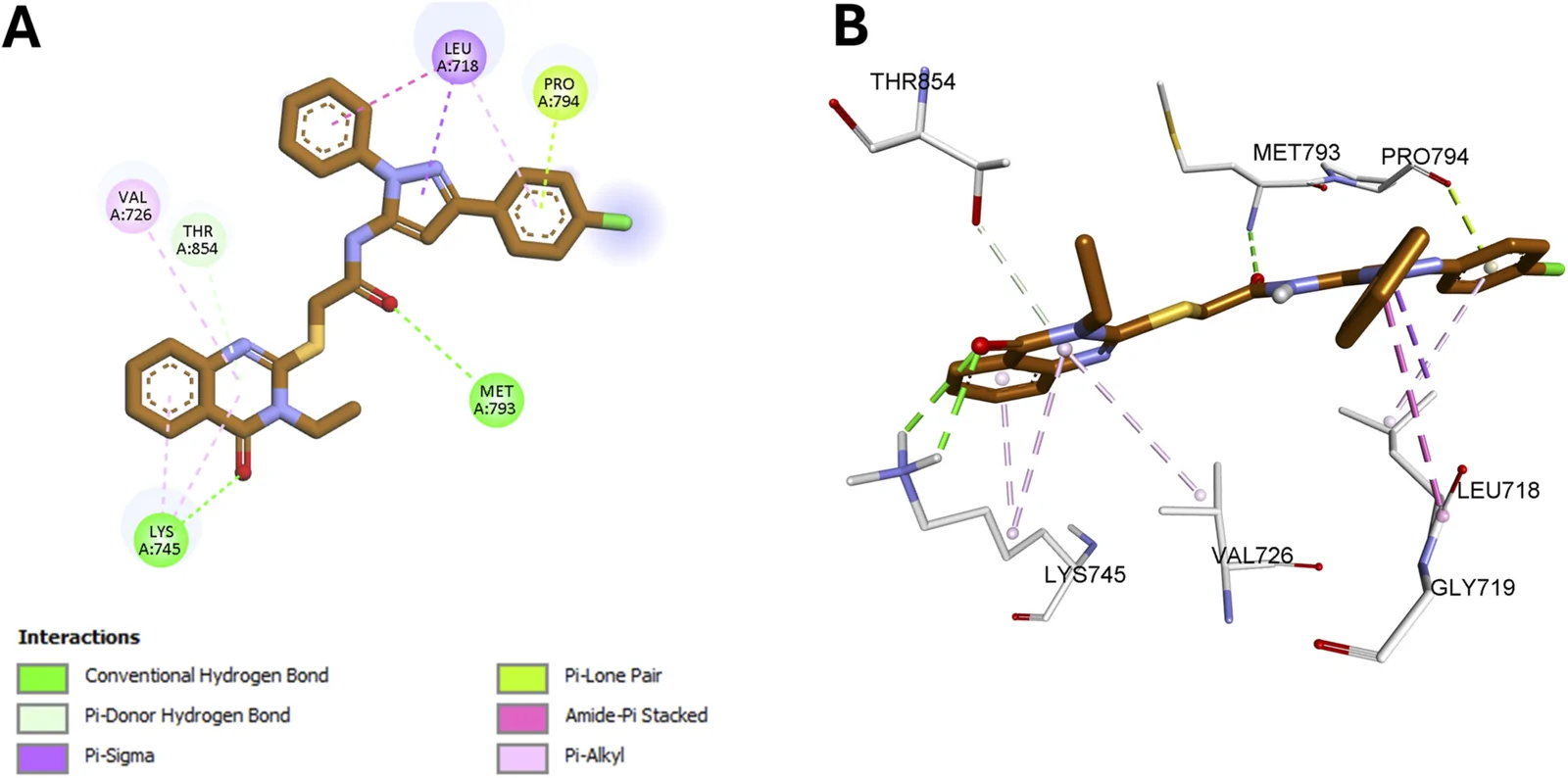
Predicted binding mode of 7f within the ATP-binding pocket of wild-type EGFR: (A) 2D interactions and (B) 3D binding pose.

#### 2.3.2. Docking of compound 7f within the COX-2 active site

Molecular docking was further performed to investigate the binding mode of 7f within the COX-2 active site. Prior to docking of the investigated compound, the docking protocol was validated by re-docking the co-crystallized ligand SC-558 into the enzyme binding pocket. The redocked SC-558 successfully reproduced its crystallographic orientation, affording an RMSD value of 0.8520 Å and a predicted binding affinity of −10.8 kcal mol^−1^. This low RMSD value further confirmed the reliability of the adopted docking protocol for reproducing the experimentally observed binding mode of the reference ligand. The superimposition of the crystallographic and redocked poses of SC-558 is shown in Fig. 6.

**Fig. 6:**
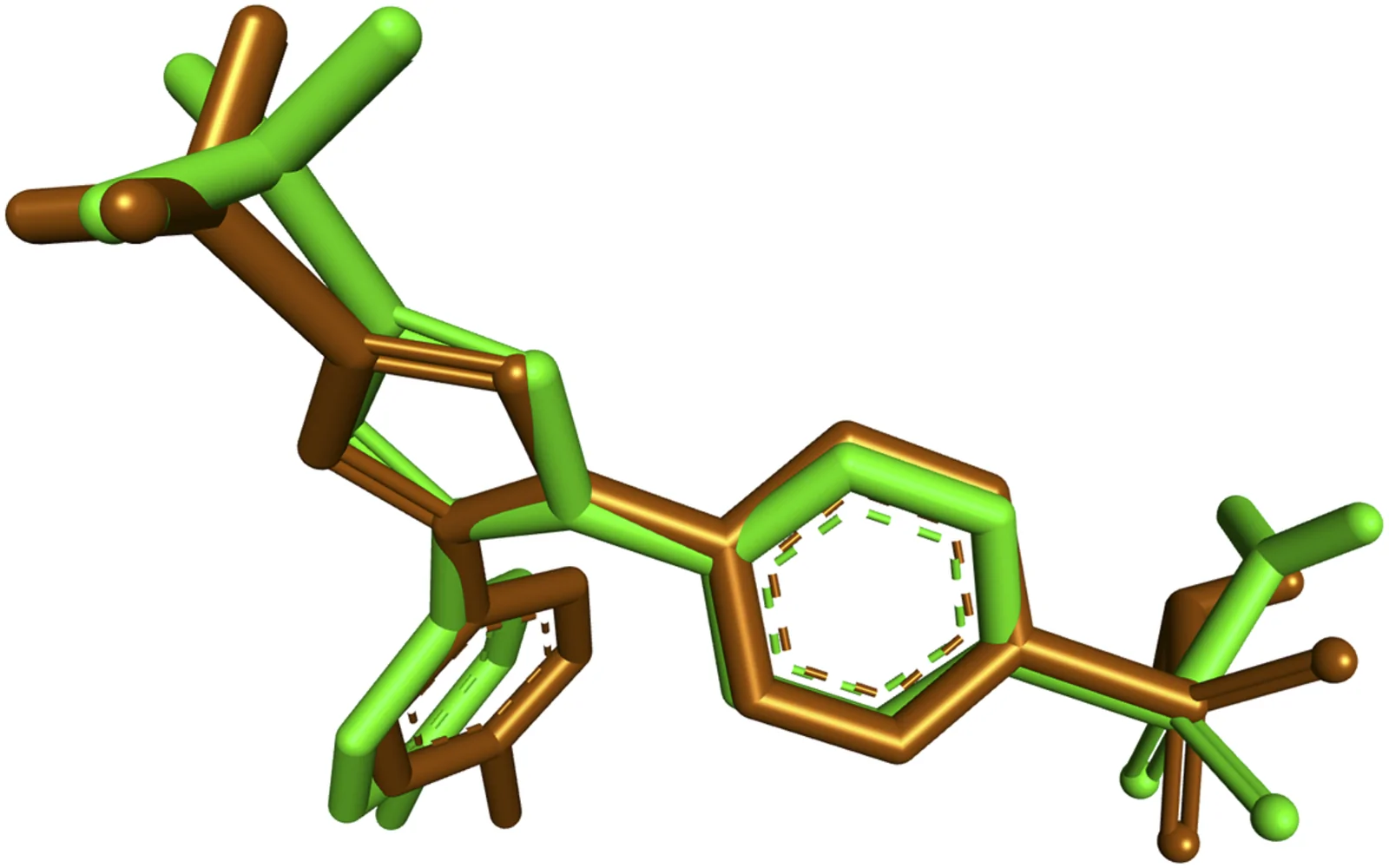
Superimposition of the co-crystallized and redocked poses of SC-558 within the COX-2 active site, showing an RMSD of 0.8520 Å.

Docking of 7f within the COX-2 binding pocket gave a predicted binding affinity of −10.5 kcal mol^−1^, indicating favorable accommodation within the active site. Analysis of the selected docking pose showed that the quinazolinone moiety played a central role in anchoring the compound, as its carbonyl oxygen formed a conventional hydrogen bond with Gln192, while its aromatic system participated in π–π stacking with His90, π–alkyl interactions with Arg513 and Pro514, a π–sigma interaction with Ala516, and an amide–π stacked interaction with Asp515. The N3-ethyl substituent further reinforced binding through carbon–hydrogen bonds with Gln192 and Leu352, together with hydrophobic contacts involving Phe518 and Ile517. The thioacetamide linker also contributed important stabilizing interactions, where the sulfur atom formed a conventional hydrogen bond with Arg513, the linker carbonyl oxygen formed a conventional hydrogen bond with His90, and the linker NH established a conventional hydrogen bond with Tyr355. In addition, the pyrazole ring contributed π–alkyl interactions with Ala527 and Val523, while its ring nitrogen formed a conventional hydrogen bond with Arg120. The terminal diaryl region also supported binding, with the *N*-phenyl ring forming a π–alkyl interaction with Val89 and a π–cation interaction with Arg120, whereas the *p*-chlorophenyl ring showed a π–alkyl interaction with Val349 and a π–sigma interaction with Ala527, and the chloro substituent itself engaged in an alkyl contact with Val349. Collectively, these multiple hydrogen-bonding, aromatic, electrostatic, and hydrophobic interactions support the stable accommodation of 7f within the COX-2 active site and provide a plausible structural basis for its potent COX-2 inhibitory activity. The predicted binding mode and interaction pattern of 7f are shown in Fig. 7.

**Fig. 7:**
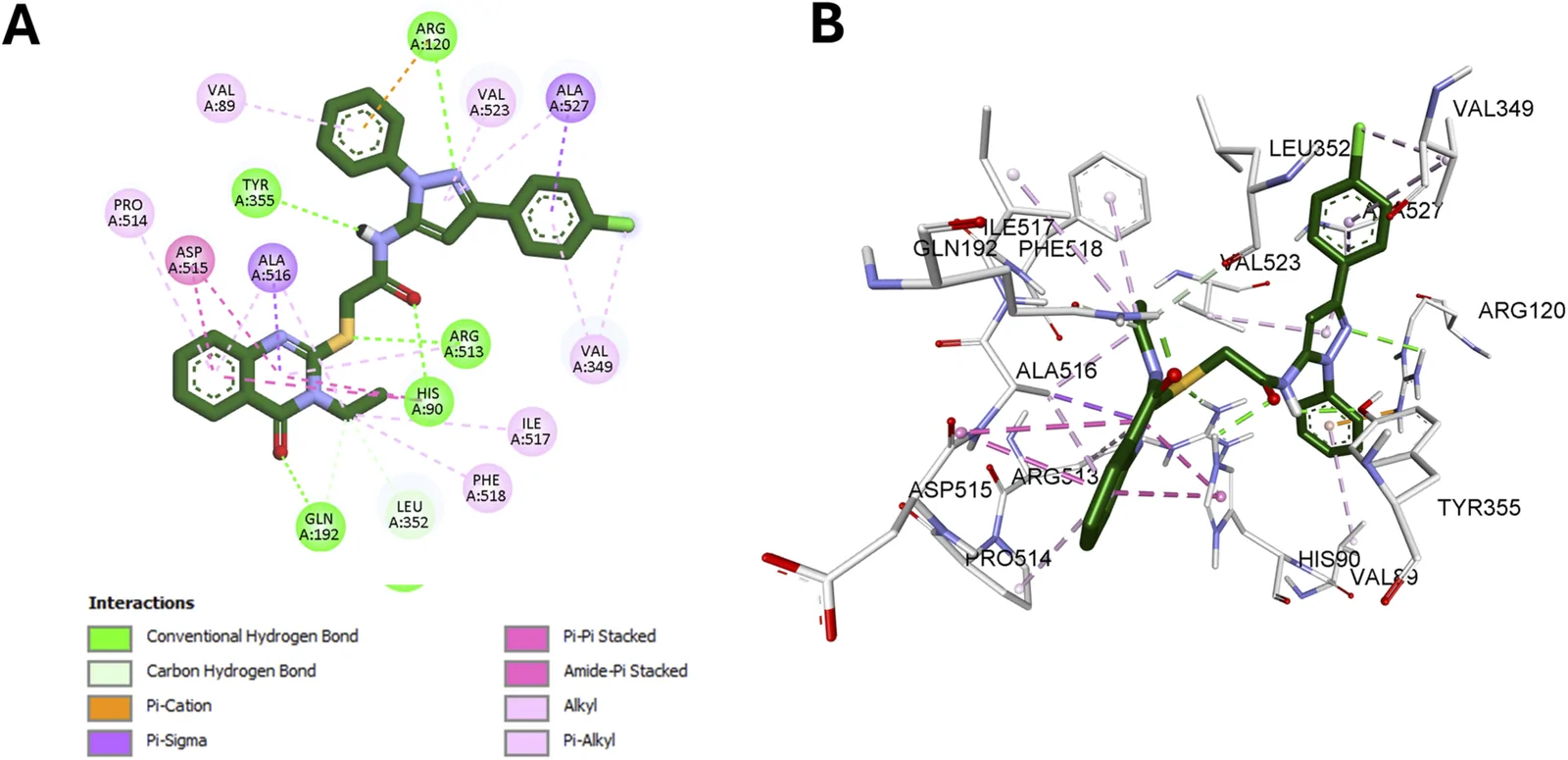
Predicted binding mode of 7f within the COX-2 active site: (A) 2D interactions and (B) 3D binding pose.

## 3. Conclusion

In this study, a series of quinazolinone–diarylpyrazole hybrids 7a–l was designed, synthesized, and biologically evaluated as dual EGFR/COX-2 inhibitors. The synthesized compounds demonstrated variable but generally promising inhibitory activity against both targets, with several derivatives also exhibiting pronounced antiproliferative effects against A431, A549, and HCA-7 cancer cells. Among the investigated compounds, 7f emerged as the most balanced lead, combining potent EGFR inhibition (IC_50_ = 0.24 ± 0.03 µM) with strong COX-2 inhibition (IC_50_ = 0.08 ± 0.01 µM) and a high COX-2 selectivity index of 197.88. Compound 7j showed the highest EGFR inhibitory activity within the series (IC_50_ = 0.15 ± 0.02 µM), further demonstrating that the developed scaffold can support inhibitory activity against both EGFR and COX-2.

The promising biochemical activity of the lead compounds was also maintained at the cellular level, as 7f, 7j, and 7k effectively inhibited EGFR phosphorylation and markedly reduced PGE_2_ production. In particular, 7f suppressed PGE_2_ release by 89.9% at 1 µM while maintaining a favorable antiproliferative and cellular selectivity profile. Mechanistic studies further demonstrated that 7f increased Bax expression, reduced Bcl-2 levels, and enhanced caspase-3 and caspase-9 levels, supporting the involvement of mitochondrial caspase-dependent apoptotic signaling in its antiproliferative activity. Molecular docking studies further supported the experimentally observed dual inhibitory activity by providing plausible binding modes of the lead compound within the EGFR and COX-2 active sites. Overall, these findings highlight 7f as the principal lead of the series and support further optimization of this quinazolinone–diarylpyrazole scaffold toward compounds displaying balanced EGFR and COX-2 inhibitory activity.

## 4. Experimental

### 4.1. Chemistry

General details: refer to SI.

Quinazolinones (2a–c),^45^ nitriles (4a–d), amino pyrazoles (5a–d) and chloroacetamides (6a–d)^21^ were prepared according to reported methods.

#### 4.1.1. General procedures for the synthesis of compounds 7a–l

The appropriate 2-mercaptoquinazolinone derivative 2a–c (1 mmol) was dissolved in 5 mL acetonitrile, followed by the addition of triethylamine (1.5 mmol, 0.152 g). The corresponding chloroacetamide derivative 6a–d (1 mmol) was then added, and the reaction mixture was stirred at room temperature for 6 h. After completion of the reaction, the mixture was poured onto ice-cold water. The formed precipitate was collected by filtration, washed thoroughly with water, and dried. The crude product was finally recrystallized from ethanol to afford the corresponding target compounds 7a–l.

##### 4.1.1.1. *N*-(1,3-Diphenyl-1*H*-pyrazol-5-yl)-2-((3-methyl-4-oxo-3,4-dihydroquinazolin-2-yl)thio)acetamide (7a)

White powder (365 mg, 78% yield), m.p: 214–216 °C; ^1^H NMR (500 MHz, DMSO-*d*_6_) *δ* (ppm): 10.37 (s, 1H, CON**H**), 8.05 (d, *J* = 7.9 Hz, 1H, **H**-5 of quinazolinone), 7.80 (d, *J* = 7.4 Hz, 2H, Ar–**H** of phenyl), 7.75–7.68 (m, 1H, **H**-7 of quinazolinone), 7.54 (d, *J* = 7.8 Hz, 2H, Ar–**H** of phenyl), 7.45–7.26 (m, 8H, **H**-6 and **H**-8 of quinazolinone & Ar–**H** of phenyl), 6.84 (s, 1H, **H**-4 of pyrazole), 4.19 (s, 2H, S–C**H**_2_), 3.51 (s, 3H, N–C**H**_3_); ^13^C NMR (125 MHz, DMSO-*d*_6_) *δ* (ppm): 167.15, 161.12, 156.92, 150.71, 147.09, 138.96, 137.55, 135.03, 133.25, 129.55, 129.24, 128.58, 128.08, 126.90, 126.41, 126.24, 125.69, 124.28, 119.10, 99.98, 36.15, 30.71. Anal. calcd. For C_26_H_21_N_5_O_2_S: C, 66.79; H, 4.53; N, 14.98. Found: C, 66.92; H, 4.42; N, 15.16.

##### 4.1.1.2. *N*-(3-(4-Chlorophenyl)-1-phenyl-1*H*-pyrazol-5-yl)-2-((3-methyl-4-oxo-3,4-dihydroquinazolin-2-yl)thio)acetamide (7b)

White powder (371 mg, 74% yield), m.p: 221–223 °C; ^1^H NMR (500 MHz, DMSO-*d*_6_) *δ* (ppm): 10.39 (s, 1H, CON**H**), 8.04 (d, *J* = 7.8 Hz, 1H, **H**-5 of quinazolinone), 7.83 (d, *J* = 8.4 Hz, 2H, Ar–**H** of phenyl), 7.72 (t, *J* = 7.1 Hz, 1H, **H**-7 of quinazolinone), 7.53 (d, *J* = 7.8 Hz, 2H, Ar–**H** of phenyl), 7.46–7.39 (m, 3H, **H**-6 of quinazolinone & Ar–**H** of phenyl), 7.38–7.27 (m, 4H, **H**-8 of quinazolinone & Ar–**H** of phenyl), 6.88 (s, 1H, **H**-4 of pyrazole), 4.19 (s, 2H, S–C**H**_2_), 3.51 (s, 3H, N–CH_3_); ^13^C NMR (125 MHz, DMSO-*d*_6_) *δ* (ppm): 167.15, 161.13, 156.92, 149.58, 147.09, 138.84, 137.73, 135.06, 133.10, 132.12, 129.57, 129.28, 128.21, 127.39, 126.90, 126.43, 126.25, 124.32, 119.09, 100.07, 36.13, 30.72. Anal. calcd. For C_26_H_20_ClN_5_O_2_S: C, 62.21; H, 4.02; N, 13.95. Found: C, 62.05; H, 4.12; N, 13.83.

##### 4.1.1.3. 2-((3-Methyl-4-oxo-3,4-dihydroquinazolin-2-yl)thio)-*N*-(1-phenyl-3-(*p*-tolyl)-1*H*-pyrazol-5-yl)acetamide (7c)

White powder (395 mg, 82% yield), m.p: 207–209 °C; ^1^H NMR (500 MHz, DMSO-*d*_6_) *δ* (ppm): 10.35 (s, 1H, CON**H**), 8.05 (d, *J* = 7.9 Hz, 1H, **H**-5 of quinazolinone), 7.76–7.65 (m, 3H, **H**-7 of quinazolinone & Ar–**H** of phenyl), 7.53 (d, *J* = 7.7 Hz, 2H, Ar–**H** of phenyl), 7.42 (t, *J* = 7.4 Hz, 1H, **H**-6 of quinazolinone), 7.37–7.32 (m, 3H, **H**-8 of quinazolinone & Ar–**H** of phenyl), 7.31–7.26 (m, 1H, Ar–**H** of phenyl), 7.20 (d, *J* = 7.5 Hz, 2H, Ar–**H** of phenyl), 6.79 (s, 1H, **H**-4 of pyrazole), 4.18 (s, 2H, S–C**H**_2_), 3.51 (s, 3H, N–C**H**_3_), 2.29 (s, 3H, C**H**_3_ of *p*-tolyl); ^13^C NMR (125 MHz, DMSO-*d*_6_) *δ* (ppm): 167.11, 161.12, 156.95, 150.75, 147.10, 139.02, 137.88, 137.47, 135.03, 130.52, 129.80, 129.52, 127.96, 126.90, 126.41, 126.25, 125.61, 124.22, 119.11, 99.81, 36.17, 30.72, 21.38. Anal. calcd. For C_27_H_23_N_5_O_2_S: C, 67.34; H, 4.81; N, 14.54. Found: C, 67.43; H, 4.67; N, 14.71.

##### 4.1.1.4. *N*-(3-(4-Methoxyphenyl)-1-phenyl-1*H*-pyrazol-5-yl)-2-((3-methyl-4-oxo-3,4-dihydroquinazolin-2-yl)thio)acetamide (7d)

White powder (378 mg, 76% yield), m.p: 218–220 °C; ^1^H NMR (500 MHz, DMSO-*d*_6_) *δ* (ppm): 10.35 (s, 1H, CON**H**), 8.05 (d, *J* = 7.8 Hz, 1H, **H**-5 of quinazolinone), 7.72 (t, *J* = 7.1 Hz, 3H, **H**-7 of quinazolinone & Ar–**H** of phenyl), 7.52 (d, *J* = 7.7 Hz, 2H, Ar–**H** of phenyl), 7.44–7.39 (m, 1H, **H**-6 of quinazolinone), 7.37–7.31 (m, 3H, **H**-8 of quinazolinone & Ar–**H** of phenyl), 7.31–7.25 (m, 1H, Ar–**H** of phenyl), 6.95 (d, *J* = 8.7 Hz, 2H, Ar–**H** of phenyl), 6.75 (s, 1H, **H**-4 of pyrazole), 4.18 (s, 2H, S–C**H**_2_), 3.75 (s, 3H, OC**H**_3_), 3.51 (s, 3H, N–C**H**_3_); ^13^C NMR (125 MHz, DMSO-*d*_6_) *δ* (ppm): 167.11, 161.12, 159.76, 156.93, 150.63, 147.09, 139.04, 137.39, 135.02, 129.52, 127.90, 127.02, 126.90, 126.39, 126.24, 125.90, 124.18, 119.10, 114.63, 99.57, 55.62, 36.17, 30.72. Anal. calcd. For C_27_H_23_N_5_O_3_S: C, 65.18; H, 4.66; N, 14.08. Found: C, 64.97; H, 4.78; N, 14.18.

##### 4.1.1.5. *N*-(1,3-Diphenyl-1*H*-pyrazol-5-yl)-2-((3-ethyl-4-oxo-3,4-dihydroquinazolin-2-yl)thio)acetamide (7e)

White powder (342 mg, 71% yield), m.p: 203–205 °C; ^1^H NMR (500 MHz, DMSO-*d*_6_) *δ* (ppm): 10.39 (s, 1H, CON**H**), 8.05 (d, *J* = 7.7 Hz, 1H, **H**-5 of quinazolinone), 7.80 (d, *J* = 7.3 Hz, 2H, Ar–**H** of phenyl), 7.76–7.69 (m, 1H, **H**-7 of quinazolinone), 7.53 (d, *J* = 7.5 Hz, 2H, Ar–**H** of phenyl), 7.46–7.36 (m, 4H) and 7.36–7.26 (m, 4H) (**H**-6 and **H**-8 of quinazolinone & Ar–**H** of phenyl), 6.84 (s, 1H, **H**-4 of pyrazole), 4.18 (s, 2H, S–C**H**_2_), 4.09 (q, *J* = 7.0 Hz, 2H, N–C**H**_2_CH_3_), 1.27 (t, *J* = 7.0 Hz, 3H, N–CH_2_C**H**_3_); ^13^C NMR (125 MHz, DMSO-*d*_6_) *δ* (ppm): 167.16, 160.77, 156.10, 150.71, 147.14, 138.99, 137.60, 135.14, 133.29, 129.54, 129.25, 128.58, 128.08, 126.88, 126.53, 126.31, 125.70, 124.31, 119.39, 99.97, 39.61, 36.09, 13.55. Anal. calcd. For C_27_H_23_N_5_O_2_S: C, 67.34; H, 4.81; N, 14.54. Found: C, 67.49; H, 4.90; N, 14.41.

##### 4.1.1.6. *N*-(3-(4-Chlorophenyl)-1-phenyl-1*H*-pyrazol-5-yl)-2-((3-ethyl-4-oxo-3,4-dihydroquinazolin-2-yl)thio)acetamide (7f)

White powder (433 mg, 84% yield), m.p: 226–228 °C; ^1^H NMR (500 MHz, DMSO-*d*_6_) *δ* (ppm): 10.40 (s, 1H, CON**H**), 8.04 (d, *J* = 7.9 Hz, 1H, **H**-5 of quinazolinone), 7.83 (d, *J* = 8.2 Hz, 2H, Ar–**H** of phenyl), 7.72 (t, *J* = 7.6 Hz, 1H, **H**-7 of quinazolinone), 7.53 (d, *J* = 7.7 Hz, 2H, Ar–**H** of phenyl), 7.46–7.39 (m, 3H, **H**-6 of quinazolinone & Ar–**H** of phenyl), 7.37–7.27 (m, 4H, **H**-8 of quinazolinone & Ar–**H** of phenyl), 6.88 (s, 1H, **H**-4 of pyrazole), 4.18 (s, 2H, S–C**H**_2_), 4.08 (q, *J* = 7.0 Hz, 2H, N–C**H**_2_CH_3_), 1.27 (t, *J* = 7.0 Hz, 3H, N–CH_2_C**H**_3_); ^13^C NMR (125 MHz, DMSO-*d*_6_) *δ* (ppm): 167.15, 160.75, 156.08, 149.60, 147.12, 138.87, 137.78, 135.13, 133.11, 132.16, 129.56, 129.28, 128.20, 127.40, 126.87, 126.52, 126.30, 124.36, 119.37, 100.03, 39.60, 36.08, 13.54. Anal. calcd. For C_27_H_22_ClN_5_O_2_S: C, 62.85; H, 4.30; N, 13.57. Found: C, 62.74; H, 4.48; N, 13.48.

##### 4.1.1.7. 2-((3-Ethyl-4-oxo-3,4-dihydroquinazolin-2-yl)thio)-*N*-(1-phenyl-3-(*p*-tolyl)-1*H*-pyrazol-5-yl)acetamide (7g)

White powder (392 mg, 79% yield), m.p: 211–214 °C; ^1^H NMR (500 MHz, DMSO-*d*_6_) *δ* (ppm): 10.37 (s, 1H, CON**H**), 8.04 (d, *J* = 7.9 Hz, 1H, **H**-5 of quinazolinone), 7.75–7.66 (m, 3H, **H**-7 of quinazolinone & Ar–**H** of phenyl), 7.52 (d, *J* = 7.8 Hz, 2H, Ar–**H** of phenyl), 7.45–7.39 (m, 1H, **H**-6 of quinazolinone), 7.36–7.31 (m, 3H, **H**-8 of quinazolinone & Ar–**H** of phenyl), 7.30–7.25 (m, 1H, Ar–**H** of phenyl), 7.19 (d, *J* = 7.7 Hz, 2H, Ar–**H** of phenyl), 6.79 (s, 1H, **H**-4 of pyrazole), 4.18 (s, 2H, S–C**H**_2_), 4.08 (q, *J* = 6.8 Hz, 2H, N–C**H**_2_CH_3_), 2.29 (s, 3H, C**H**_3_ of *p*-tolyl), 1.26 (t, *J* = 6.8 Hz, 3H, N–CH_2_C**H**_3_); ^13^C NMR (125 MHz, DMSO-*d*_6_) *δ* (ppm): 167.14, 160.76, 156.10, 150.77, 147.13, 139.02, 137.89, 137.47, 135.13, 130.54, 129.82, 129.53, 127.98, 126.87, 126.52, 126.30, 125.62, 124.26, 119.38, 99.78, 39.59, 36.09, 21.38, 13.54. Anal. calcd. For C_28_H_25_N_5_O_2_S: C, 67.86; H, 5.08; N, 14.13. Found: C, 68.08; H, 4.98; N, 14.27.

##### 4.1.1.8. 2-((3-Ethyl-4-oxo-3,4-dihydroquinazolin-2-yl)thio)-*N*-(3-(4-methoxyphenyl)-1-phenyl-1*H*-pyrazol-5-yl)acetamide (7h)

White powder (373 mg, 73% yield), m.p: 224–226 °C; ^1^H NMR (500 MHz, DMSO-*d*_6_) *δ* (ppm): 10.35 (s, 1H, CON**H**), 8.05 (d, *J* = 7.9 Hz, 1H, **H**-5 of quinazolinone), 7.73 (d, *J* = 8.3 Hz, 3H, **H**-7 of quinazolinone & Ar–**H** of phenyl), 7.52 (d, *J* = 7.7 Hz, 2H, Ar–**H** of phenyl), 7.42 (t, *J* = 7.4 Hz, 1H, **H**-6 of quinazolinone), 7.37–7.30 (m, 3H, **H**-8 of quinazolinone & Ar–**H** of phenyl), 7.30–7.25 (m, 1H, Ar–**H** of phenyl), 6.95 (d, *J* = 8.4 Hz, 2H, Ar–**H** of phenyl), 6.75 (s, 1H, **H**-4 of pyrazole), 4.18 (s, 2H, S–C**H**_2_), 4.09 (q, *J* = 6.9 Hz, 2H, N–C**H**_2_CH_3_), 3.75 (s, 3H, OC**H**_3_), 1.27 (t, *J* = 6.9 Hz, 3H, N–CH_2_C**H**_3_); ^13^C NMR (125 MHz, DMSO-*d*_6_) *δ* (ppm): 167.15, 160.76, 159.76, 156.11, 150.60, 147.13, 139.03, 137.40, 135.18, 129.53, 127.90, 127.02, 126.88, 126.55, 126.32, 125.88, 124.19, 119.35, 114.64, 99.57, 55.65, 39.54, 36.07, 13.54. Anal. calcd. For C_28_H_25_N_5_O_3_S: C, 65.74; H, 4.93; N, 13.69. Found: C, 65.61; H, 5.09; N, 13.49.

##### 4.1.1.9. 2-((3-Allyl-4-oxo-3,4-dihydroquinazolin-2-yl)thio)-*N*-(1,3-diphenyl-1*H*-pyrazol-5-yl)acetamide (7i)

White powder (400 mg, 81% yield), m.p: 198–200 °C; ^1^H NMR (500 MHz, DMSO-*d*_6_) *δ* (ppm): 10.38 (s, 1H, CON**H**), 8.05 (d, *J* = 7.9 Hz, 1H, **H**-5 of quinazolinone), 7.80 (d, *J* = 7.6 Hz, 2H, Ar–**H** of phenyl), 7.77–7.71 (m, 1H, **H**-7 of quinazolinone), 7.54 (d, *J* = 7.6 Hz, 2H, Ar–**H** of phenyl), 7.47–7.27 (m, 8H, **H**-6 and **H**-8 of quinazolinone & Ar–**H** of phenyl), 6.84 (s, 1H, **H**-4 of pyrazole), 5.99–5.82 (m, 1H, NCH_2_–C**H**=CH_2_), 5.20 (d, *J* = 10.4 Hz, 1H, =C**H**_2_), 5.12 (d, *J* = 17.5 Hz, 1H, =C**H**_2_), 4.70 (d, *J* = 3.2 Hz, 2H, N–C**H**_2_–CH=CH_2_), 4.17 (s, 2H, S–C**H**_2_); ^13^C NMR (125 MHz, DMSO-*d*_6_) *δ* (ppm): 167.13, 160.79, 156.53, 150.71, 147.15, 138.99, 137.58, 135.30, 133.27, 131.82, 129.58, 129.26, 128.59, 128.09, 127.01, 126.63, 126.37, 125.70, 124.31, 119.26, 118.25, 99.96, 46.49, 36.24. Anal. calcd. For C_28_H_23_N_5_O_2_S: C, 68.14; H, 4.70; N, 14.19. Found: C, 68.24; H, 4.51; N, 14.31.

##### 4.1.1.10. 2-((3-Allyl-4-oxo-3,4-dihydroquinazolin-2-yl)thio)-*N*-(3-(4-chlorophenyl)-1-phenyl-1*H*-pyrazol-5-yl)acetamide (7j)

White powder (407 mg, 77% yield), m.p: 232–234 °C; ^1^H NMR (500 MHz, DMSO-*d*_6_) *δ* (ppm): 10.40 (s, 1H, CON**H**), 8.05 (d, *J* = 7.9 Hz, 1H, **H**-5 of quinazolinone), 7.83 (d, *J* = 8.5 Hz, 2H, Ar–**H** of phenyl), 7.74 (t, *J* = 7.1 Hz, 1H, **H**-7 of quinazolinone), 7.53 (d, *J* = 7.7 Hz, 2H, Ar–**H** of phenyl), 7.43 (t, *J* = 9.4 Hz, 3H, **H**-6 of quinazolinone & Ar–**H** of phenyl), 7.35 (t, *J* = 7.3 Hz, 3H, **H**-8 of quinazolinone & Ar–**H** of phenyl), 7.32–7.28 (m, 1H, Ar–**H** of phenyl), 6.88 (s, 1H, **H**-4 of pyrazole), 5.97–5.85 (m, 1H, NCH_2_–C**H**=CH_2_), 5.20 (d, *J* = 10.4 Hz, 1H, =C**H**_2_), 5.12 (d, *J* = 17.3 Hz, 1H, =C**H**_2_), 4.70 (d, *J* = 4.8 Hz, 2H, N–C**H**_2_–CH=CH_2_), 4.17 (s, 2H, S–C**H**_2_); ^13^C NMR (125 MHz, DMSO-*d*_6_) *δ* (ppm): 167.12, 160.78, 156.51, 149.59, 147.14, 138.87, 137.76, 135.31, 133.11, 132.14, 131.83, 129.60, 129.30, 128.22, 127.41, 127.01, 126.63, 126.38, 124.36, 119.25, 118.24, 100.03, 46.48, 36.22. Anal. calcd. For C_28_H_22_ClN_5_O_2_S: C, 63.69; H, 4.20; N, 13.26. Found: C, 63.51; H, 4.31; N, 13.35.

##### 4.1.1.11. 2-((3-Allyl-4-oxo-3,4-dihydroquinazolin-2-yl)thio)-*N*-(1-phenyl-3-(*p*-tolyl)-1*H*-pyrazol-5-yl)acetamide (7k)

White powder (381 mg, 75% yield), m.p: 205–207 °C; ^1^H NMR (500 MHz, DMSO-*d*_6_) *δ* (ppm): 10.36 (s, 1H, CON**H**), 8.05 (d, *J* = 7.9 Hz, 1H, **H**-5 of quinazolinone), 7.76–7.72 (m, 1H, **H**-7 of quinazolinone), 7.69 (d, *J* = 8.0 Hz, 2H, Ar–**H** of phenyl), 7.53 (d, *J* = 7.8 Hz, 2H, Ar–**H** of phenyl), 7.46–7.39 (m, 1H, **H**-6 of quinazolinone), 7.35 (t, *J* = 7.9 Hz, 3H, **H**-8 of quinazolinone & Ar–**H** of phenyl), 7.31–7.25 (m, 1H, Ar–**H** of phenyl), 7.19 (d, *J* = 8.0 Hz, 2H, Ar–**H** of phenyl), 6.79 (s, 1H, **H**-4 of pyrazole), 6.03–5.82 (m, 1H, NCH_2_–C**H**=CH_2_), 5.20 (d, *J* = 10.4 Hz, 1H, =C**H**_2_), 5.12 (d, *J* = 17.2 Hz, 1H, =C**H**_2_), 4.70 (d, *J* = 4.8 Hz, 2H, N–C**H**_2_–CH=CH_2_), 4.17 (s, 2H, S–C**H**_2_), 2.29 (s, 3H, C**H**_3_ of *p*-tolyl); ^13^C NMR (125 MHz, DMSO-*d*_6_) *δ* (ppm): 167.10, 160.79, 156.53, 150.77, 147.15, 139.03, 137.90, 137.46, 135.28, 131.83, 130.54, 129.82, 129.55, 127.99, 127.01, 126.62, 126.37, 125.63, 124.26, 119.27, 118.23, 99.77, 46.49, 36.24, 21.38. Anal. calcd. For C_29_H_25_N_5_O_2_S: C, 68.62; H, 4.96; N, 13.80. Found: C, 68.76; H, 4.84; N, 13.59.

##### 4.1.1.12. 2-((3-Allyl-4-oxo-3,4-dihydroquinazolin-2-yl)thio)-*N*-(3-(4-methoxyphenyl)-1-phenyl-1*H*-pyrazol-5-yl)acetamide (7l)

White powder (435 mg, 83% yield), m.p: 219–221 °C; ^1^H NMR (500 MHz, DMSO-*d*_6_) *δ* (ppm): 10.35 (s, 1H, CON**H**), 8.05 (d, *J* = 7.9 Hz, 1H, **H**-5 of quinazolinone), 7.74 (t, *J* = 9.3 Hz, 3H, **H**-7 of quinazolinone & Ar–**H** of phenyl), 7.52 (d, *J* = 7.6 Hz, 2H, Ar–**H** of phenyl), 7.46–7.40 (m, 1H, **H**-6 of quinazolinone), 7.34 (t, *J* = 9.0 Hz, 3H, **H**-8 of quinazolinone & Ar–**H** of phenyl), 7.31–7.24 (m, 1H, Ar–**H** of phenyl), 6.95 (d, *J* = 8.7 Hz, 2H, Ar–**H** of phenyl), 6.76 (s, 1H, **H**-4 of pyrazole), 5.98–5.83 (m, 1H, NCH_2_–C**H**=CH_2_), 5.20 (d, *J* = 10.4 Hz, 1H, =C**H**_2_), 5.12 (d, *J* = 17.5 Hz, 1H, =C**H**_2_), 4.70 (d, *J* = 4.6 Hz, 2H, N–C**H**_2_–CH=CH_2_), 4.17 (s, 2H, S–C**H**_2_), 3.75 (s, 3H, OC**H**_3_); ^13^C NMR (125 MHz, DMSO-*d*_6_) *δ* (ppm): 167.07, 160.77, 159.76, 156.52, 150.62, 147.13, 139.04, 137.39, 135.28, 131.82, 129.56, 129.51, 127.90, 127.02, 126.61, 126.36, 125.90, 124.20, 119.25, 118.24, 114.64, 99.51, 55.67, 46.47, 36.24. Anal. calcd. For C_29_H_25_N_5_O_3_S: C, 66.52; H, 4.81; N, 13.38. Found: C, 66.43; H, 5.01; N, 13.51.

### 4.2. Biology

#### 4.2.1. EGFR inhibition assay

The EGFR inhibitory activities of compounds 7a–l were determined using the ADP-Glo™ Kinase Assay + EGFR Kinase Enzyme System (Promega Corporation, Madison, WI, USA; Cat. No. V9261), with erlotinib as the reference inhibitor, following the manufacturer's recommended assay procedure. Additional information regarding the assay conditions and experimental protocol is included in the SI.

#### 4.2.2. COX inhibition assay

The inhibitory activities of compounds 7a–l against COX-1 and COX-2 were determined using the COX (ovine/human) Inhibitor Screening Assay Kit (Cayman Chemical, Ann Arbor, MI, USA; Item No. 560131), with celecoxib and indomethacin as reference inhibitors, following the manufacturer's recommended assay procedure. A detailed description of the experimental procedure is presented in the SI.

#### 4.2.3. Antiproliferative activity

The antiproliferative activities of compounds 7a–l against A431, A549, HCA-7, and MRC-5 cells were evaluated using the MTT-based *in vitro* Toxicology Assay Kit (Sigma-Aldrich, St. Louis, MO, USA; TOX1),^46^ with erlotinib and doxorubicin as reference drugs, following the manufacturer's recommended procedure. Further details concerning the MTT assay protocol are provided in the SI.

#### 4.2.4. Cellular p-EGFR inhibition assay

The cellular p-EGFR inhibitory activities of compounds 7j, 7f, and 7k were evaluated in A431 cells using the Human EGFR (pY1068) ELISA Kit (Abcam, Cambridge, UK; Cat. No. ab126438), with erlotinib as the reference inhibitor, following the manufacturer's recommended procedure. The complete experimental protocol for the cellular p-EGFR determination is described in the SI.

#### 4.2.5. Effects on cellular PGE_2_ production

The effects of compounds 7f, 7j, and 7k on cellular PGE_2_ production were evaluated using the Parameter™ Prostaglandin E_2_ Immunoassay (R&D Systems, Minneapolis, MN, USA; Cat. No. KGE004B), with celecoxib as the reference inhibitor, following the manufacturer's recommended procedure. Details of the cell treatment and PGE_2_ quantification procedures can be found in the SI.

#### 4.2.6. Effect on Bax and Bcl-2 levels

The effects of compound 7f on Bax and Bcl-2 levels were determined using the DRG Human Bax ELISA Kit (DRG International, Inc., USA; Cat. No. EIA-4487) and Zymed Bcl-2 ELISA Kit (Invitrogen, Carlsbad, CA, USA; Cat. No. 99-0042), with doxorubicin as the reference drug, following the manufacturers' recommended procedures. The experimental conditions employed for Bax and Bcl-2 quantification are outlined in the SI.

#### 4.2.7. Effect on caspase-3 and caspase-9 levels

The effects of compound 7f on active caspase-3 and caspase-9 levels were determined using the Human caspase-3 (Active) ELISA Kit (Invitrogen, Thermo Fisher Scientific, USA; Cat. No. KHO1091) and Human caspase-9 ELISA Kit (Thermo Fisher Scientific, USA; Cat. No. BMS2025), respectively, with doxorubicin as the reference drug, following the manufacturers' recommended procedures. Full experimental details are provided in the SI.

### 4.3. Molecular docking

Molecular docking of 7f against EGFR (PDB ID: 4WKQ) and COX-2 (PDB ID: 1CX2) was performed using AutoDock Vina.^47^ Protein and ligand preparation was carried out using AutoDockTools,^48^ and the docking protocols were validated by re-docking the respective co-crystallized ligands. Docked complexes were analyzed and visualized using BIOVIA Discovery Studio Visualizer. Full details of the docking protocol and parameters are provided in the SI.

## Author contributions

Abdullah Alkhammash: conceptualization, investigation, formal analysis, writing – original draft. Basima A. A. Saleem: methodology, investigation, validation, data curation. Khulood H. Oudah: methodology, investigation, data curation, writing – review & editing. Mohamed Ahmed: methodology, formal analysis, validation, writing – review & editing. Kasim Sakran Abass: methodology, investigation, formal analysis, data curation. Sara Mahmoud Farhan: methodology, investigation, formal analysis, validation. Magdi E. A. Zaki: software, formal analysis, visualization, validation. Sobhi M. Gomha: conceptualization, supervision, project administration, writing – review & editing.

## Conflicts of interest

The authors declare no conflicts of interest.

## Supplementary Material

RA-016-D6RA07867B-s001

## Data Availability

The original contributions presented in this study are included in the article; further inquiries can be directed to the corresponding author. Supplementary information (SI) is available. See DOI: https://doi.org/10.1039/d6ra07867b.

## References

[cit1] Hanahan D. (2022). Hallmarks of Cancer: New Dimensions. Cancer Discovery.

[cit2] Maddeboina K., Yada B., Kumari S., McHale C., Pal D., Durden D. L. (2024). Recent advances in multitarget-directed ligands via in silico drug discovery. Drug Discov. Today.

[cit3] Kabir A., Muth A. (2022). Polypharmacology: The science of multi-targeting molecules. Pharmacol. Res..

[cit4] Finetti F., Paradisi L., Bernardi C., Pannini M., Trabalzini L. (2023). Cooperation between Prostaglandin E2 and Epidermal Growth Factor Receptor in Cancer Progression: A Dual Target for Cancer Therapy. Cancers.

[cit5] Zhang Y. (2023). Targeting Epidermal Growth Factor Receptor for Cancer Treatment: Abolishing Both Kinase-Dependent and Kinase-Independent Functions of the Receptor. Pharmacol. Rev..

[cit6] Gao C., Wang W., Liu T., Li X., Yu Y., Wu J. (2025). Annual review of EGFR inhibitors in 2024. Eur. J. Med. Chem..

[cit7] Ghoneim M. M., Abdelgawad M. A., Elkanzi N. A. A., Parambi D. G. T., Alsalahat I., Farouk A., Bakr R. B. (2024). A literature review on pharmacological aspects, docking studies, and synthetic approaches of quinazoline and quinazolinone derivatives. Arch. Pharm. (Weinheim, Ger.).

[cit8] Sandor A., Ionut I., Marc G., Oniga I., Eniu D., Oniga O. (2023). Structure-Activity Relationship Studies Based on Quinazoline Derivatives as EGFR Kinase Inhibitors (2017-Present). Pharmaceuticals.

[cit9] El-Mahdy R. Y., Galal N., Lotfy R., Arafa R. K. (2025). Structure-based design of new anticancer N3-Substituted quinazolin-4-ones as type I ATP-competitive inhibitors targeting the deep hydrophobic pocket of EGFR. Comput. Biol. Med..

[cit10] Hamdi A., Tawfik S. S., Ali A. R., Ewes W. A., Haikal A., El-Azab A. S., Bakheit A. H., Hefnawy M. M., Ghabbour H. A., Abdel-Aziz A. A.-M. (2024). Harnessing potential COX-2 engagement for boosting anticancer activity of substituted 2-mercapto-4(3*H*)-quinazolinones with promising EGFR/VEGFR-2 inhibitory activities. Bioorg. Chem..

[cit11] Ahmadi M., Bekeschus S., Weltmann K.-D., von Woedtke T., Wende K. (2022). Non-steroidal anti-inflammatory drugs: recent advances in the use of synthetic COX-2 inhibitors. RSC Med. Chem..

[cit12] Chun K.-S., Kim E.-H., Kim D.-H., Song N.-Y., Kim W., Na H.-K., Surh Y.-J. (2024). Targeting cyclooxygenase-2 for chemoprevention of inflammation-associated intestinal carcinogenesis: An update. Biochem. Pharmacol..

[cit13] Finetti F., Travelli C., Ercoli J., Colombo G., Buoso E., Trabalzini L. (2020). Prostaglandin E2 and Cancer: Insight into Tumor Progression and Immunity. Biology.

[cit14] Zhang Y. , Tighe S. and Zhu Y.-T., COX-2 Signaling in the Tumor Microenvironment, in Tumor Microenvironment : Molecular Players – Part B, ed. A. Birbrair, Springer International Publishing, Cham, 2020, pp. 87–104, 10.1007/978-3-030-50224-9_6 33119867

[cit15] Santiso A., Heinemann A., Kargl J. (2024). Prostaglandin E2 in the Tumor Microenvironment, a Convoluted Affair Mediated by EP Receptors 2 and 4. Pharmacol. Rev..

[cit16] Kassab A. E. (2025). Recent advances in targeting COX-2 for cancer therapy: a review. RSC Med. Chem..

[cit17] Khalil N. A., Ahmed E. M., Tharwat T., Mahmoud Z. (2024). NSAIDs between past and present; a long journey towards an ideal COX-2 inhibitor lead. RSC Adv..

[cit18] Kumar R., Sharma R., Kumar Sharma D. (2023). Pyrazole; A Privileged Scaffold of Medicinal Chemistry: A Comprehensive Review. Curr. Top. Med. Chem..

[cit19] Ghoneim M. M., Abdelgawad M. A., Elkanzi N. A. A., Bakr R. B. (2025). Review of the recent advances of pyrazole derivatives as selective COX-2 inhibitors for treating inflammation. Mol. Diversity.

[cit20] Li G., Cheng Y., Han C., Song C., Huang N., Du Y. (2022). Pyrazole-containing pharmaceuticals: target, pharmacological activity, and their SAR studies. RSC Med. Chem..

[cit21] Farag K. O., Nour M. S., Abdelhafez M. M., Ibrahim N. M., Abdel-Aziz H. A., Fahim S. H. (2026). Pyrazole derivatives as dual COX-2/EGFR inhibitors with anticancer potential: Optimization of difenamizole Analogs. Bioorg. Chem..

[cit22] Tveteraas I. H., Müller K. M., Aasrum M., Ødegård J., Dajani O., Guren T., Sandnes D., Christoffersen T. (2012). Mechanisms involved in PGE2-induced transactivation of the epidermal growth factor receptor in MH1C1 hepatocarcinoma cells. J. Exp. Clin. Cancer Res..

[cit23] Wang D., Xia D., DuBois R. N. (2011). The Crosstalk of PTGS2 and EGF Signaling Pathways in Colorectal Cancer. Cancers.

[cit24] Hu H., Han T., Zhuo M., Wu L., Yuan C., Wu L., Lei W., Jiao F., Wang L.-W. (2017). Elevated COX-2 Expression Promotes Angiogenesis Through EGFR/p38-MAPK/Sp1-Dependent Signalling in Pancreatic Cancer. Sci. Rep..

[cit25] Valverde A., Peñarando J., Cañas A., López-Sánchez L. M., Conde F., Hernández V., Peralbo E., López-Pedrera C., de la Haba-Rodríguez J., Aranda E., Rodríguez-Ariza A. (2015). Simultaneous Inhibition of EGFR/VEGFR and Cyclooxygenase-2 Targets Stemness-Related Pathways in Colorectal Cancer Cells. PLoS One.

[cit26] Ahmed E. A., Mohamed M. F. A., Omran O. A. (2022). Novel quinoxaline derivatives as dual EGFR and COX-2 inhibitors: synthesis, molecular docking and biological evaluation as potential anticancer and anti-inflammatory agents. RSC Adv..

[cit27] Kothayer H., Rezq S., Abdelkhalek A. S., Romero D. G., Elbaramawi S. S. (2023). Triple targeting of mutant EGFRL858R/T790M, COX-2, and 15-LOX: design and synthesis of novel quinazolinone tethered phenyl urea derivatives for anti-inflammatory and anticancer evaluation. J. Enzyme Inhib. Med. Chem..

[cit28] Kahk N. M., Mohamed F. E. A., Abdelhakeem M. M., Bakr R. B. (2025). Optimization of pyrazole/1,2,4-triazole as dual EGFR/COX-2 inhibitors: Design, synthesis, anticancer potential, apoptosis induction and cell cycle analysis. Eur. J. Med. Chem..

[cit29] Soltan O. M., Abdel-Aziz S. A., Abdelrahman K. S., Narumi A., Abdel-Aziz M., Shoman M. E., Konno H. (2026). Design, synthesis, and biological evaluation of novel 1,5-diarylpyrazole carboxamides with dual inhibition of EGFR and COX-2 for the treatment of cancer and inflammatory diseases. Bioorg. Med. Chem. Lett..

[cit30] Serag M. I., Hamdi A., Nasr E. E., Elnagar M. R., Tawfik M. M., El-Azab A. S., Brogi S., Al-Suwaidan I. A., Hefnawy M., Abdel-Aziz A. A.-M. (2025). Exploiting COX-2 engagement for amplifying anticancer potential of Quinazoline-triazole conjugates with superior EGFR/VEGFR-2 inhibition. Bioorg. Chem..

[cit31] Gaber A. A., Abo Elmaaty A., Sharaky M., Mosa A. A., Yahya Abdullah Alzahrani A., Shaaban S., Eldehna W. M., Al-Karmalawy A. A. (2024). Multi-target rational design and synthesis of novel diphenyl-tethered pyrazolopyrimidines targeting EGFR and topoisomerase II with potential DNA intercalation and apoptosis induction. Bioorg. Chem..

[cit32] Shaaban S., Althikrallah H. A., Negm A., Abo Elmaaty A., Al-Karmalawy A. A. (2024). Repurposed organoselenium tethered amidic acids as apoptosis inducers in melanoma cancer via P53, BAX, caspases-3, 6, 8, 9, BCL-2, MMP2, and MMP9 modulations. RSC Adv..

[cit33] Al-Karmalawy A. A., Elmaaty A. A., Alabdali A. Y. M., Shaaban S., Khatib A. O. A., Abdellattif M. H., Alnjaa A. A., Sharaky M., Eldehna W. M., Gaber A. A. (2025). Exploring the anticancer potential of new pyrazolopyrimidine analogues as multi-target directed EGFR/STAT3 downregulatory candidates with apoptotic potential. New J. Chem..

[cit34] Hawash M., Jaradat N., Abualhasan M., Şüküroğlu M. K., Qaoud M. T., Kahraman D. C., Daraghmeh H., Maslamani L., Sawafta M., Ratrout A., Issa L. (2023). Design, synthesis, molecular docking studies and biological evaluation of thiazole carboxamide derivatives as COX inhibitors. BMC Chem..

[cit35] Ghorab W. M., El-Sebaey S. A., Ghorab M. M. (2023). Design, synthesis and molecular modeling study of certain quinazolinone derivatives targeting poly (ADP-ribose) polymerase 1 (PARP-1) enzyme as anti-breast cancer and radio-sensitizers. J. Mol. Struct..

[cit36] Abuelizz H. A., Marzouk M., Ghabbour H., Al-Salahi R. (2017). Synthesis and anticancer activity of new quinazoline derivatives. Saudi Pharm. J..

[cit37] Halder S., Basu S., Lall S. P., Ganti A. K., Batra S. K., Seshacharyulu P. (2023). Targeting the EGFR signaling pathway in cancer therapy: What's new in 2023?. Expert Opin. Ther. Targets.

[cit38] Li Y., Mao T., Wang J., Zheng H., Hu Z., Cao P., Yang S., Zhu L., Guo S., Zhao X., Tian Y., Shen H., Lin F. (2023). Toward the next generation EGFR inhibitors: an overview of osimertinib resistance mediated by EGFR mutations in non-small cell lung cancer. Cell Commun. Signaling.

[cit39] Althikrallah H. A., Shaaban S., Elmaaty A. A., Ba-Ghazal H., Almarri M. N., Sharaky M., Alnajjar R., Al-Karmalawy A. A. (2024). Investigating the anti-inflammatory potential of N-amidic acid organoselenium candidates: biological assessments, molecular docking, and molecular dynamics simulations. RSC Adv..

[cit40] Shaaban S., Yousef T. A., Althikrallah H. A., Al-Faiyz Y. S., Elghamry I., Sharaky M., Alnajjar R., Al-Karmalawy A. A. (2025). Unveiling the anti-inflammatory potential of organoselenium Schiff bases: computational and in vitro studies. New J. Chem..

[cit41] Kale J., Osterlund E. J., Andrews D. W. (2018). BCL-2 family proteins: changing partners in the dance towards death. Cell Death Differ..

[cit42] Pan Y., Ye C., Tian Q., Yan S., Zeng X., Xiao C., Wang L., Wang H. (2018). miR-145 suppresses the proliferation, invasion and migration of NSCLC cells by regulating the BAX/BCL-2 ratio and the caspase-3 cascade. Oncol. Lett..

[cit43] Boice A., Bouchier-Hayes L. (2020). Targeting apoptotic caspases in cancer. Biochim. Biophys. Acta – Mol. Cell Res..

[cit44] Ramírez D., Caballero J. (2018). Is It Reliable to Take the Molecular Docking Top Scoring Position as the Best Solution without Considering Available Structural Data?. Molecules.

[cit45] Khodair A. I., Alsafi M. A., Nafie M. S. (2019). Synthesis, molecular modeling and anti-cancer evaluation of a series of quinazoline derivatives. Carbohydr. Res..

[cit46] van Meerloo J. , Kaspers G. J. L. and Cloos J., Cell Sensitivity Assays: The MTT Assay, in Cancer Cell Culture: Methods and Protocols, ed. I. A. Cree, Humana Press, Totowa, NJ, 2011, pp. 237–245, 10.1007/978-1-61779-080-5_20 21516412

[cit47] Trott O., Olson A. J. (2010). AutoDock Vina: Improving the speed and accuracy of docking with a new scoring function, efficient optimization, and multithreading. J. Comput. Chem..

[cit48] Morris G. M., Huey R., Lindstrom W., Sanner M. F., Belew R. K., Goodsell D. S., Olson A. J. (2009). AutoDock4 and AutoDockTools4: Automated docking with selective receptor flexibility. J. Comput. Chem..

