# Supplementary material for: Quinazolinone–diarylpyrazole hybrids as dual EGFR/COX-2 inhibitors: antiproliferative, mechanistic, and in silico studies
Source: RSC Adv. 2026 Sep 24. Online ahead of print. doi: 10.1039/d6ra07867b (PMC13603062; doi:10.1039/d6ra07867b)
Supplement: Supplementary file 1 [file RA-OLF-D6RA07867B-s001.pdf]

Supplementary Information (SI) for RSC Advances.
This journal is © The Royal Society of Chemistry 2026
Supplementary Information (SI) for RSC Advances.
This journal is © The Royal Society of Chemistry

Quinazoline–Diarylpyrazole Hybrids as Dual EGFR/COX-2 Inhibitors: Antiproliferative, Mechanistic, and In Silico Studies

Abdullah Alkhammash[1], Basima A. A. Saleem[2], Khulood H. Oudah[3], Mohamed Ahmed[4], Kasim Sakran Abass[5,*], Sara Mahmoud Farhan[6], Magdi E. A. Zaki[7], Sobhi M. Gomha[8,*]

[1]Department of Pharmacology, College of Pharmacy, Al-Dawadmi Campus, Shaqra University, Shaqra 11961, Saudi Arabia

[2]Department of Chemistry, College of Science, University of Mosul, Mosul 41001, Iraq

[3]Department of Pharmacy, Mazaya University College, Nasiriyah, Iraq

[4]College of Medicine, Dhofar University, Salalah 122, Oman

[5]Department of Physiology, Biochemistry and Pharmacology, College of Veterinary Medicine, University of Kirkuk, Kirkuk 36001, Iraq

[6]Department of Microbiology and Immunology, Faculty of Pharmacy, Deraya University, New Minia 61768, Egypt

[7]Department of Chemistry, Faculty of Science, Imam Mohammad Ibn Saud Islamic University (IMSIU), Riyadh 11623, Saudi Arabia

[8]Department of Chemistry, Faculty of Science, Islamic University of Madinah, Madinah, 42351, Saudi Arabia

1. Chemistry

1.1. General details

All reagents and solvents were of general purpose or analytical grade and purchased from Sigma Aldrich Ltd, Fisher Scientific, Fluka and Acros. ^{1}H- and ^{13}C-NMR spectra were recorded with a Bruker Avance III spectrometer operating at 500, 120 MHz respectively, with Me_4Si as internal standard and DMSO-d_6 as a solvent. Elemental analysis was performed by the regional center for mycology and biotechnology (Cairo, Egypt). TLC was carried out on precoated silica plates (Keisel gel 60 F254, BDH). Compounds were visualized by illumination under UV light (254 nm). Melting points were determined on an electrothermal instrument and are uncorrected. All solvents were dried prior to use and stored over 4 Å molecular sieves, under nitrogen.

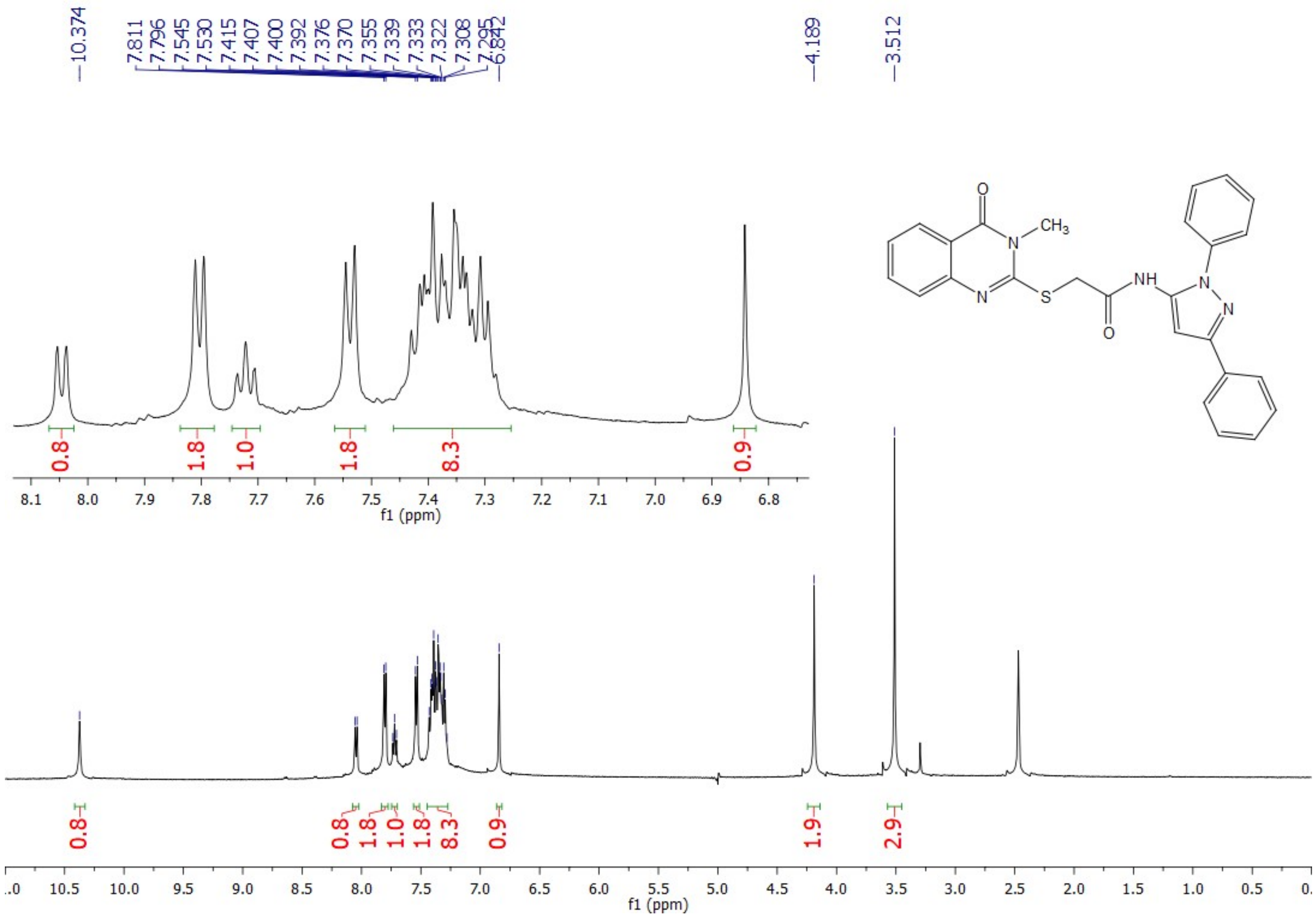

Figure S1. ^{1}H NMR spectrum of compound **7a**

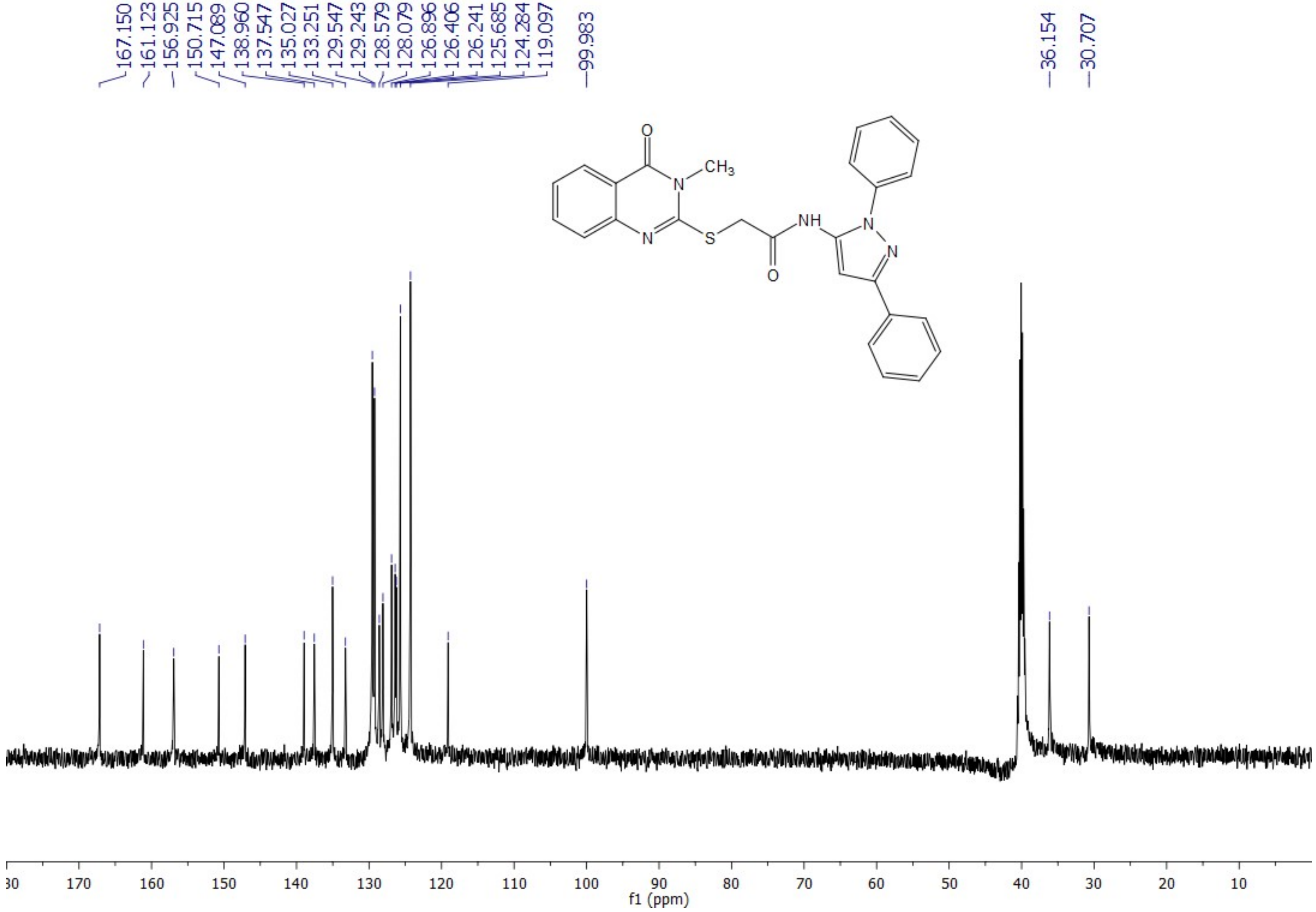

Figure S2. ^{13}C NMR spectrum of compound **7a**

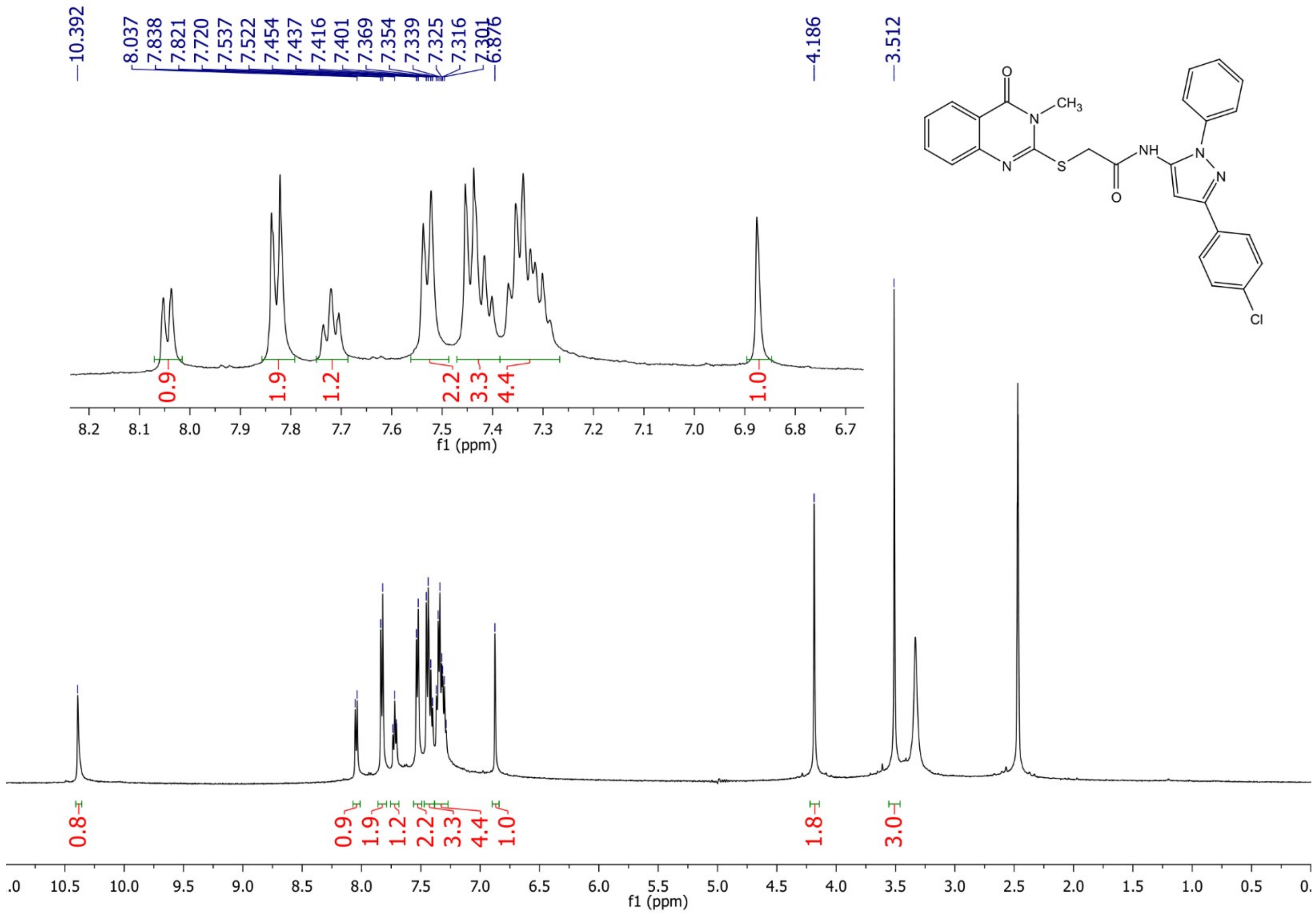

Figure S3. ^{1}H NMR spectrum of compound **7b**

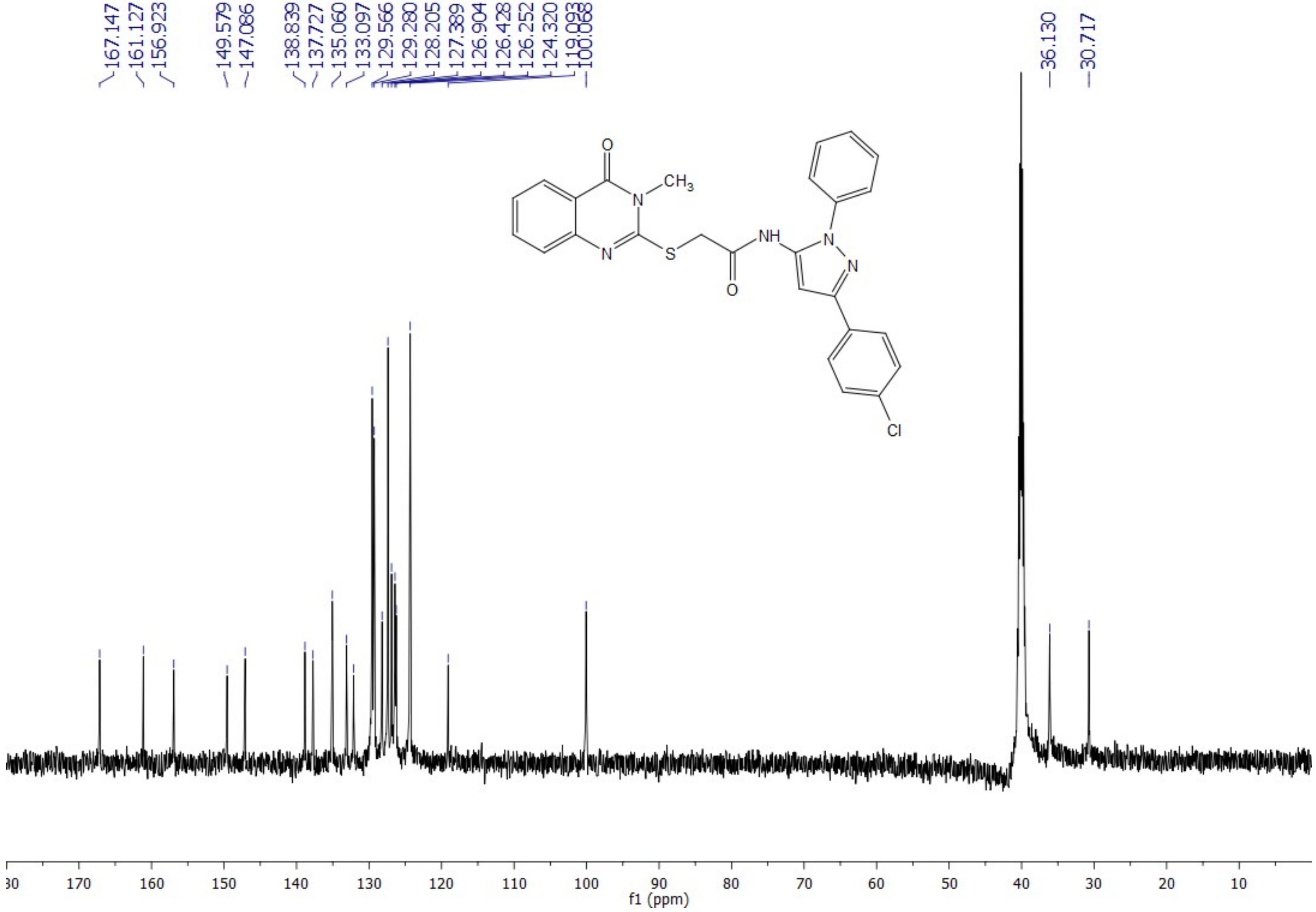

Figure S4. ^{13}C NMR spectrum of compound **7b**

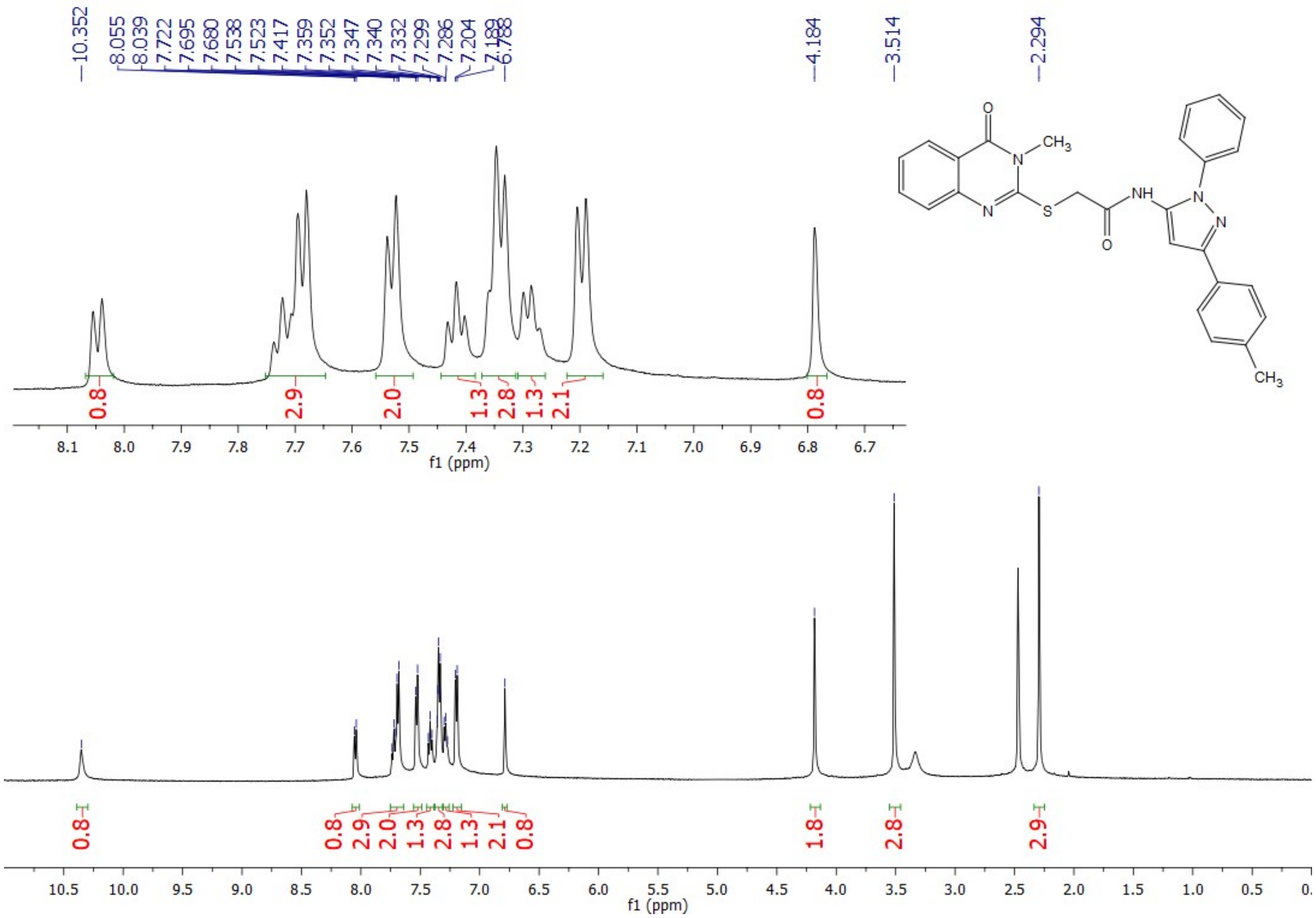

Figure S5. ^{1}H NMR spectrum of compound **7c**

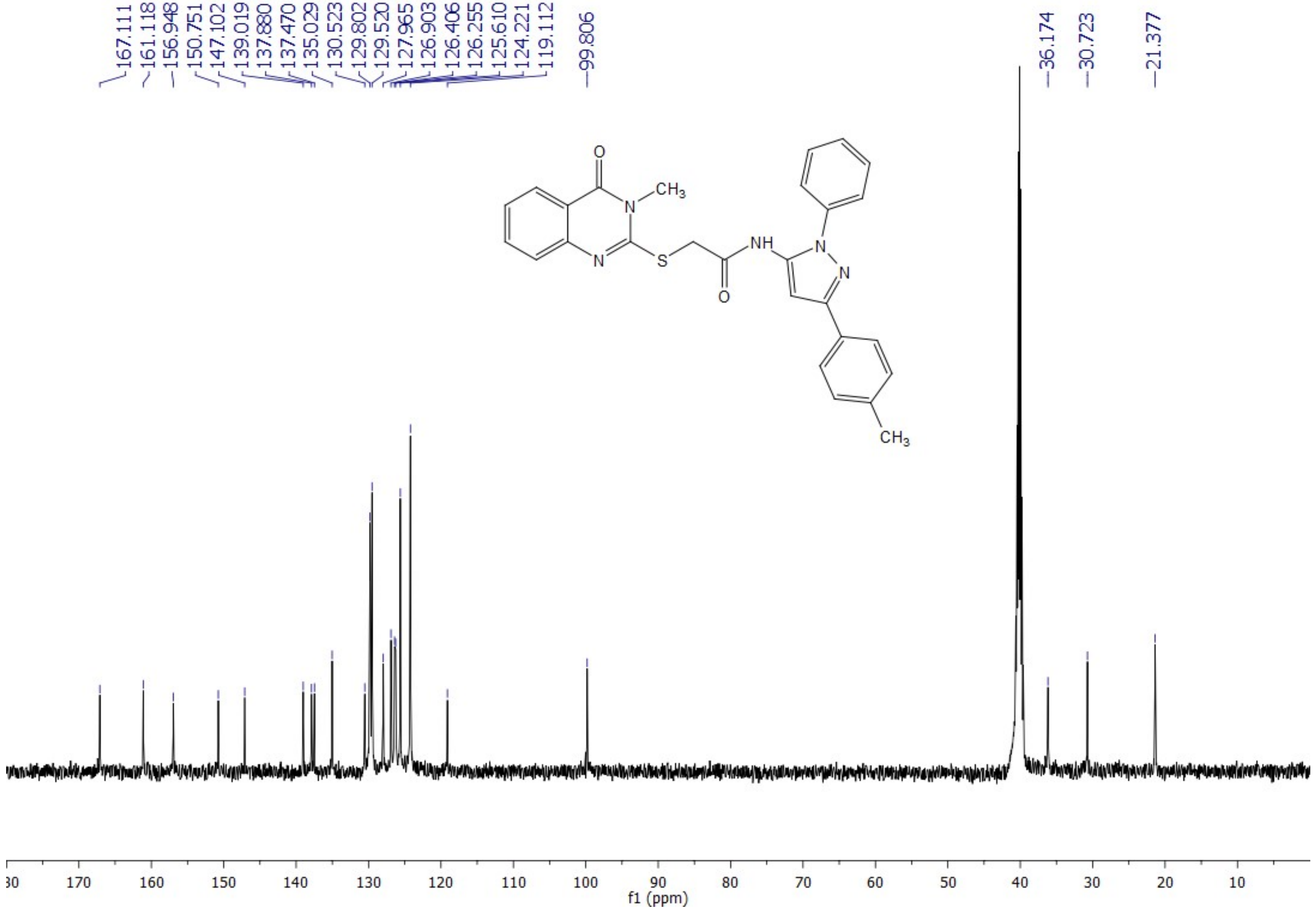

Figure S6. ^{13}C NMR spectrum of compound **7c**

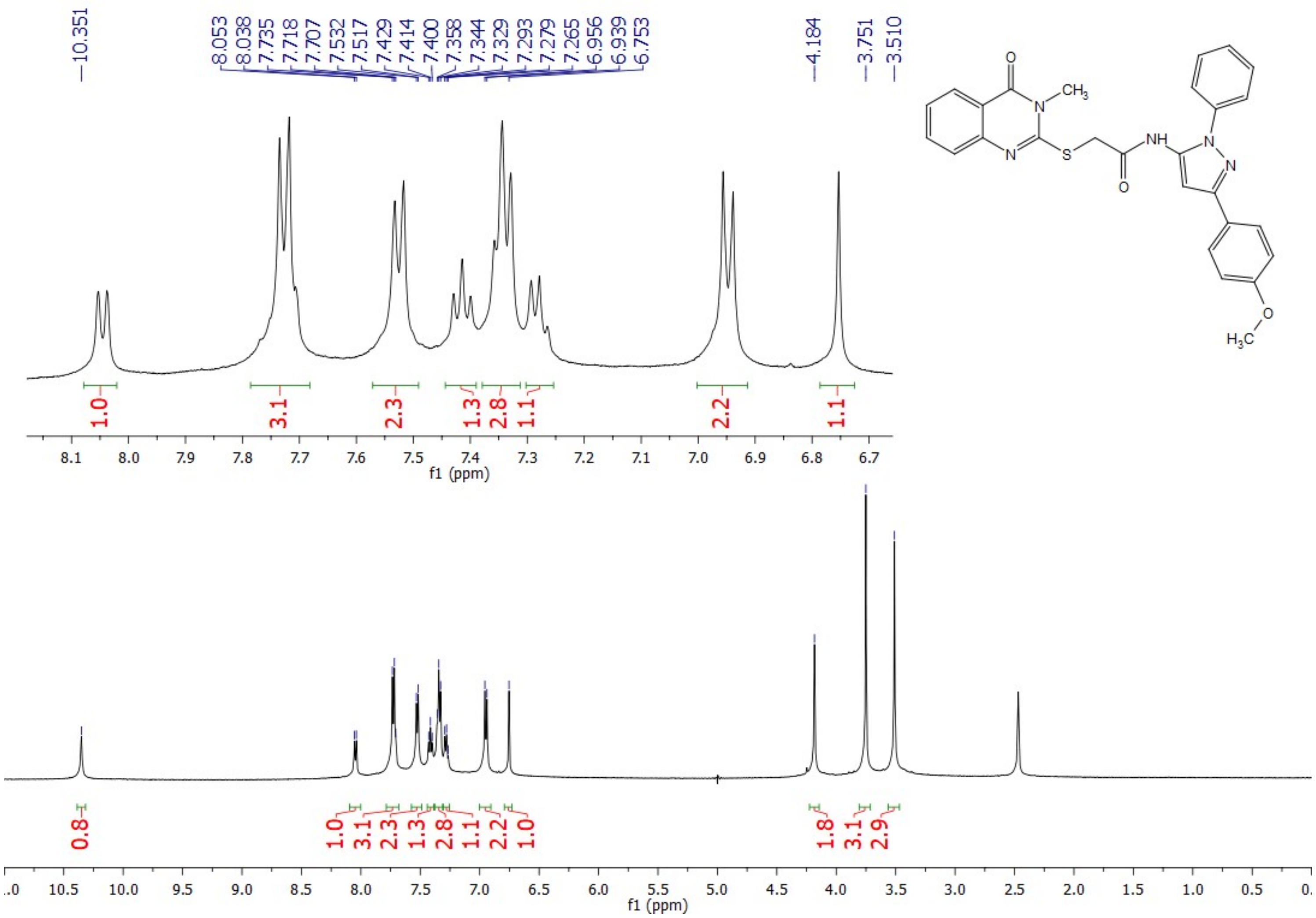

Figure S7. ^{1}H NMR spectrum of compound **7d**

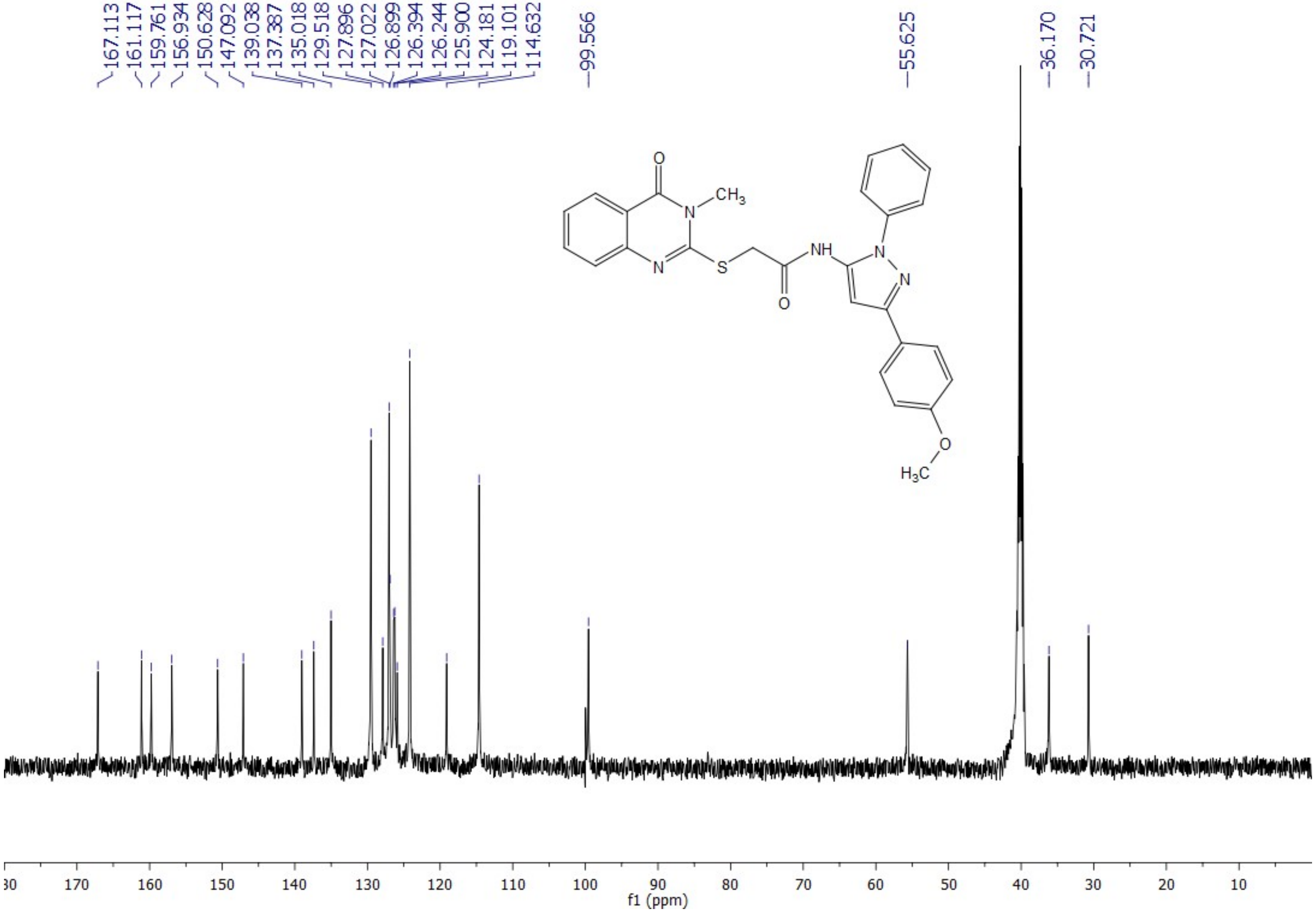

Figure S8. ^{13}C NMR spectrum of compound **7d**

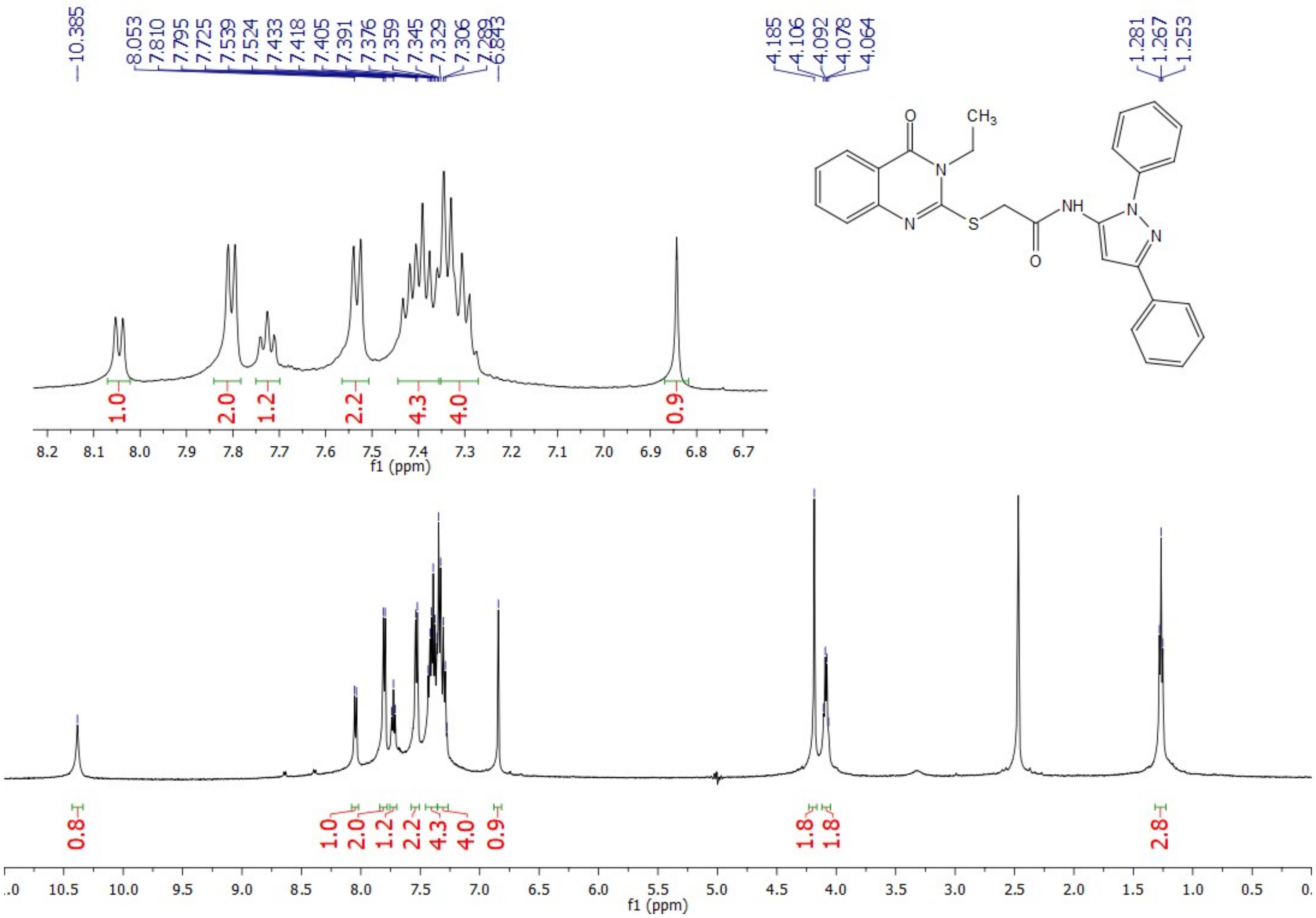

Figure S9. ^{1}H NMR spectrum of compound **7e**

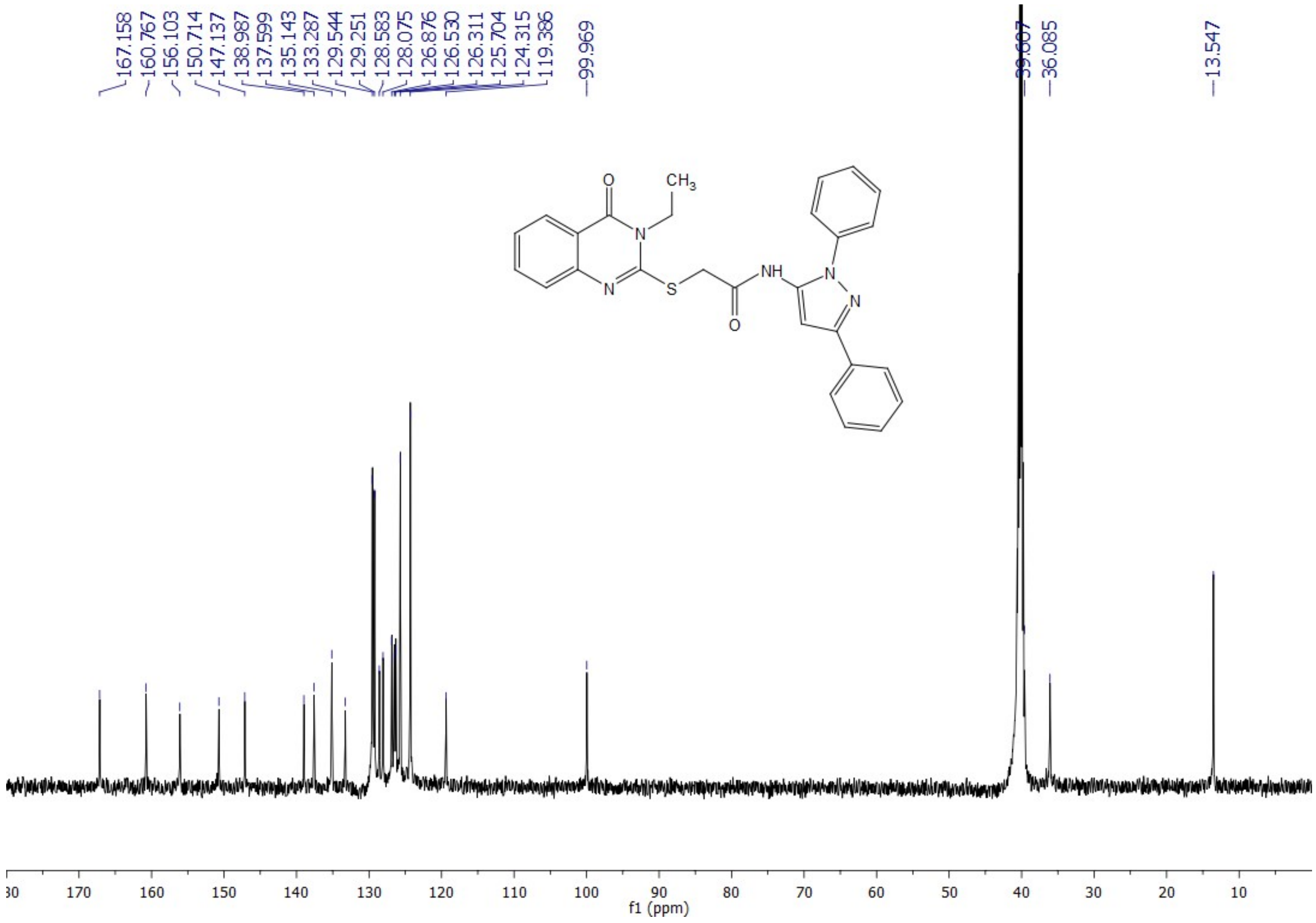

Figure S10. ^{13}C NMR spectrum of compound **7e**

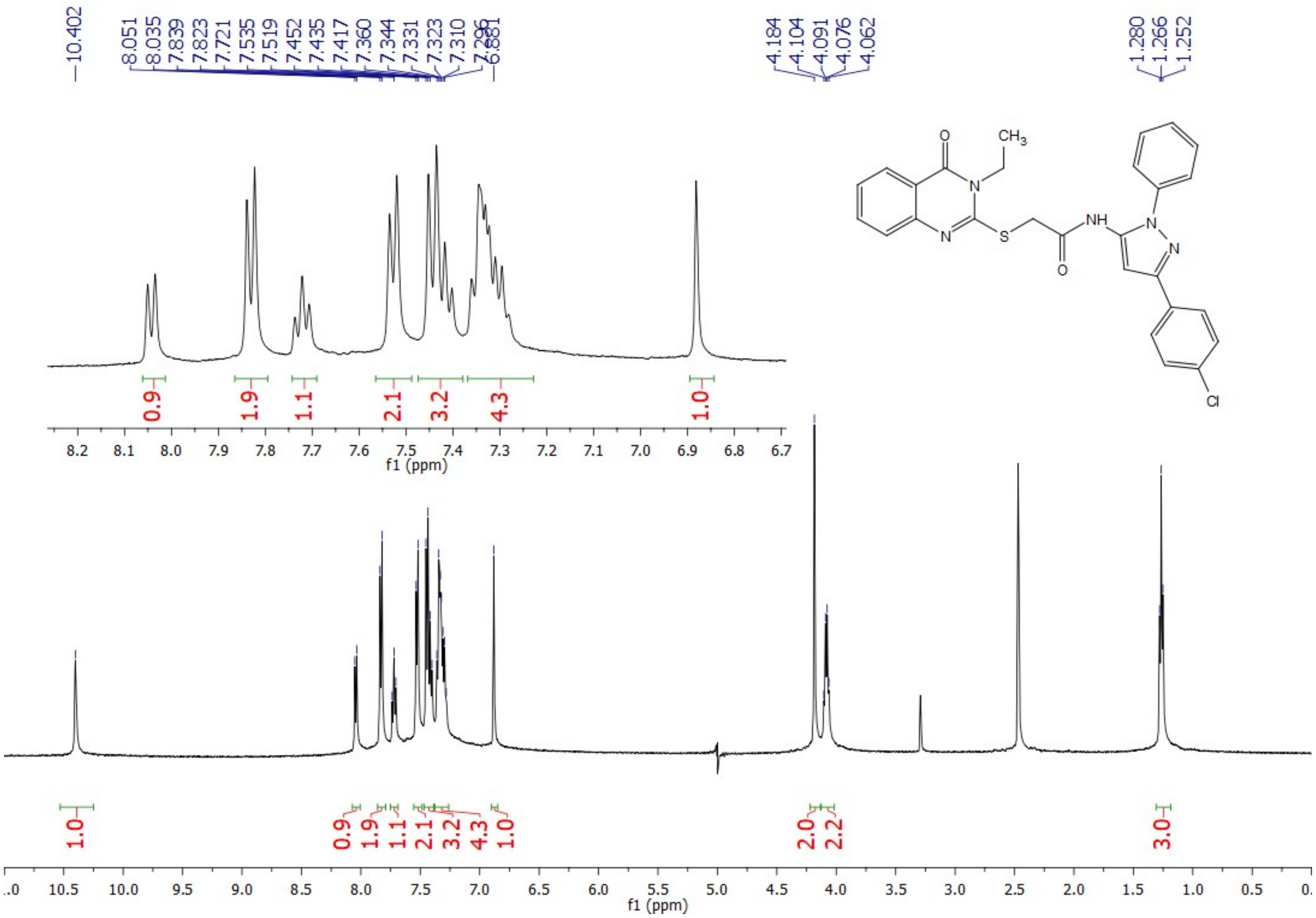

Figure S11. ^{1}H NMR spectrum of compound **7f**

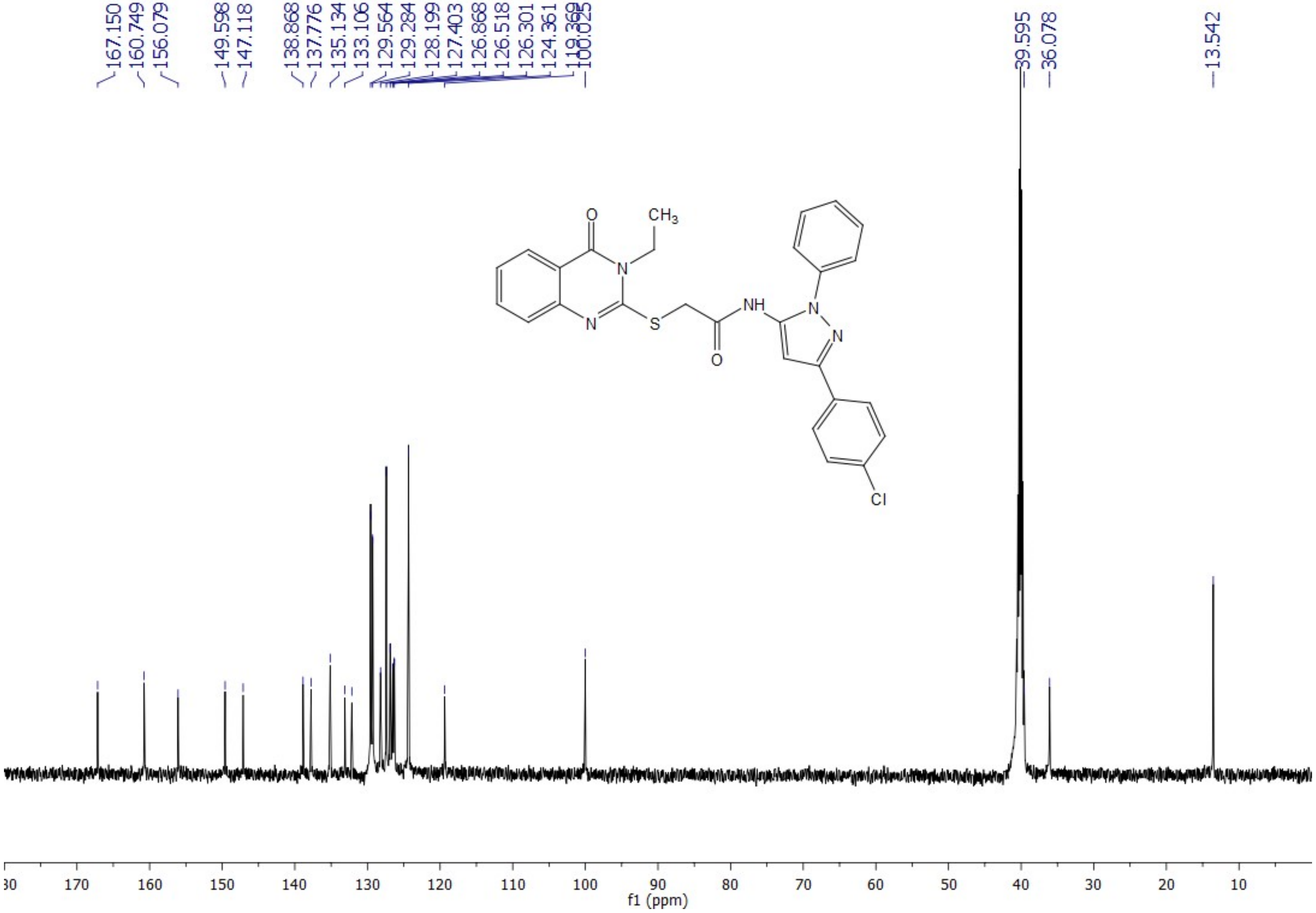

Figure S12. ^{13}C NMR spectrum of compound **7f**

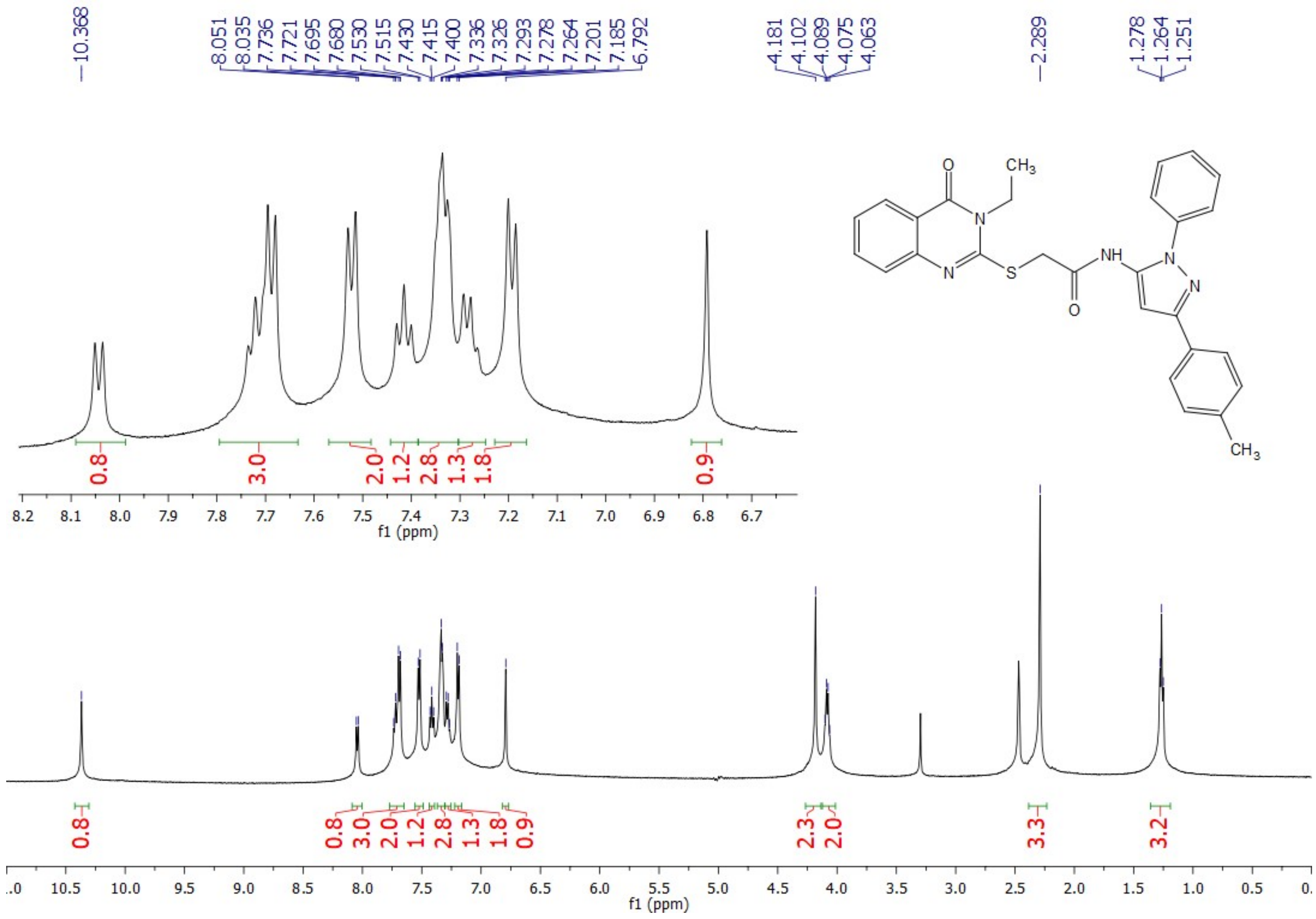

Figure S13. ^{1}H NMR spectrum of compound **7g**

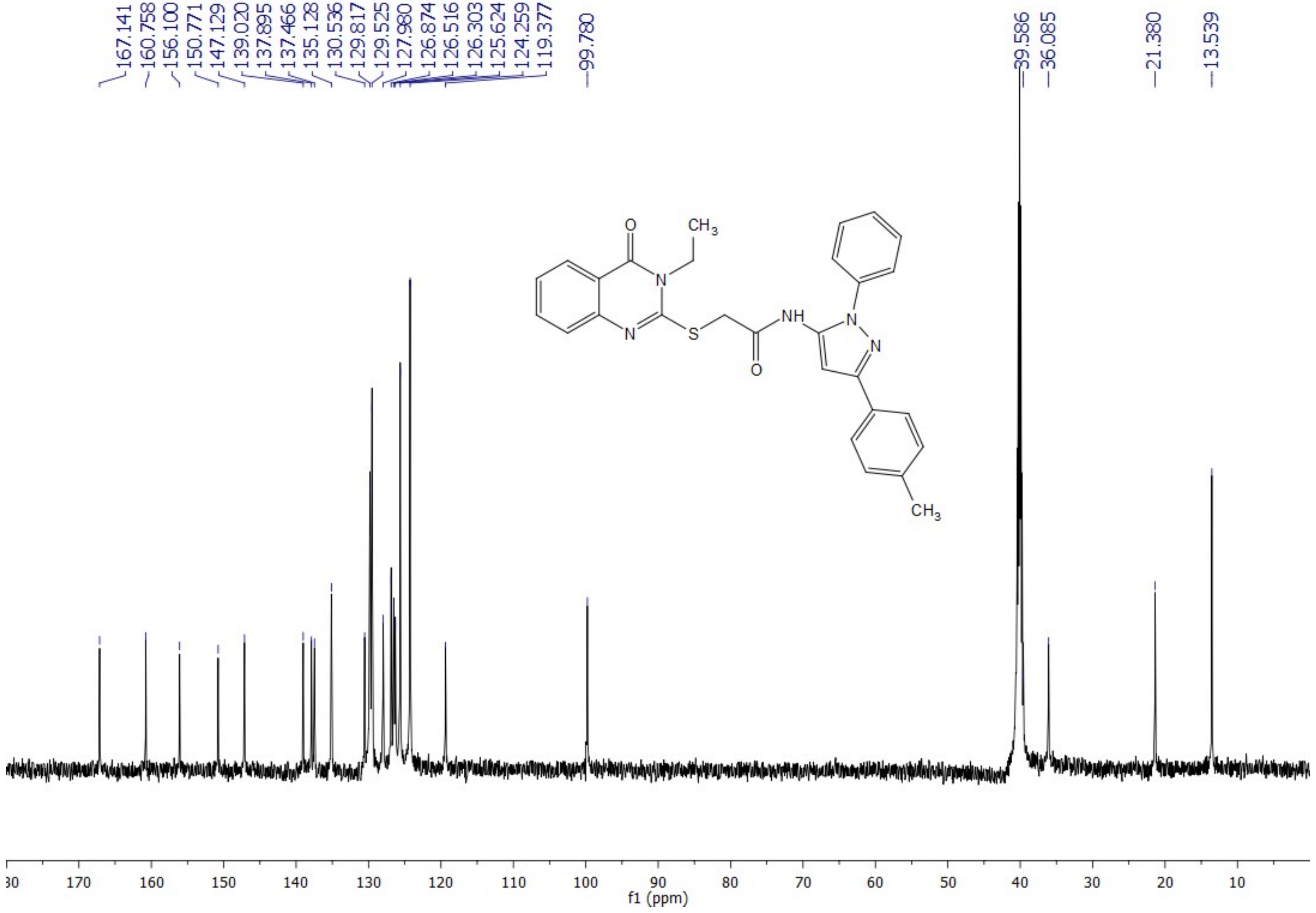

Figure S14. ^{13}C NMR spectrum of compound **7g**

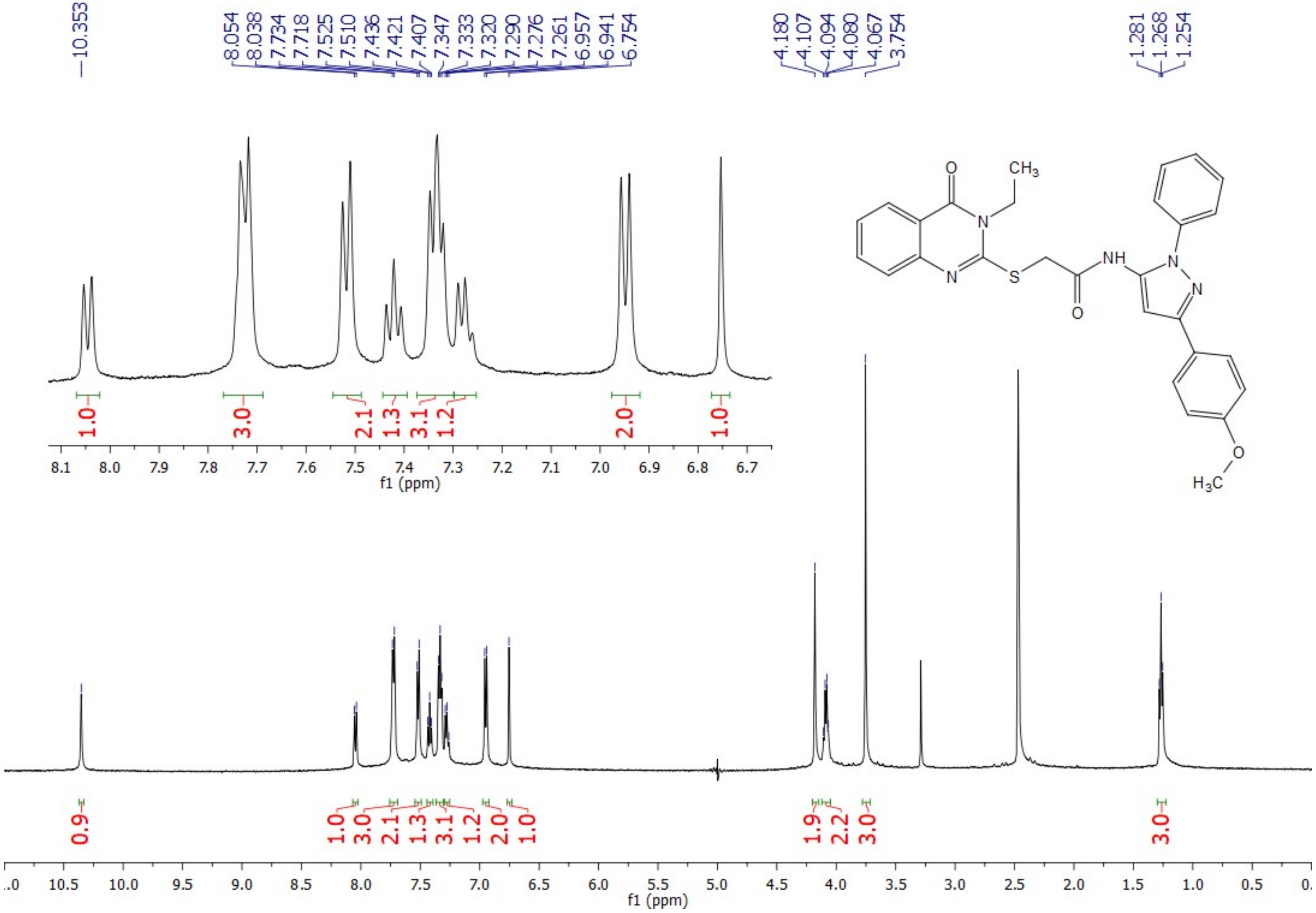

Figure S15. ^{1}H NMR spectrum of compound **7h**

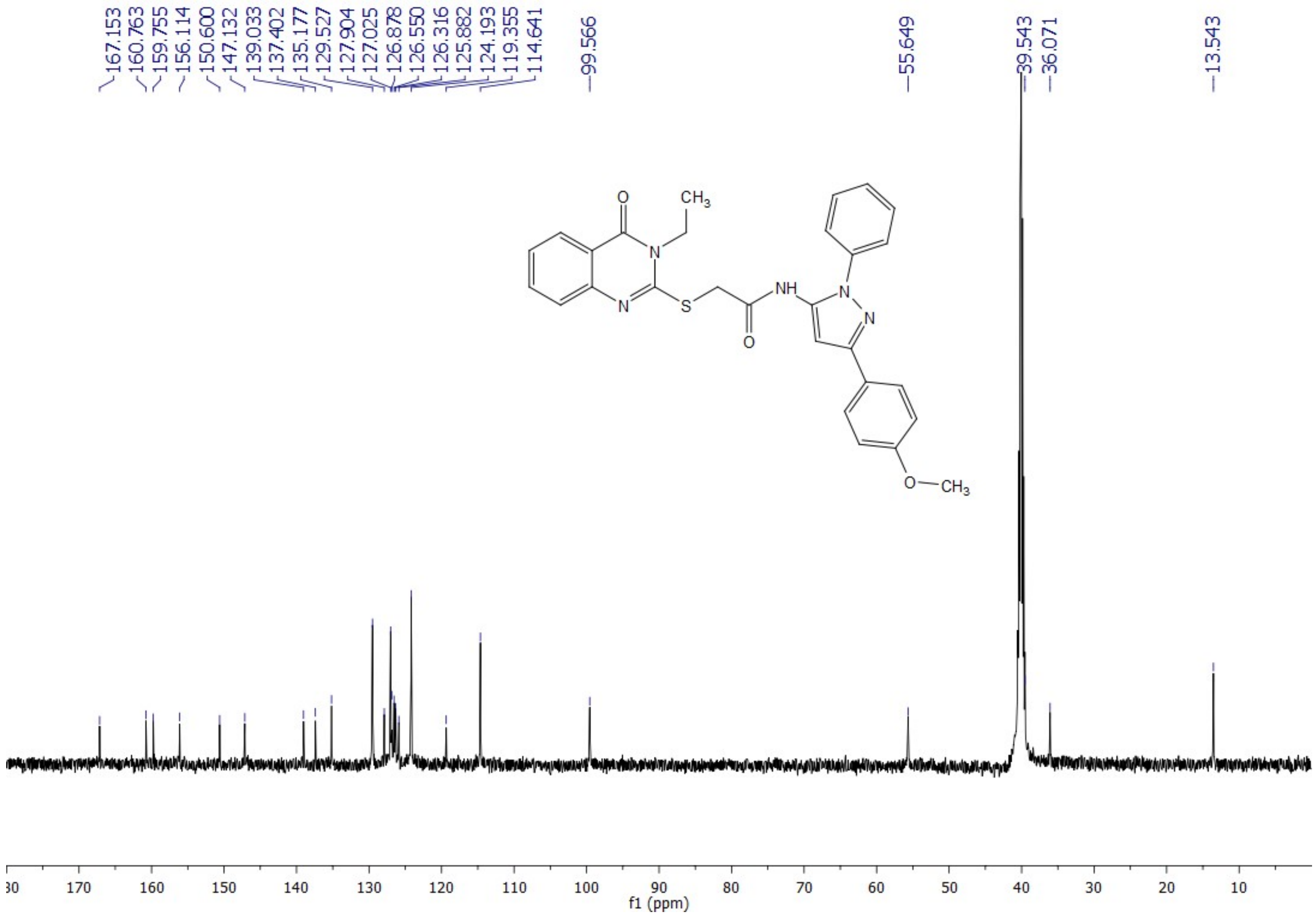

Figure S16. ^{13}C NMR spectrum of compound **7h**

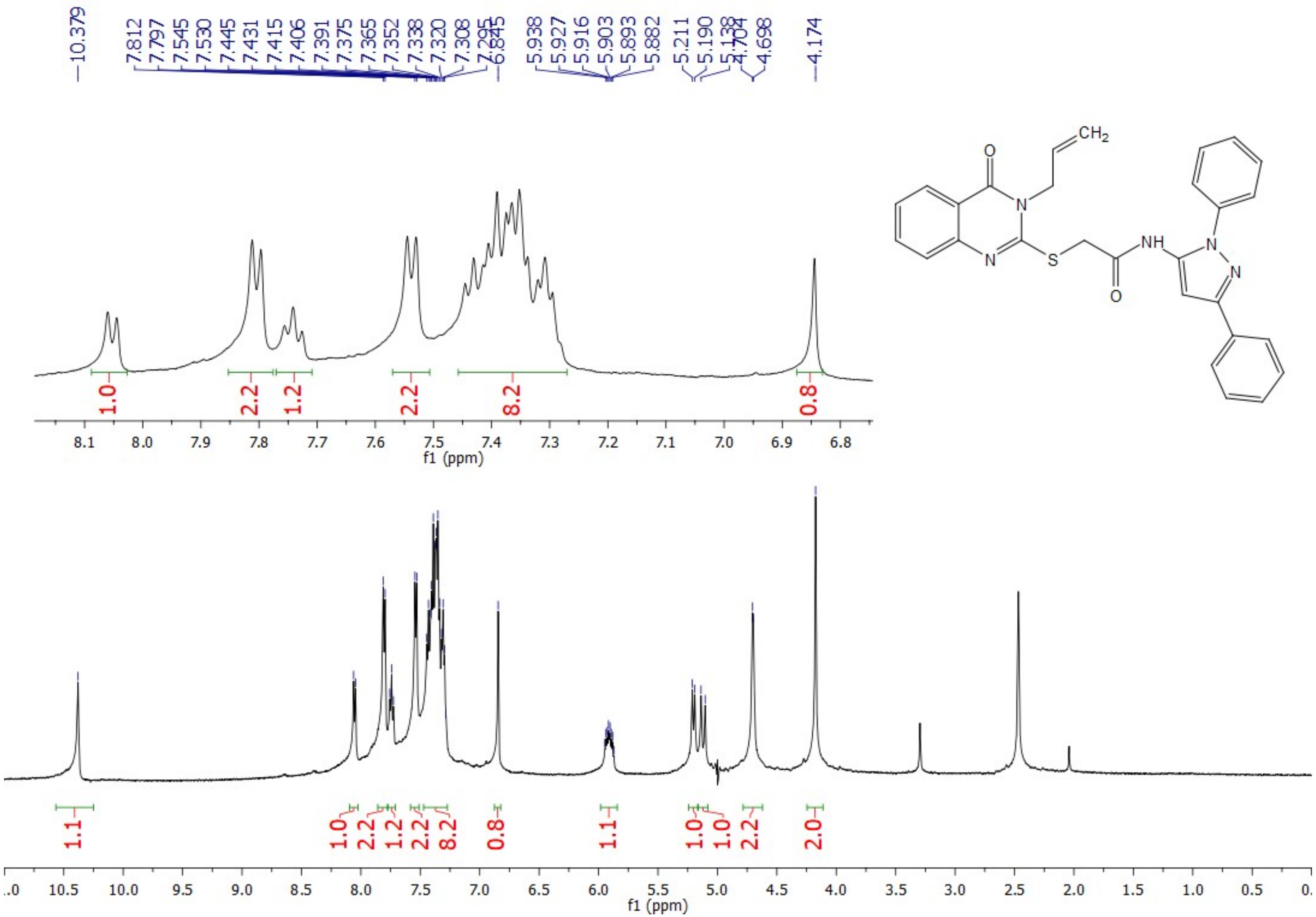

Figure S17. ^{1}H NMR spectrum of compound **7i**

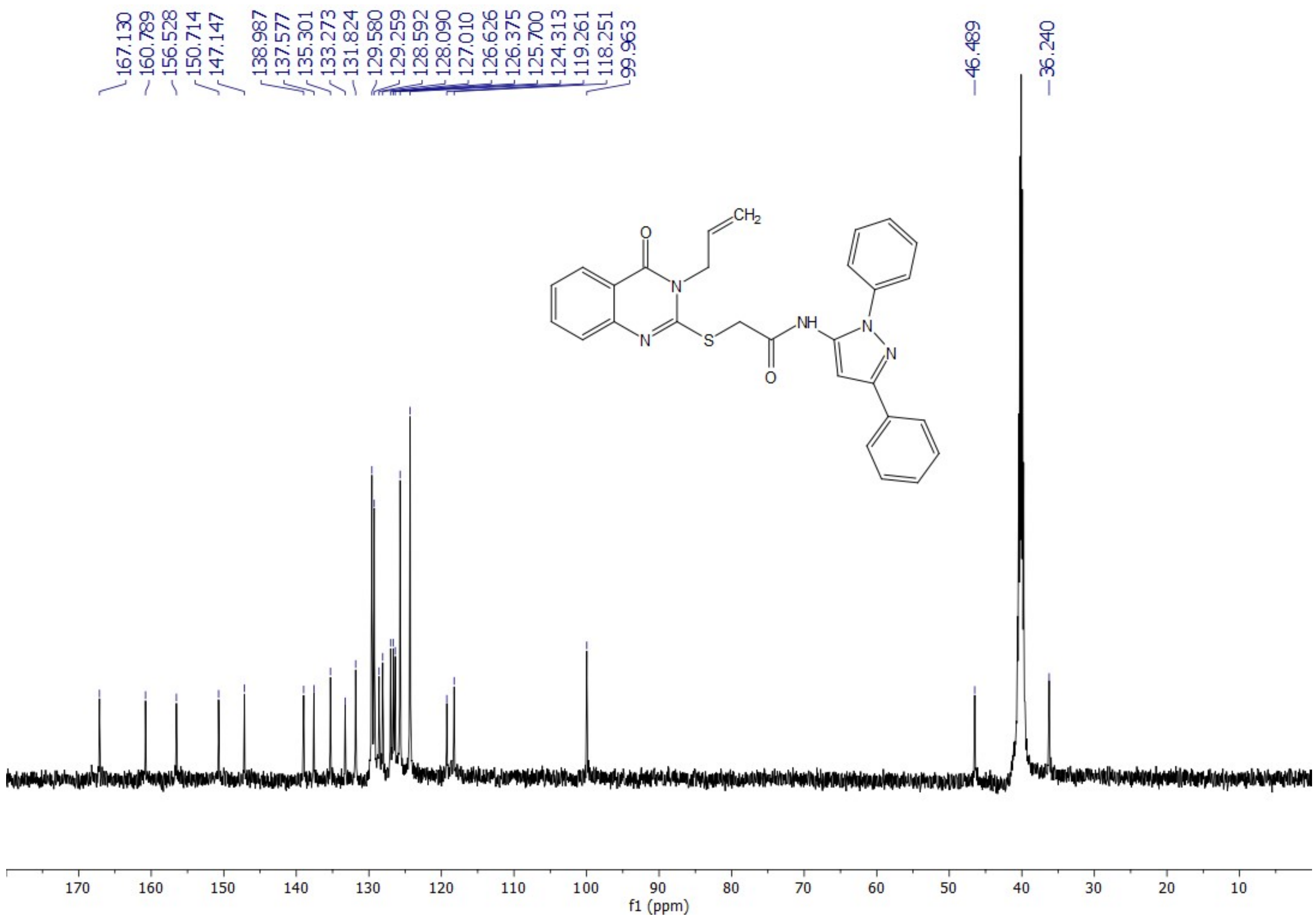

Figure S18. ^{13}C NMR spectrum of compound **7i**

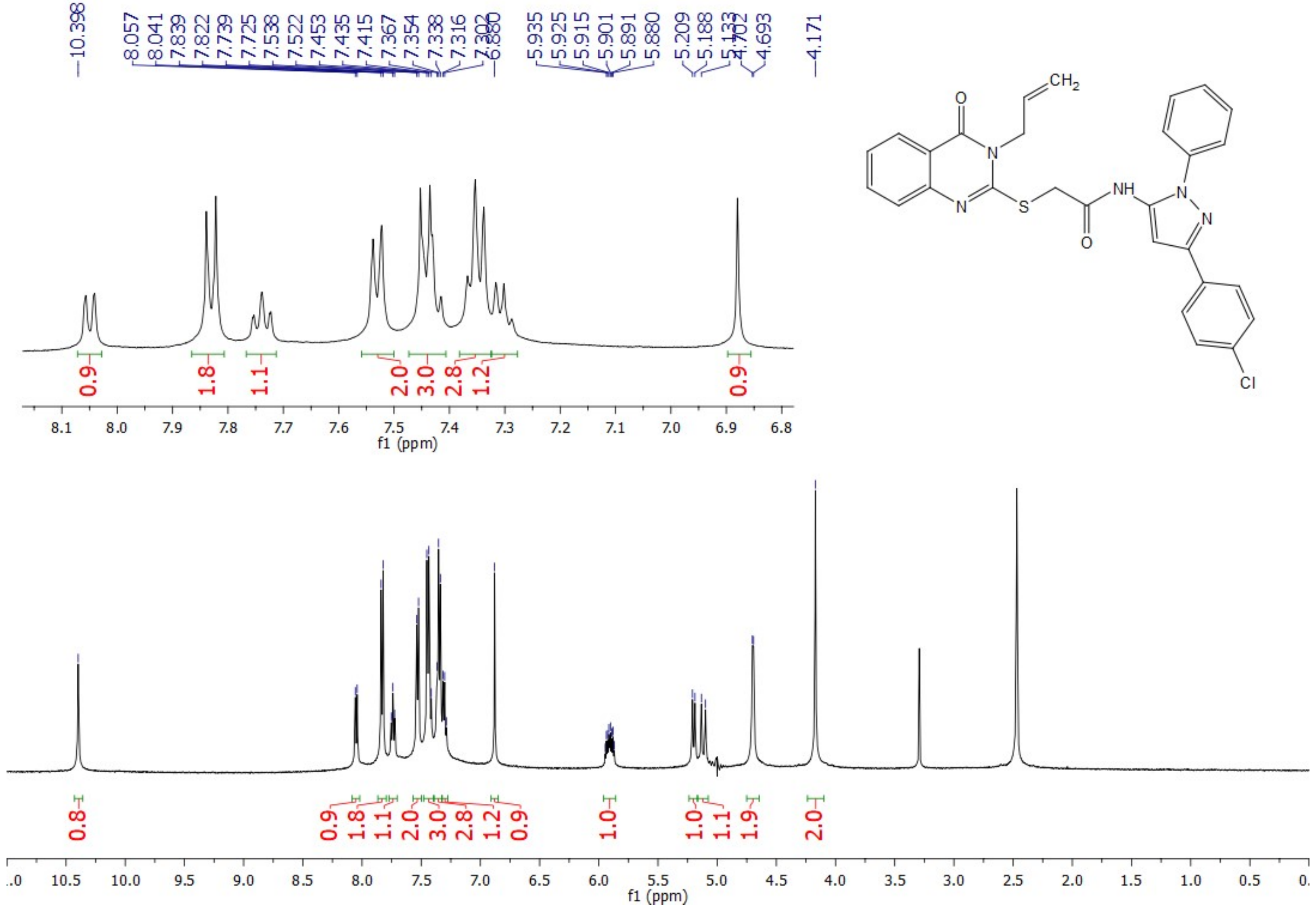

Figure S19. ^{1}H NMR spectrum of compound **7j**

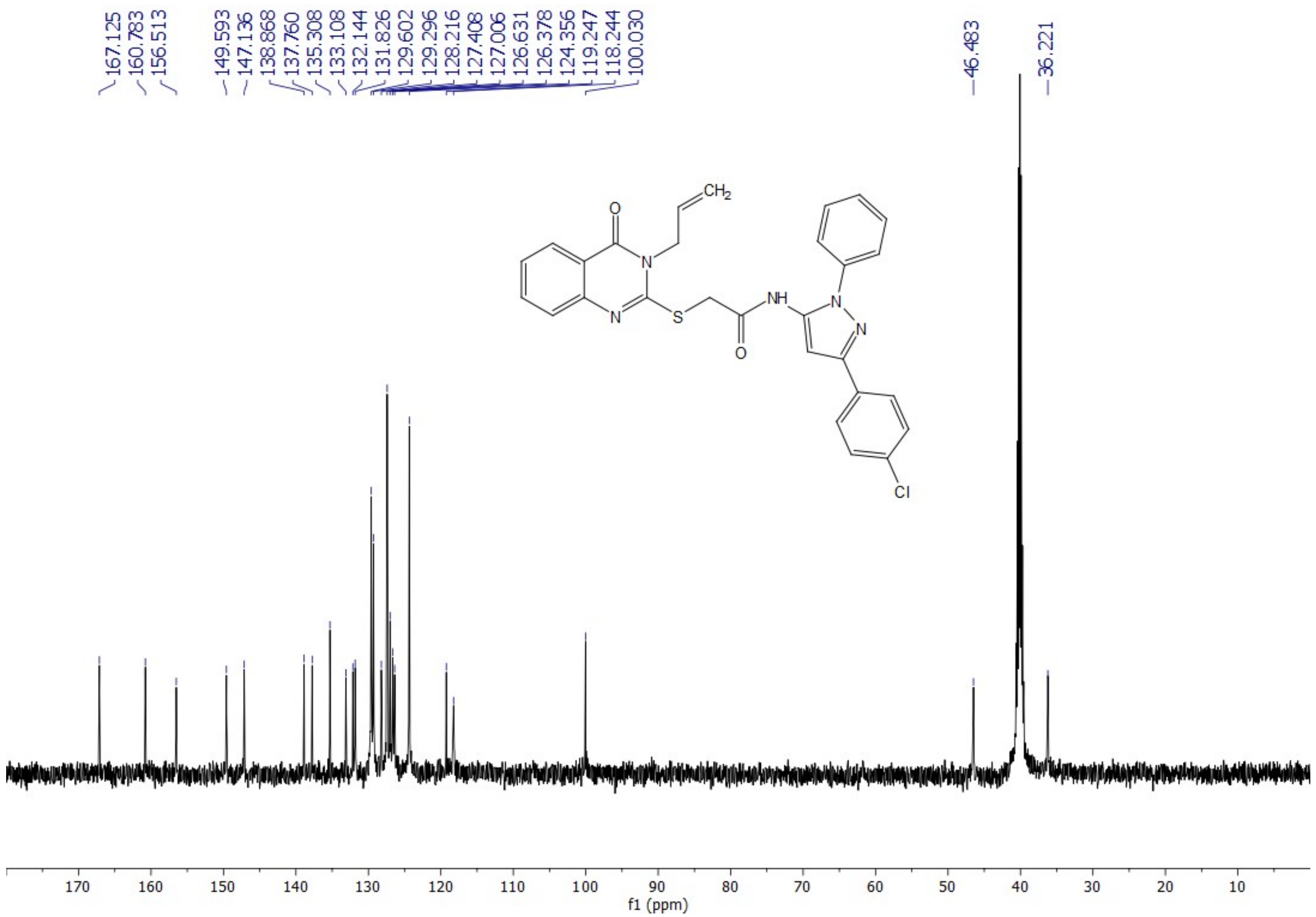

Figure S20. ^{13}C NMR spectrum of compound **7j**

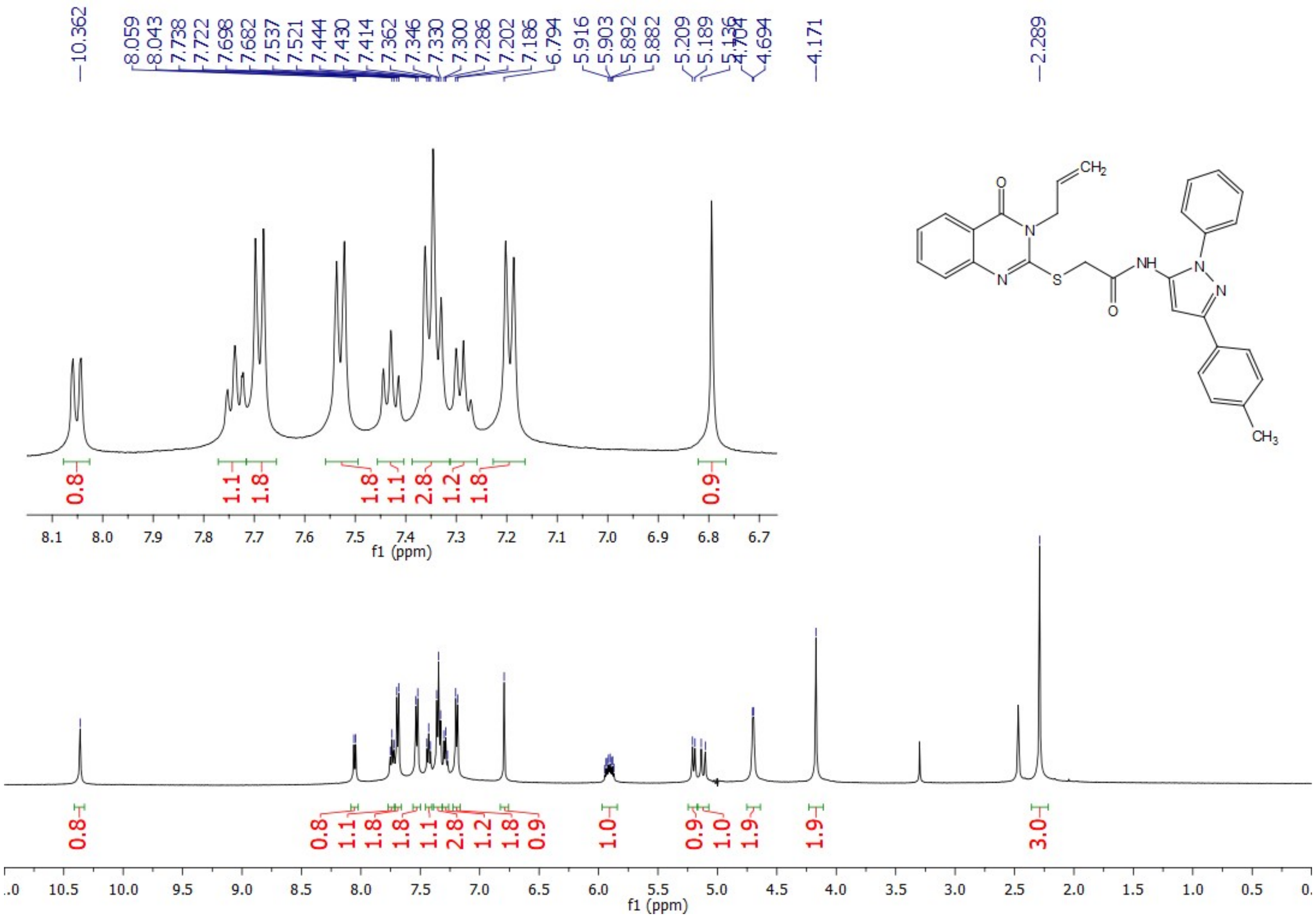

Figure S21. ^{1}H NMR spectrum of compound **7k**

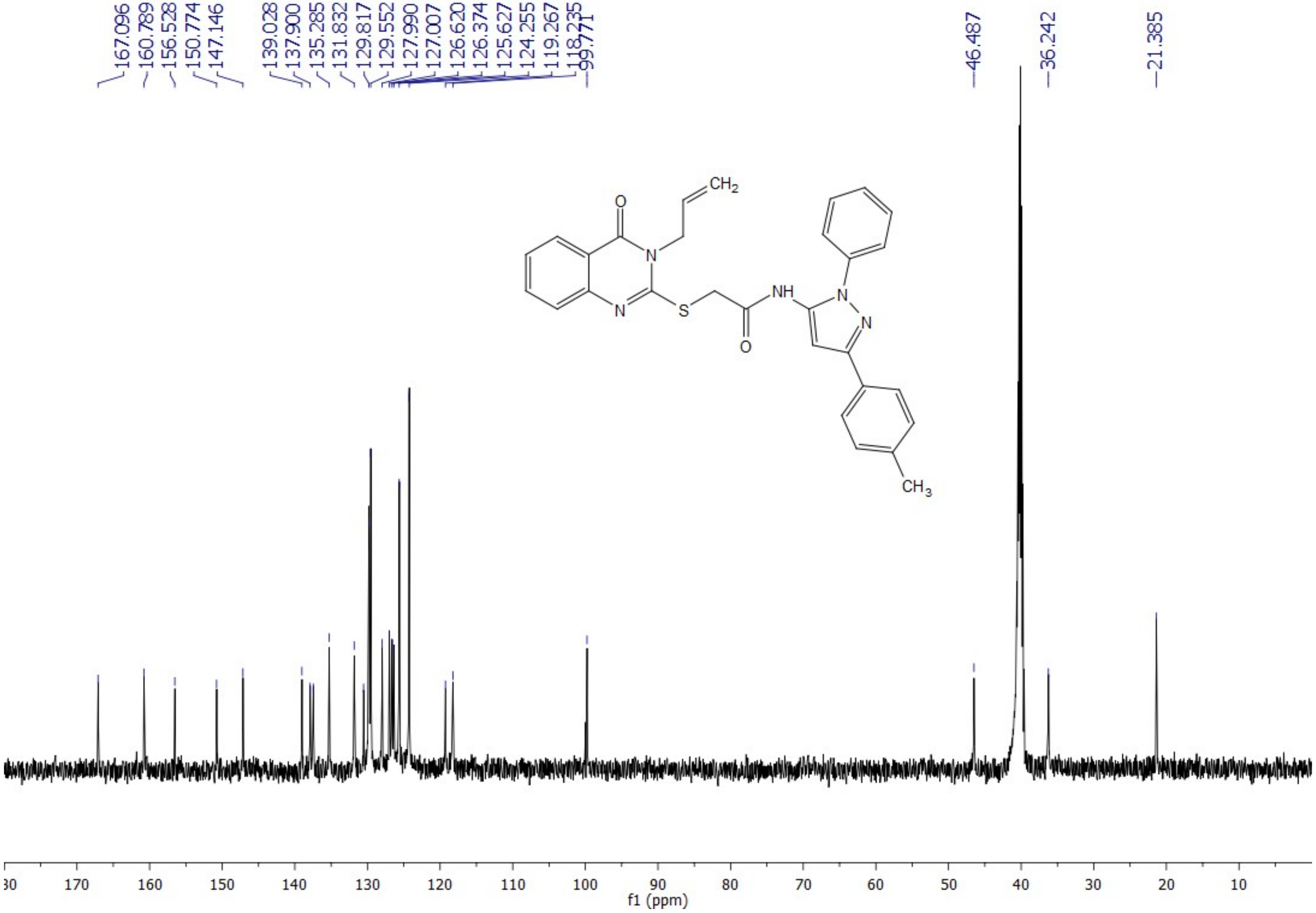

Figure S22. ^{13}C NMR spectrum of compound **7k**

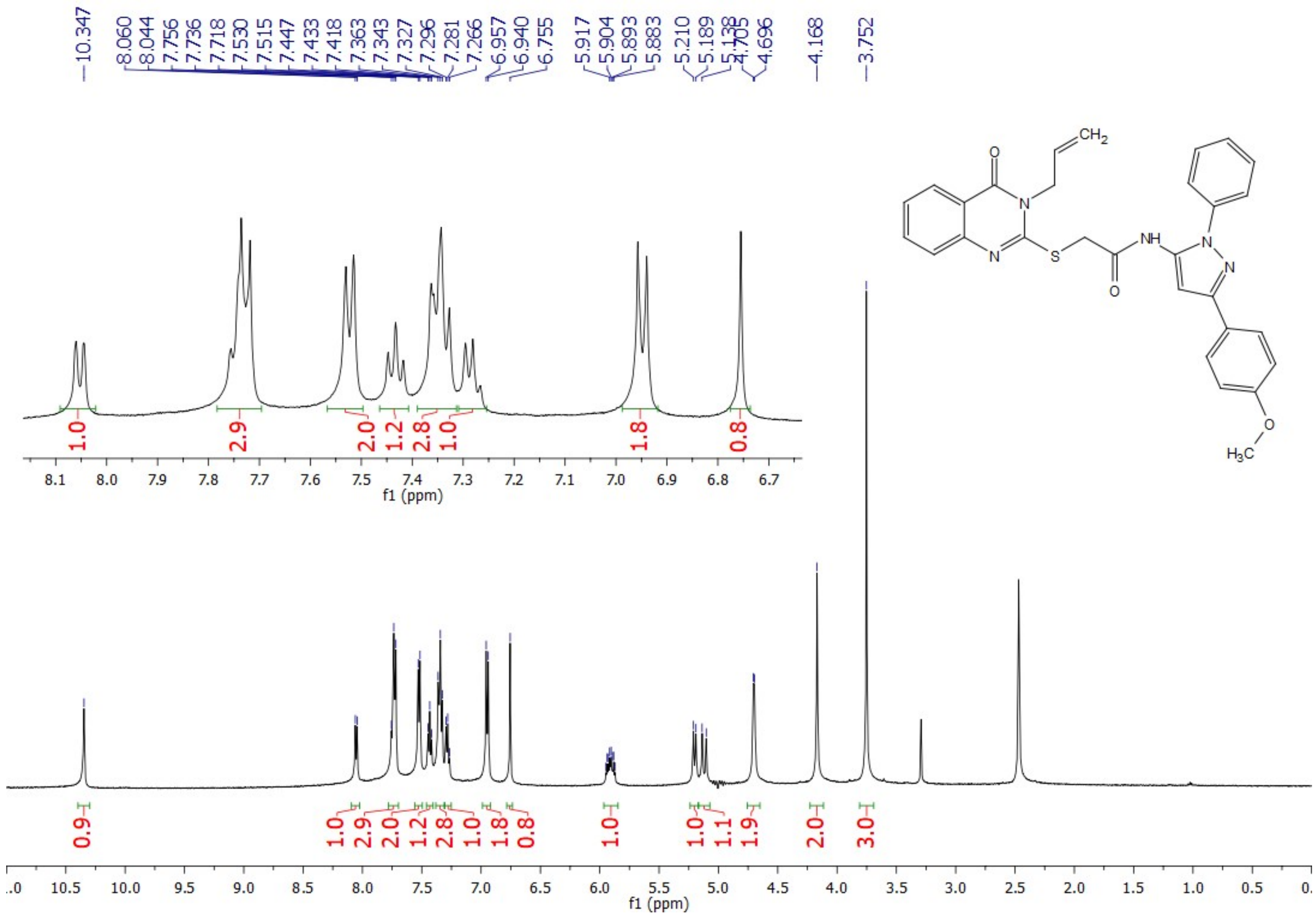

Figure S23. ^{1}H NMR spectrum of compound **7l**

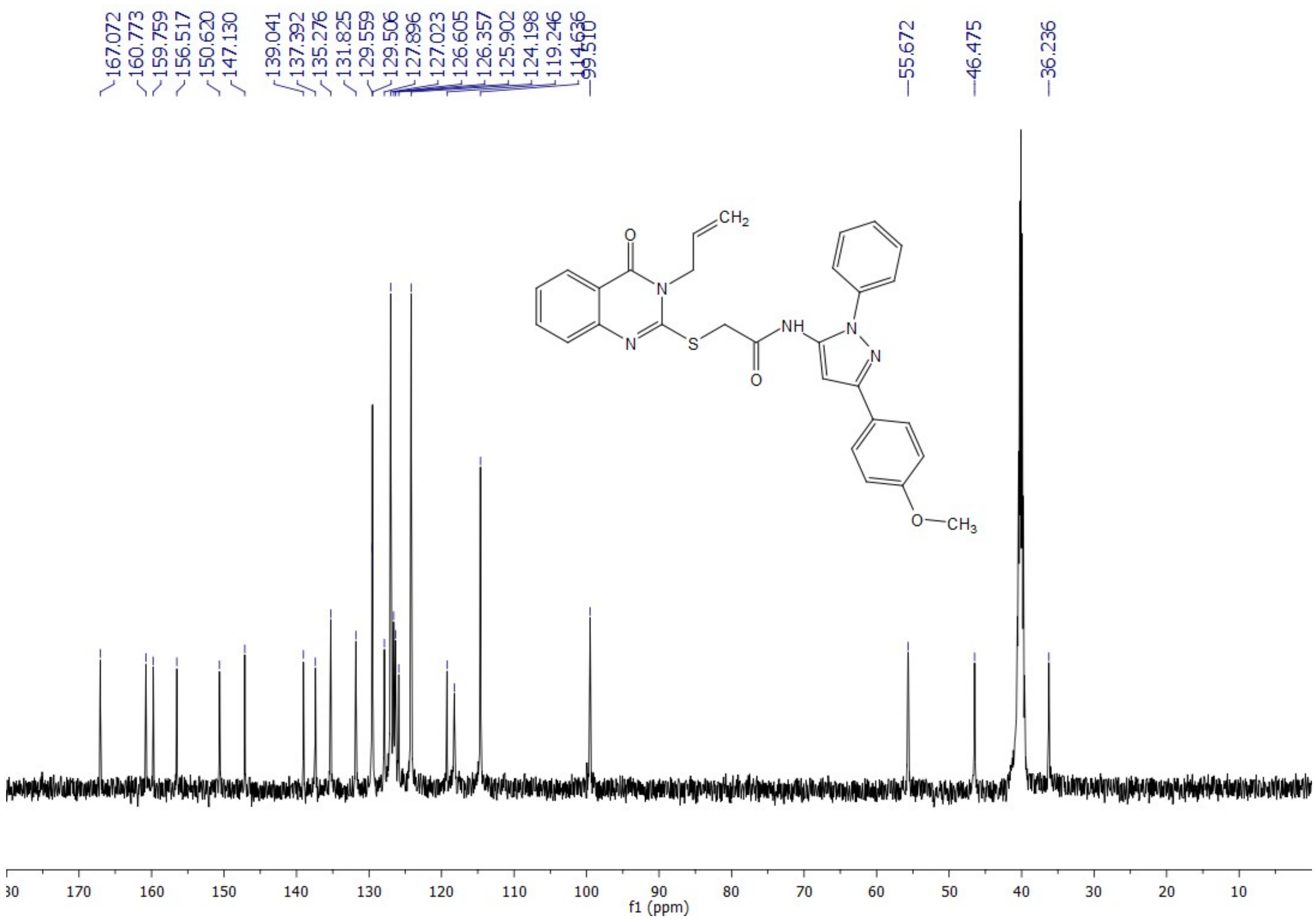

Figure S24. ^{13}C NMR spectrum of compound **7l**

2. Biology

2.1. EGFR inhibition assay

The EGFR inhibitory activities of compounds **7a–l** were determined using the ADP-Glo™ Kinase Assay + EGFR Kinase Enzyme System (Promega Corporation, Madison, WI, USA; Cat. No. V9261), with erlotinib employed as the reference inhibitor. The assay was performed in white low-volume 384-well plates using recombinant human EGFR kinase and Poly(Glu_4,Tyr_1) as the kinase substrate, following the manufacturer's recommended conditions.

Test compounds and erlotinib were prepared as serial dilutions covering a suitable concentration range for IC_{50} determination. Each 5 µL kinase reaction contained the test compound or vehicle, recombinant EGFR enzyme, substrate, and ATP. The reactions contained 4 ng EGFR per well, 0.2 µg/µL Poly(Glu_4,Tyr_1), and 5 µM ATP and were incubated at room temperature for 60 min. Vehicle-treated reactions containing enzyme, substrate, and ATP served as the uninhibited enzyme control, while appropriate background controls were included for correction of non-enzymatic luminescence.

Following incubation, 5 µL of ADP-Glo™ Reagent was added to each well to terminate the kinase reaction and deplete the remaining ATP, followed by incubation at room temperature for 40 min. Subsequently, 10 µL of Kinase Detection Reagent was added, and the plates were incubated for an additional 30 min at room temperature. The luminescence generated, which was proportional to the amount of ADP produced during the kinase reaction, was measured using a microplate luminometer.

The obtained luminescence values were corrected for background and normalized relative to the vehicle-treated enzyme control. Percentage EGFR inhibition was calculated for each tested concentration, and concentration–response curves were constructed. IC_{50} values, defined as the concentrations required to inhibit 50% of EGFR kinase activity, were determined by linear regression analysis using GraphPad Prism. Erlotinib was assayed under the same experimental conditions as the reference EGFR inhibitor.

2.2. COX inhibition assay

The inhibitory activities of compounds **7a–l** against COX-1 and COX-2 were determined using the COX (ovine/human) Inhibitor Screening Assay Kit (Cayman Chemical, Ann Arbor, MI, USA; Item No. 560131), containing ovine COX-1 and human recombinant COX-2. Celecoxib and indomethacin were employed as reference inhibitors. The assay was performed according to the manufacturer's recommended procedure using arachidonic acid as the COX substrate and $PGF_{2}\alpha$ production as the measure of enzyme activity.

Test compounds and reference inhibitors were prepared at serial concentrations suitable for IC_{50} determination. For each COX isoform, the enzyme was mixed with reaction buffer and heme, followed by addition of the tested inhibitor or the corresponding vehicle. The mixtures were preincubated for 10 min at 37 °C, after which the reactions were initiated by addition of arachidonic acid and allowed to proceed for exactly 2 min at 37 °C. Enzyme catalysis was terminated by addition of saturated stannous chloride solution, and the mixtures were incubated for a further 15 min at 37 °C to reduce the generated PGH_2 to the more stable $PGF_{2}\alpha$.

The amount of $PGF_{2}\alpha$ generated in each reaction was quantified using the competitive $PGF_{2}\alpha$ ELISA supplied with the kit. Appropriate dilutions of the COX reaction mixtures were added to the antibody-coated plate together with $PGF_{2}\alpha$ AChE tracer and $PGF_{2}\alpha$ antiserum and incubated for 18 h at 4 °C. The plate was subsequently washed, Ellman's Reagent was added, and color development was allowed to proceed in the dark. Absorbance was measured at 405–420 nm using a microplate reader.

$PGF_{2}\alpha$ concentrations were determined from the standard curve, and background values were subtracted from the corresponding enzyme activity measurements. Percentage inhibition was calculated relative to the vehicle-treated 100% initial activity control. IC_{50} values for COX-1 and COX-2 were determined from the concentration-dependent inhibitory responses by linear regression analysis using GraphPad Prism. COX-2 selectivity was expressed as the selectivity index (SI), calculated as COX-1 IC_{50} / COX-2 IC_{50}.

2.3. Antiproliferative activity

The antiproliferative activities of compounds **7a–l** were evaluated against A431 human epidermoid carcinoma, A549 human lung adenocarcinoma, HCA-7 human colorectal adenocarcinoma, and MRC-5 human lung fibroblast cells using the MTT-based *In Vitro*

Toxicology Assay Kit (Sigma-Aldrich, St. Louis, MO, USA; TOX1). Erlotinib and doxorubicin were employed as reference drugs. Cells were cultured under standard conditions, seeded into 96-well plates, and allowed to attach before treatment. The tested compounds and reference drugs were applied at serial concentrations covering suitable ranges for IC_{50} determination, while vehicle-treated cells served as the control.

Following the treatment period, freshly prepared MTT solution was added at a volume corresponding to 10% of the culture medium volume. The plates were incubated at 37 °C for 2–4 h to allow viable cells to reduce MTT to purple formazan crystals. MTT Solubilization Solution was then added at a volume equal to the original culture medium volume, and the plates were gently mixed until complete dissolution of the formed formazan.

Absorbance was measured at 570 nm using a microplate reader, with background absorbance measured at 690 nm and subtracted from the corresponding 570 nm values. Cell viability was calculated relative to the vehicle-treated control, which was considered to represent 100% viability, and percentage growth inhibition was determined for each tested concentration. IC_{50} values were calculated from the concentration-dependent responses by linear regression analysis using GraphPad Prism.

Cellular selectivity indices were calculated by dividing the MRC-5 IC_{50} value by the corresponding IC_{50} value against each cancer cell line. An overall selectivity index was additionally calculated as the ratio of the MRC-5 IC_{50} to the geometric mean IC_{50} obtained across A431, A549, and HCA-7 cells.

2.4. Cellular p-EGFR inhibition assay

The effects of compounds **7j, 7f, and 7k** on cellular EGFR phosphorylation were evaluated in A431 cells using the Human EGFR (pY1068) ELISA Kit (Abcam, Cambridge, UK; Cat. No. ab126438), with erlotinib employed as the reference inhibitor. Cells were treated with serial concentrations of the tested compounds or erlotinib and subsequently stimulated with recombinant human EGF (100 ng/mL) for 10 min to induce EGFR phosphorylation. Vehicle-treated, EGF-stimulated cells were used as the maximum phosphorylation control.

Following treatment, the cells were washed with PBS and lysed using the Cell Lysis Buffer supplied with the kit supplemented with protease and phosphatase inhibitors. Cell lysates were prepared at approximately 4×10^7 cells/mL and incubated with gentle shaking at 2–8 °C for 30 min. The lysates were then centrifuged at 13,000 rpm for 10 min at 2–8 °C, and the clarified supernatants were collected for analysis.

Aliquots (100 µL) of the prepared cell lysates were transferred to the anti-EGFR antibody-coated microplate and incubated for 2.5 h at room temperature with gentle shaking. The wells were washed four times with the supplied Wash Buffer, followed by addition of 100 µL of the prepared anti-phospho-EGFR (Y1068) detection antibody and incubation for 1.5 h at room temperature. After washing, 100 µL of HRP-conjugated anti-rabbit IgG was added and the plate was incubated overnight at 4 °C with gentle shaking.

The wells were washed four times, after which 100 µL of TMB One-Step Substrate Reagent was added and the plate was incubated for 30 min at room temperature in the dark. The reaction was terminated by addition of 50 µL of Stop Solution, and absorbance was measured immediately at 450 nm using a microplate reader.

After subtraction of the blank absorbance, the p-EGFR(Y1068) signal in each treated sample was expressed relative to that of the EGF-stimulated vehicle control, which was considered to represent 100% phosphorylation. Percentage inhibition of cellular EGFR phosphorylation was calculated for each tested concentration, and IC_{50} values were determined from the concentration-dependent responses by linear regression analysis using GraphPad Prism. As total EGFR was not measured, the results were expressed as changes in the p-EGFR(Y1068) signal rather than as p-EGFR/total EGFR ratios.

2.5. Effects on cellular PGE_2 production

The effects of compounds **7f, 7j, and 7k** on cellular PGE_2 production were determined using the Parameter™ Prostaglandin E_2 Immunoassay (R&D Systems, Minneapolis, MN, USA; Cat. No. KGE004B), a forward sequential competitive enzyme immunoassay for quantitative determination of PGE_2 in cell-culture supernatants. Celecoxib was employed as the reference inhibitor. Cells were treated with compounds **7f, 7j, and 7k** at 1.0 µM, while celecoxib was tested at 50 µM. Untreated and vehicle-treated cells were included as controls. Following the treatment period, culture supernatants were collected and centrifuged to remove particulate material.

Prior to analysis, the collected supernatants were diluted threefold with Calibrator Diluent RD5-56 according to the manufacturer's instructions. Aliquots (150 μL) of standards or diluted samples were added to the appropriate wells, followed by 50 μL of Primary Antibody Solution. The plate was incubated for 1 h at room temperature on a horizontal orbital microplate shaker at 500 ± 50 rpm. Without an intermediate washing step, 50 μL of PGE_2 Conjugate was added to each well, followed by incubation for a further 2 h under the same conditions.

The wells were subsequently aspirated and washed four times with Wash Buffer, after which 200 μL of Substrate Solution was added. The plate was incubated for 30 min at room temperature protected from light, and the reaction was terminated by addition of 100 μL of Stop Solution. Absorbance was measured at 450 nm using a microplate reader, with wavelength correction at 540 or 570 nm. PGE_2 concentrations were determined from the corresponding standard curve and multiplied by the sample dilution factor to obtain the concentrations in the original culture supernatants.

PGE_2 levels were expressed in ng/mL. Fold change and relative PGE_2 production were calculated relative to the untreated control, which was assigned values of 1.00-fold and 100%, respectively. Inhibition of PGE_2 release was calculated as 100 minus the relative PGE_2 production (%).

2.6. Effect on Bax and Bcl-2 levels

The effects of compound **7f** on the cellular levels of the pro-apoptotic protein Bax and anti-apoptotic protein Bcl-2 were determined using the DRG Human Bax ELISA Kit (DRG International, Inc., USA; Cat. No. EIA-4487) and Zymed Bcl-2 ELISA Kit (Invitrogen, Carlsbad, CA, USA; Cat. No. 99-0042), respectively. Doxorubicin was employed as the reference drug, while vehicle-treated cells served as the control. Following treatment, cells were collected, washed with PBS, and lysed. The clarified cell lysates were used for the respective ELISA measurements according to the manufacturers' recommended procedures.

For Bax determination, appropriate volumes of assay buffer, Bax standards, and cell lysates were added to the designated wells of the antibody-coated microplate and incubated for 60 min at room temperature with shaking. After washing, the Bax detection antibody was added and the plate was incubated for a further 60 min. Following another washing step, enzyme conjugate was added and incubated for 30

min. The wells were washed, substrate solution was added, and color development was allowed to proceed for 30 min at room temperature. The reaction was terminated with Stop Solution, and absorbance was measured using a microplate reader.

For Bcl-2 determination, appropriately diluted standards and cell lysates were added to the microplate together with the biotin-conjugated anti-Bcl-2 antibody and incubated for 2 h. After washing, streptavidin-HRP solution was added and the plate was incubated for 1 h. The wells were subsequently washed, TMB substrate solution was added for color development, and the reaction was terminated with Stop Solution. Absorbance was measured at 450 nm using a microplate reader.

Bax and Bcl-2 concentrations were calculated from their respective standard curves and expressed as pg μL^{-1}. Fold changes were calculated relative to the vehicle-treated control. The Bax/Bcl-2 ratio was calculated by dividing the Bax concentration by the corresponding Bcl-2 concentration for each treatment group.

2.7. Effect on caspase-3 and caspase-9 levels

The effects of compound **7f** on active caspase-3 and caspase-9 levels were evaluated using the Human Caspase-3 (Active) ELISA Kit (Invitrogen, Thermo Fisher Scientific, USA; Cat. No. KHO1091) and Human Caspase-9 ELISA Kit (Thermo Fisher Scientific, USA; Cat. No. BMS2025), respectively. Doxorubicin was employed as the reference drug, while vehicle-treated cells served as the control. Following treatment, cells were harvested, washed with PBS, and lysed according to the respective manufacturers' procedures. Clarified cell lysates were used for ELISA analysis.

For active caspase-3 determination, cells were lysed in Cell Extraction Buffer on ice for 30 min, with vortexing at 10-min intervals. The lysates were centrifuged at 13,000 rpm for 10 min at 4 °C, and the resulting supernatants were appropriately diluted with Standard Diluent Buffer. Aliquots (100 µL) of standards and samples were added to the antibody-coated wells and incubated for 2 h at room temperature. After four washes, 100 µL of Caspase-3 (Active) Detection Antibody was added and incubated for 1 h. The wells were washed four times, followed by addition of 100 µL of anti-rabbit IgG-HRP and incubation for 30 min. After washing, 100 µL of Stabilized Chromogen was added and the plate was incubated for 30 min in the dark. The reaction was terminated with 100 µL of Stop Solution, and absorbance was measured at 450 nm. Active caspase-3 concentrations were obtained from the corresponding standard curve.

For caspase-9 determination, cell lysates were prepared using the supplied Lysis Buffer and clarified by centrifugation. Samples were added in duplicate to the antibody-coated wells together with Sample Diluent and Detection Antibody and incubated for 2 h at room temperature. The wells were washed three times, followed by addition of 100 µL of diluted anti-rabbit IgG-HRP and incubation for 1 h. After three additional washes, 100 µL of TMB Substrate Solution was added and the plate was incubated for approximately 10 min at room temperature. The reaction was stopped with 100 µL of Stop Solution, and absorbance was measured at 450 nm. Caspase-9 concentrations were calculated from the corresponding standard curve, taking the assay dilution factor into account.

Active caspase-3 levels were expressed as pg/mL and caspase-9 levels as ng/mL. Fold changes were calculated relative to the vehicle-treated control, which was assigned a value of 1.00.

2.8. Molecular docking

Molecular docking methodology

Molecular docking calculations were performed using AutoDock Vina. The crystal structures of wild-type EGFR kinase domain co-crystallized with gefitinib (PDB ID: 4WKQ) and COX-2 co-crystallized with SC-558 (PDB ID: 1CX2) were retrieved from the RCSB Protein Data Bank.

Protein and ligand preparation was performed using AutoDockTools. The co-crystallized ligands were separated from their respective protein structures and retained for validation of the docking protocol. Water molecules were removed, polar hydrogen atoms were added, and appropriate partial charges and AutoDock atom types were assigned. The prepared receptors were saved in PDBQT format. The structure of **7f** and the co-crystallized ligands were similarly prepared by addition of hydrogens and partial charges, assignment of rotatable bonds, and conversion to PDBQT format. The receptors were treated as rigid, whereas ligand rotatable bonds were allowed conformational flexibility during docking.

The docking search spaces were defined around the binding sites occupied by the respective co-crystallized ligands. For EGFR (4WKQ), the search box was centered at $x = 1.45748$, $y = 194.208$, and $z = 20.4128$ Å, with dimensions of 30 × 30 × 30 Å. For

COX-2 (1CX2), the search box was centered at x = 21.583, y = −3.655, and z = 36.436 Å, with dimensions of 26 × 26 × 26 Å. These search spaces encompassed the corresponding gefitinib- and SC-558-binding regions.

Prior to docking **7f**, the docking protocols were validated by re-docking the respective co-crystallized ligands into their original binding sites. Gefitinib was re-docked into the EGFR ATP-binding site, whereas SC-558 was re-docked into the COX-2 binding pocket. The resulting poses were superimposed on their corresponding crystallographic conformations, and root-mean-square deviation (RMSD) values were calculated to evaluate reproduction of the experimental binding orientations.

Following validation, **7f** was docked into the EGFR and COX-2 binding sites using the corresponding validated search spaces. The generated poses were ranked according to the predicted AutoDock Vina binding affinity, expressed in kcal/mol, and the selected poses were evaluated according to their orientation and interactions within the respective binding pockets. Protein–ligand interactions and two- and three-dimensional representations of the selected complexes were analyzed using BIOVIA Discovery Studio Visualizer.
